# Structural characterization of the supra­molecular complex between a tetra­quinoxaline-based cavitand and benzo­nitrile

**DOI:** 10.1107/S205698902400481X

**Published:** 2024-05-31

**Authors:** Roberta Pinalli, Chiara Massera

**Affiliations:** a Dipartimento di Scienze Chimiche, della Vita e della Sostenibilità Ambientale, Università di Parma, Parco Area delle Scienze 17/A, 43124 Parma, Italy; Universidad de Los Andes, Venezuela

**Keywords:** crystal structure, quinoxaline cavitands, inclusion compounds, benzo­nitrile

## Abstract

The structural characterization is reported of the supra­molecular complex between the tetra­quinoxaline-based cavitand 2,8,14,20-tetra­hexyl-6,10:12,16:18,22:24,4-*O*,*O*′-tetra­kis­(quinoxaline-2,3-di­yl)calix[4]resorcinarene (**QxCav**) with benzo­nitrile. The complex, of general formula C_84_H_80_N_8_O_8_·2C_7_H_5_N, crystallizes in the space group *P*  with two independent mol­ecules in the asymmetric unit, displaying very similar geometrical parameters. For each complex, one of the benzo­nitrile mol­ecules is engulfed inside the cavity, while the other is located among the alkyl legs at the lower rim. The host and the guests mainly inter­act through weak C—H⋯π, C—H⋯N and dispersion inter­actions. These inter­actions help to consolidate the formation of supra­molecular chains running along the crystallographic *b-*axis direction.

## Abstract 2

The 2:1 supra­molecular complex between a tetra­quinoxaline-based cavitand and benzo­nitrile as a guest has been studied through X-ray diffraction analysis. One of the benzo­nitrile mol­ecules in engulfed inside the macrocycle.

## 1. Chemical context

Quinoxaline cavitands (QxCavs), initially reported by Cram and co-workers (Moran *et al.*, 1982 ▸), have been extensively studied in the past years for their mol­ecular recognition properties. These macrocycles are obtained by bridging four times a resorcinarene scaffold with 2,3-di­chloro­quinoxaline derivatives, affording a deep cavity capable of engulfing aromatic guests both in solution (Giannetto *et al.*, 2018 ▸) and in the gas phase (Vincenti *et al.*, 1993 ▸; Clément *et al.*, 2015 ▸; Trzciński *et al.*, 2017 ▸). The driving forces for the formation of these host–guest complexes are non-covalent C—H⋯π and π–π inter­actions that are established between the receptor and the included aromatic compound (Soncini *et al.*, 1992 ▸). Another peculiar feature of these cavitands is their ability to reversibly switch between two spatially well-defined conformations. By reorganizing the four 1,4-di­aza­naphthalene ‘flaps’ from equatorial to axial positions, these cavitands can reversibly inter­convert between an expanded *kite* (*C*_2*v*_ symmetry) and a contracted *vase* (*C*_4*v*_ symmetry) form (Azov *et al.*, 2006 ▸). All inter­mediate conformers being energetically disfavoured, this mol­ecular switching involves two discrete states and can be triggered in solution by different stimuli, such as pH and temperature variation (Skinner *et al.*, 2001 ▸; Moran *et al.*, 1991 ▸), Zn^2+^ coordination (Frei *et al.*, 2004 ▸) and redox reactions (Pochorovski & Diederich, 2014 ▸). With their ability to close and open upon external stimulation, QxCavs can be used to grab and release mol­ecules, acting as mol­ecular grippers (Milić & Diederich, 2019 ▸). By incorporating these gripper-like cavitands in polymers, the pH-driven conformational switch is maintained (Brighenti *et al.*, 2018 ▸) and can be used to regenerate QxCav-based membranes for the removal of polycyclic aromatic hydro­carbons from water under relatively mild conditions (Amorini *et al.*, 2022 ▸). By covalently embedding QxCavs in polymeric matrices, indeed, the vase–kite switching can be controlled by mechanical stimulation (Torelli *et al.*, 2020 ▸), leading to the unprecedented observation of an auxetic behaviour in a polymer of intrinsic microporosity (PIMs; Portone *et al.*, 2023 ▸). The extensive versatility of these cavitands arises from the accessible functionalization of both the lower rim of the resorcinarene unit and the quinoxaline bridges. The introduction of positively charged groups on the cavitand feet, for example, was found to be a powerful tool to impart water solubility to quinoxaline-like cavitands (Zhu *et al.*, 2022 ▸), while the insertion of a carboxyl group at the upper rim enhanced the selectivity of QxCav toward nitro­aromatic volatile compounds by adding additional hydrogen-bonding inter­actions with the NO_2_ group of the guest (Bianchi *et al.*, 2014 ▸). As a continuation of our studies towards optimal sensors for environmental applications, we have probed the recognition ability of 2,8,14,20-tetra­hexyl-6,10:12,16:18,22:24,4-*O*,*O*′-tetra­kis­(quinoxaline-2,3-di­yl)calix[4]resorcinarene (**QxCav**) towards benzo­nitrile. Benzo­nitrile has already been used as a guest in the conformationally vase-blocked resorcinarene cavitand EtQxBox to probe its effect on the cavitand fluorescence (Aprile *et al.*, 2018 ▸). Quenching was demonstrated through steady-state emission analysis. In this paper, we report and analyse the crystal structure of the supra­molecular host–guest complex between **QxCav** and benzo­nitrile. 
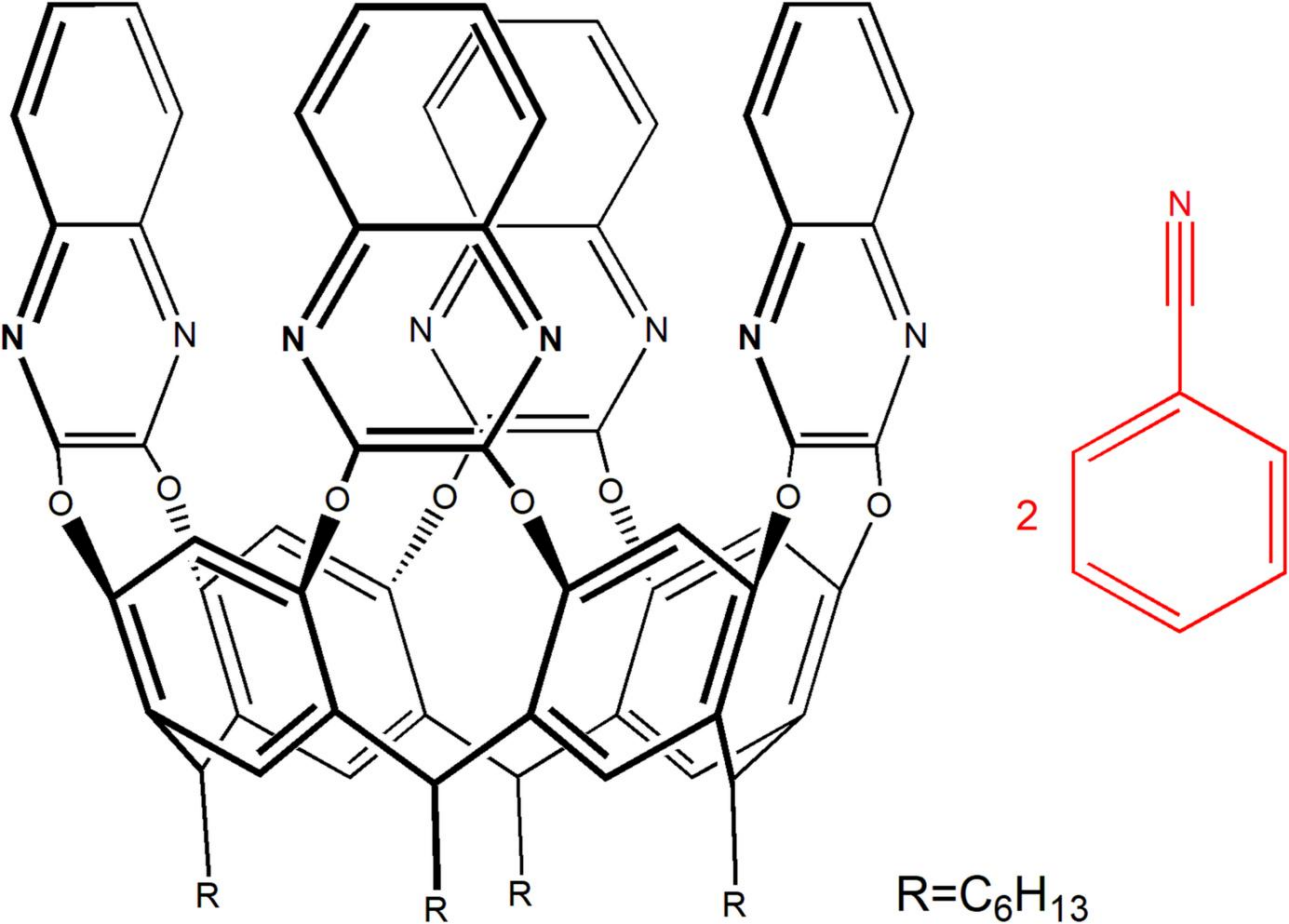


## 2. Structural commentary

**Figure 1:**
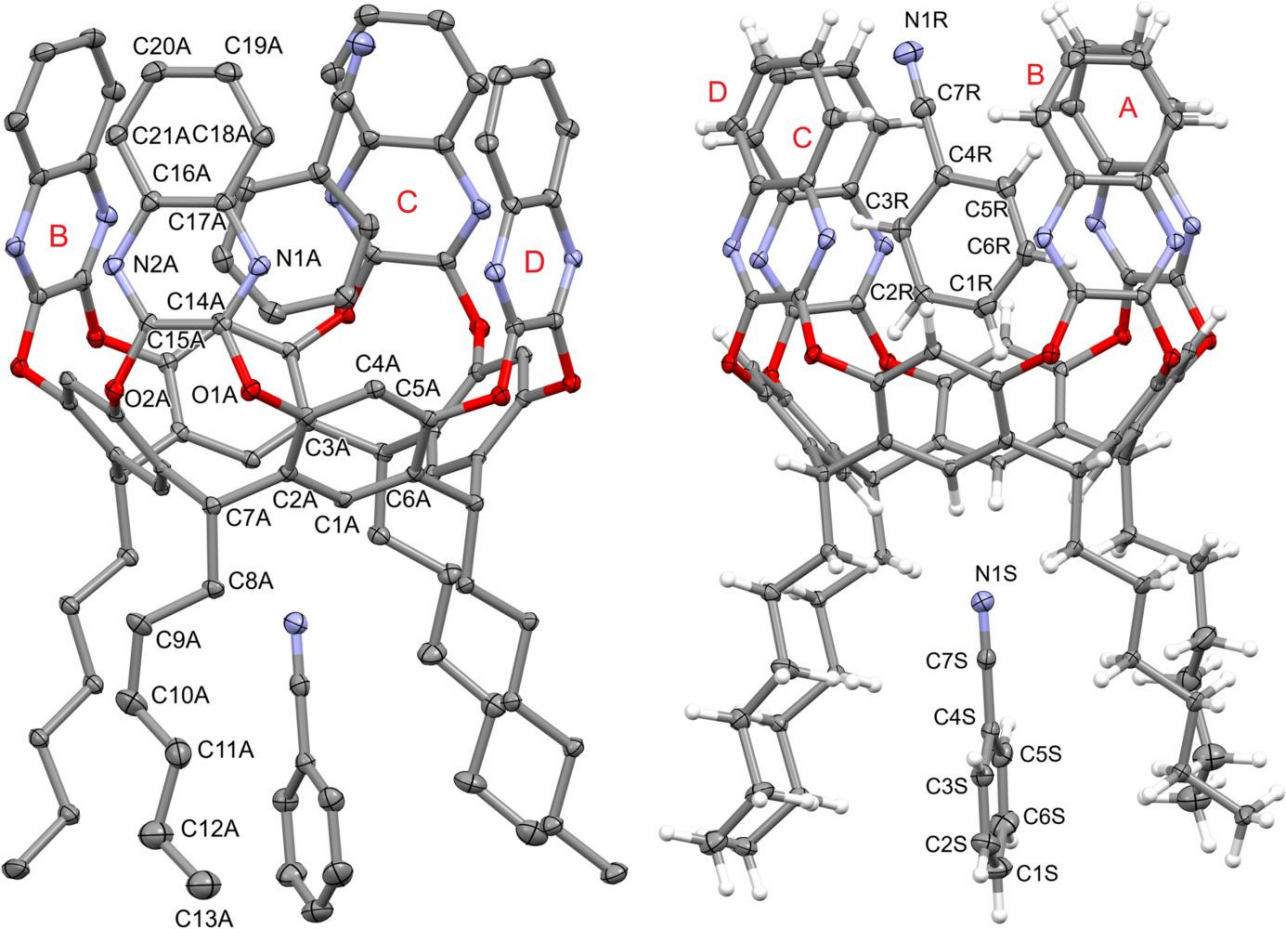
Perspective views of the title complex *A–D* with the labelling scheme for the cavitand (left) and for the benzo­nitrile mol­ecules (right). The ellipsoids are drawn at the 20% probability level. For clarity reasons, only one fourth of the cavitand (*A*) is labelled. The same scheme is applied to the rest of the macrocycle (*B*, *C* and *D*).

The complex (C_84_H_80_N_8_O_8_)·2(C_7_H_5_N) crystallizes in the space group *P* 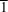 , with two independent mol­ecules (indicated as *A*–*D* and *E*–*H*) in the asymmetric unit, shown in Figs. 1 ▸ and 2 ▸, respectively. One of the benzo­nitrile mol­ecules is engulfed inside the cavity, while the other is located among the alkyl legs at the lower rim. The independent cavitands, both in the *vase* conformation, present minor differences in the cavity dimensions, in the orientation of the benzo­nitrile guest and of the alkyl chains at the lower rim (one of which is disordered over two positions - see the *Refinement* section).

**Figure 3:**
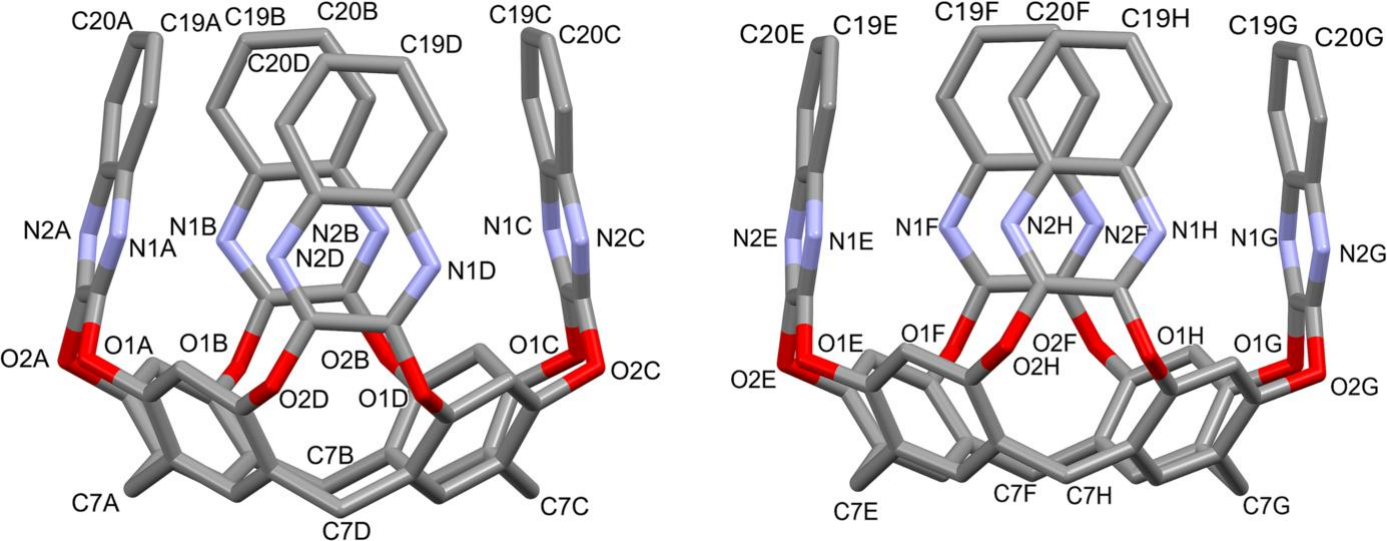
Side view of the cavities of the macrocycles *A*–*D* and *E*–*H* with partial labelling scheme. Alkyl chains and H atoms have been omitted for clarity.

**Figure 5:**
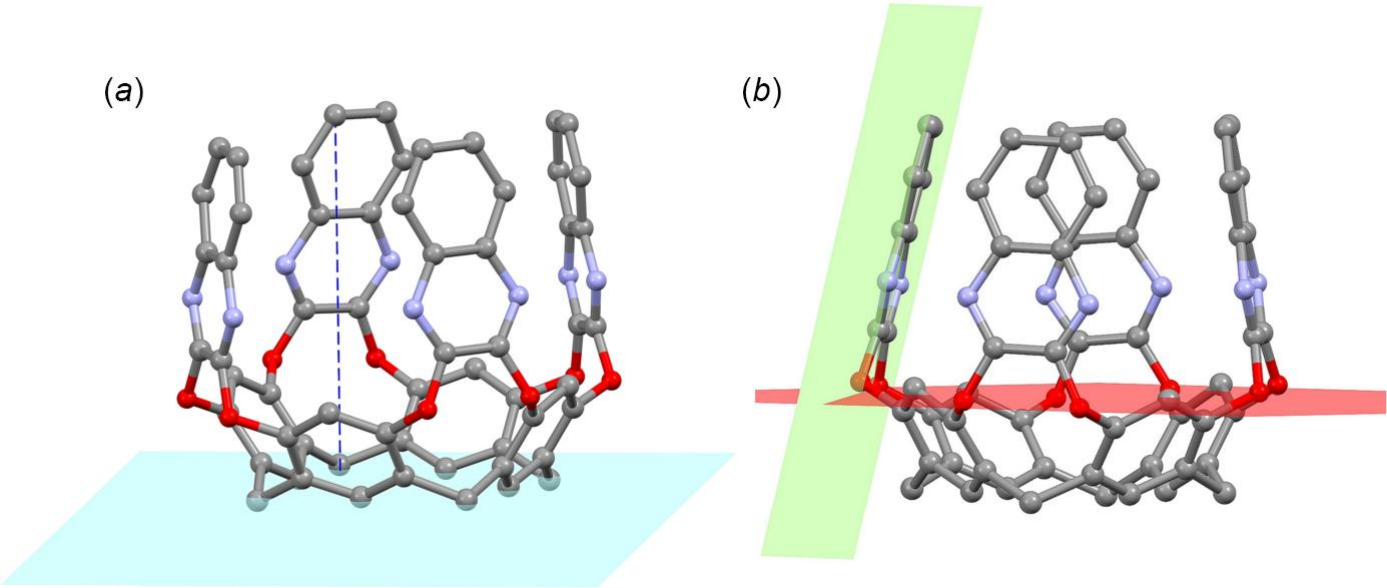
(*a*) View of the mean plane passing through the atoms C7 (light blue) and of the distances from the atom C19*A* of the upper ring to this plane (blue dotted line). The average of the distances from atoms C19–C20 to the plane is reported in the text. (*b*) View of the mean plane passing through the atoms O1/O2 of the cavitand (red) and of the plane passing through the quinoxaline moiety *A* (green). The other planes passing through the moieties *B*, *C*, *D, E*, *F*, *G* and *H* have been calculated in the same way. Alkyl chains and H atoms have been omitted for clarity.

Figs. 3 ▸ and 4 ▸ show two perspective views of the cavities, whose depth has been calculated as the average distance between the mean plane passing through the groups of atoms C7 at the lower rim and the atoms C19–C20 of the upper rim (see Fig. 5 ▸ *a*). The values are of 8.070 (2) and 8.065 (3) Å for *A*–*D* and *E*–*H*, respectively. The mean planes passing through the quinoxaline moieties (atoms C14–C21/N1/N2) are inclined with respect to the plane passing through the O1/O2 atoms, forming angles of 77.12 (3), 84.70 (4), 81.37 (2), 84.57 (2), 84.60 (3), 80.51 (4), 85.37 (3) and 77.33 (3)° for the Qx moieties *A, B, C, D, E, F, G* and *H*, respectively (see Fig. 5 ▸ *b*). Distances and angles are in good agreement with similar compounds reported in the literature, see for instance the acetone clathrate KAJFAC01 (Marsh, 2004 ▸) and other supra­molecular complexes discussed in the *Database survey* section.

## 3. Supra­molecular features

**Figure 6:**
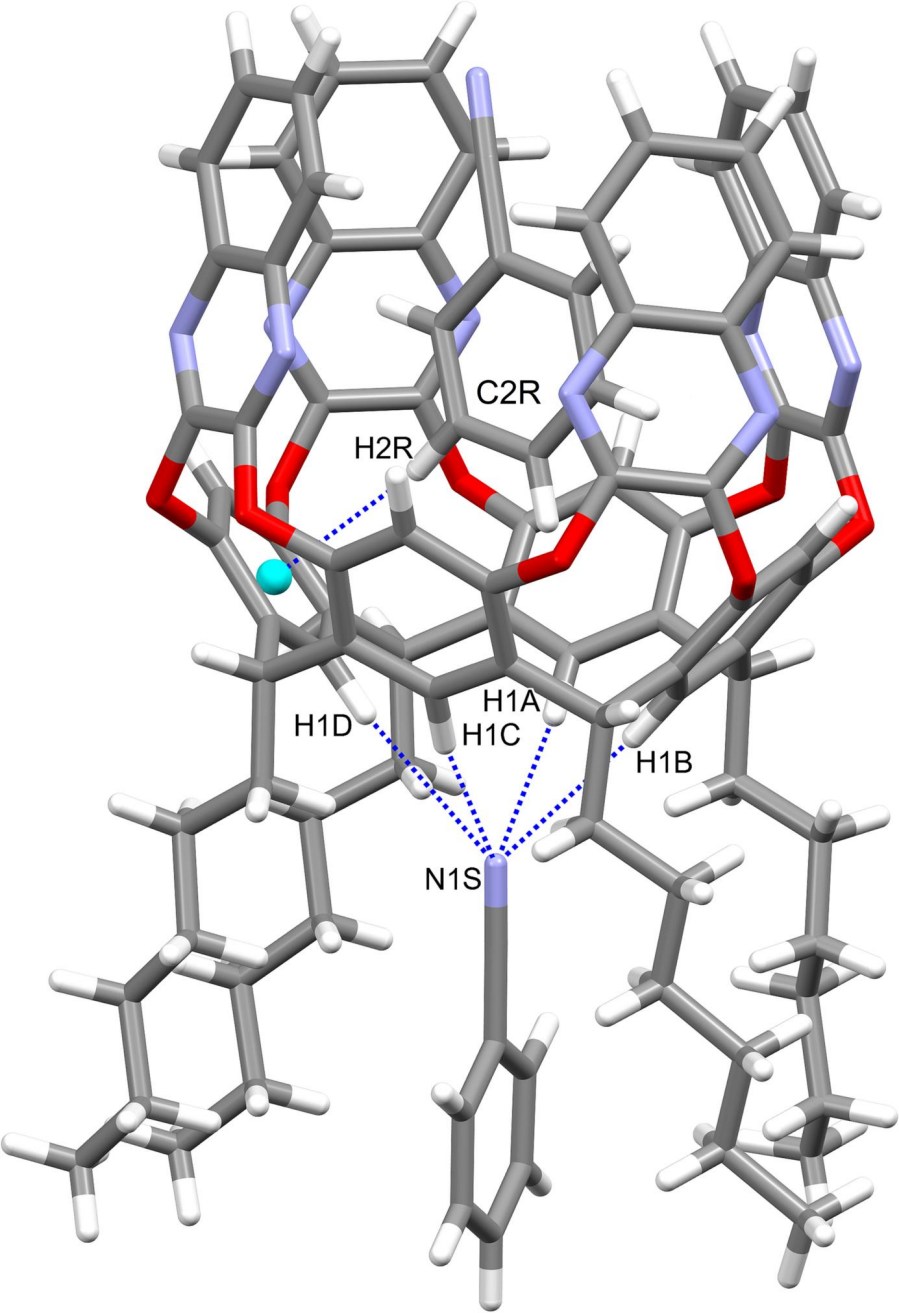
Main supra­molecular inter­actions (blue dotted lines) between the host *A*–*D* and the two benzo­nitrile guest mol­ecules C1*R*–C7*R*/N1*R* and C1*S*–C7*S*/N1*S.* The centroid C*g*1 is shown as a cyan sphere.

**Figure 8:**
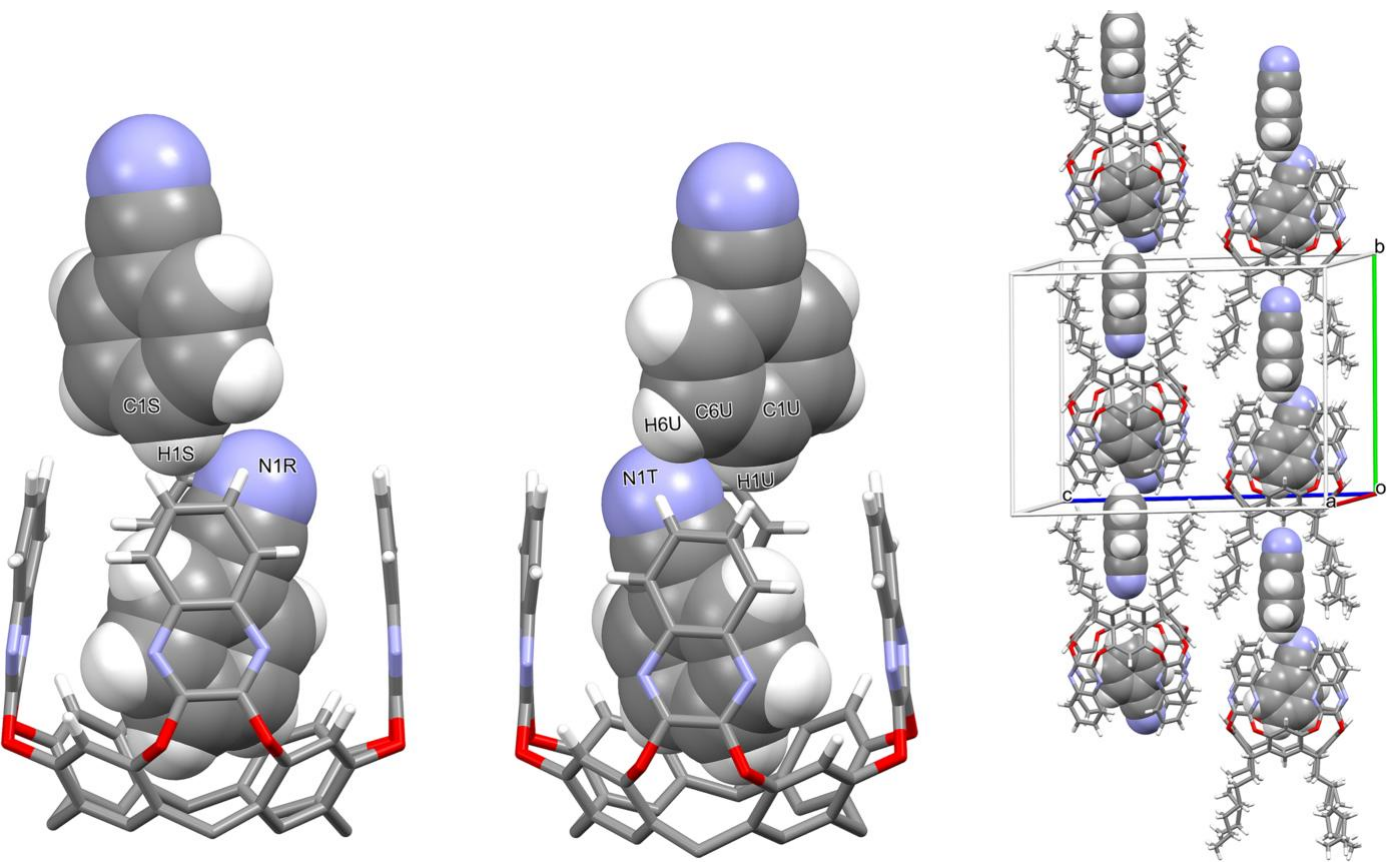
Left and middle: inter­actions between the two different types of benzo­nitrile mol­ecules (inside the cavity and inside the alkyl chains) for cavitands *A*–*D* and *E*–*H*. The symmetry code for the guest C1*R–*C7*R*/N1*R* is *x*, *y* − 1, *z.* Right: supra­molecular chains running along the crystallographic *b* axis.

Each cavitand forms similar supra­molecular complexes with two benzo­nitrile mol­ecules (Figs. 6 ▸ and 7 ▸). In particular, the guests C1*R–*C7*R/*N1*R* and C1*T–*C7*T*/N1*T* are located inside the cavity of macrocycles *A–D* and *E–H*, respectively, with the atoms C1*R* and C1*T* at 0.931 (3) and 0.979 (4) Å from the mean plane passing through the oxygen atoms O1/O2. The aromatic guests are inclined by 85.67 (4)° (benzo­nitrile *R*) and 82.43 (3)° (benzo­nitrile *T*) with respect to the same plane. The host and the guests mainly inter­act through weak C—H⋯π inter­actions with the aromatic walls of the cavitand (Table 1 ▸). The other two benzo­nitrile mol­ecules C1*S*–C7*S*/N1*S* and C1*U*–C7*U*/N1*U* are situated among the alkyl chains of macrocycle *A*–*D* and *E*–*H*, respectively, with atoms N1*S* and N1*U* at 2.595 (2) and 2.626 (3) Å from the mean plane passing through the atoms C7. The most relevant (albeit quite weak) contacts are of the type C—H⋯N: they involve the nitro­gen atoms N1*S* and N1*U* that inter­act with the C—H groups of the alkyl chains and of the aromatic rings of the lower rim, or the C—H groups C1*S*–H2*S*, C1*U*—H1*U* and C6*U*—H6*U* that inter­act with the N atoms N1*R* and N1*T*, respectively, of the benzo­nitrile guests located in the cavity (Table 1 ▸ and Fig. 8 ▸). This gives rise to supra­molecular chains running along the crystallographic *b-*axis direction.

## 4. Database survey

Quinoxaline-based cavitands have been studied for their mol­ecular recognition properties, and a few supra­molecular complexes have been reported in the literature over the past years. A search in the Cambridge Structural Database (Version 2024.1.0, update of November 2023; Groom *et al.*, 2016 ▸) yielded the inclusion compounds of **QxCav** with benzene (BUJNUR; Ballistreri *et al.*, 2016 ▸), 1,3-benzodioxole (LIMFOE; Pinalli *et al.*, 2013 ▸), 5-allyl-1,3-benzodioxole (LIMGAR; Pinalli *et al.*, 2013 ▸), phenyl azide (LUDJEA; Wagner *et al.*, 2009 ▸), fluoro­benzene [YAGVIL (Soncini *et al.*, 1992 ▸) and YAGVIL01 (Marsh, 2004 ▸)] and acetro­nitrile (UNIDUQ; Azov *et al.*, 2003 ▸).

BUJNUR is a fullerene clathrate, with one mol­ecule of benzene inside the cavity and three other mol­ecules outside it, while the fullerene mol­ecule inter­acts with the aliphatic chains of the host. The benzene mol­ecule inside the cavity is at a distance of *ca* 1.2 Å from the mean plane passing through the oxygen atoms and forms two sets of weak inter­actions with the N atoms of the quinoxaline walls [C⋯N distances spanning from 3.580 (4) to 3.752 (7) Å].

In the case of LIMFOE, the benzodioxole enters the cavity with the aromatic ring, fitting the space formed by the four quinoxaline walls and inter­acting through weak C—H⋯π contacts with the scaffold of the cavitand, in a manner similar to that of the title compound [C—H⋯centroid: 2.445 (3) Å and 160.3 (2)°]. Differently, in the structure of LIMFOE, the dioxolane ring of the guest points inside the cavity, forming two C—H⋯π inter­actions with the aromatic rings of the resorcinarene scaffold [C—H⋯centroid: 2.705 (4), 2.793 (2) Å, 165.3 (6) and 156.0 (4)°, respectively]. The different behaviour is probably due to the steric hindrance caused by the aliphatic chain of 5-allyl-1,3-benzodioxole, which cannot be conveniently accommodated inside the cavity.

The guest phenyl azide (LUDJEA; Wagner *et al.*, 2009 ▸) also enters the cavity of the macrocycle with its phenyl ring positioned between two of the quinoxaline walls. Three of the walls are slightly tilted towards the inside of the cavity to engulf the guest completely and maximize van der Waals inter­actions and weak C—H⋯π contacts. The fourth wall, on the contrary, points towards the outside of the cavity due to the steric hindrance caused by the azide group.

In the case of fluoro­benzene (YAGVIL), as for the title compound, the stoichiometry of the supra­molecular complex is 2:1; one guest is located inside the cavity, while the other one is among the alkyl chains of the lower rim. The C–F axis of the guest inside the cavity is inclined by 19.2 (2)° with respect to the normal to the mean plane passing through the oxygen atoms, with the F atom pointing toward the portal of the vase. The inter­actions are mainly of van der Waals type, with the presence of the usual weak C—H⋯π inter­actions between the guest and the aromatic ring of the host. The orientation of fluoro­benzene is slightly different since the C–F axis of the guest lies on the twofold axis passing through the centre of the cavitand.

## 5. Synthesis and crystallization

The synthesis of **QxCav** was carried out according to the literature (Soncini *et al.*, 1992 ▸). All commercial reagents were ACS grade and used as received. Solvents were dried and distilled using standard procedures. Prismatic, colourless single crystals of the title compound suitable for X-ray analysis were obtained by slow evaporation of a solution of **QxCav** in benzo­nitrile.

## 6. Refinement

Crystal data, data collection and structure refinement details are summarized in Table 2 ▸.

One of the alkyl chains in cavitand *E*–*H* (atoms C10–C13) was found to be disordered over two positions with occupancies set to 0.7 for atoms C10*H*–C13*H* and 0.3 for atoms C10*I*–C13*I*. Distances were restrained to obtain reasonable values in agreement with *sp* ^3^ hybridization. Restraints were applied to the ADP’s of the atoms belonging to the disordered alkyl chain using the commands SIMU and DELU.

The highest peak (1.27 e Å^−3^ at 0.9003 0.1896 0.0288) was found at 1.16 Å from the hydrogen atom H10*A*, bonded to the carbon atom C11*A* of an alkyl chain. This could be a sign of mild disorder, but attempts to model the disorder lead to unsatisfactory results.

The carbon-bound H atoms were placed in calculated positions and refined isotropically using the riding model, with C—H distances ranging from 0.95 to 0.99 Å and *U*_iso_(H) set to 1.2–1.5*U*_eq_(C).

## Supplementary Material

Crystal structure: contains datablock(s) I. DOI: 10.1107/S205698902400481X/jw2007sup1.cif

Structure factors: contains datablock(s) I. DOI: 10.1107/S205698902400481X/jw2007Isup3.hkl

CCDC reference: 2357457

Additional supporting information:  crystallographic information; 3D view; checkCIF report

## Figures and Tables

**Figure 2:**
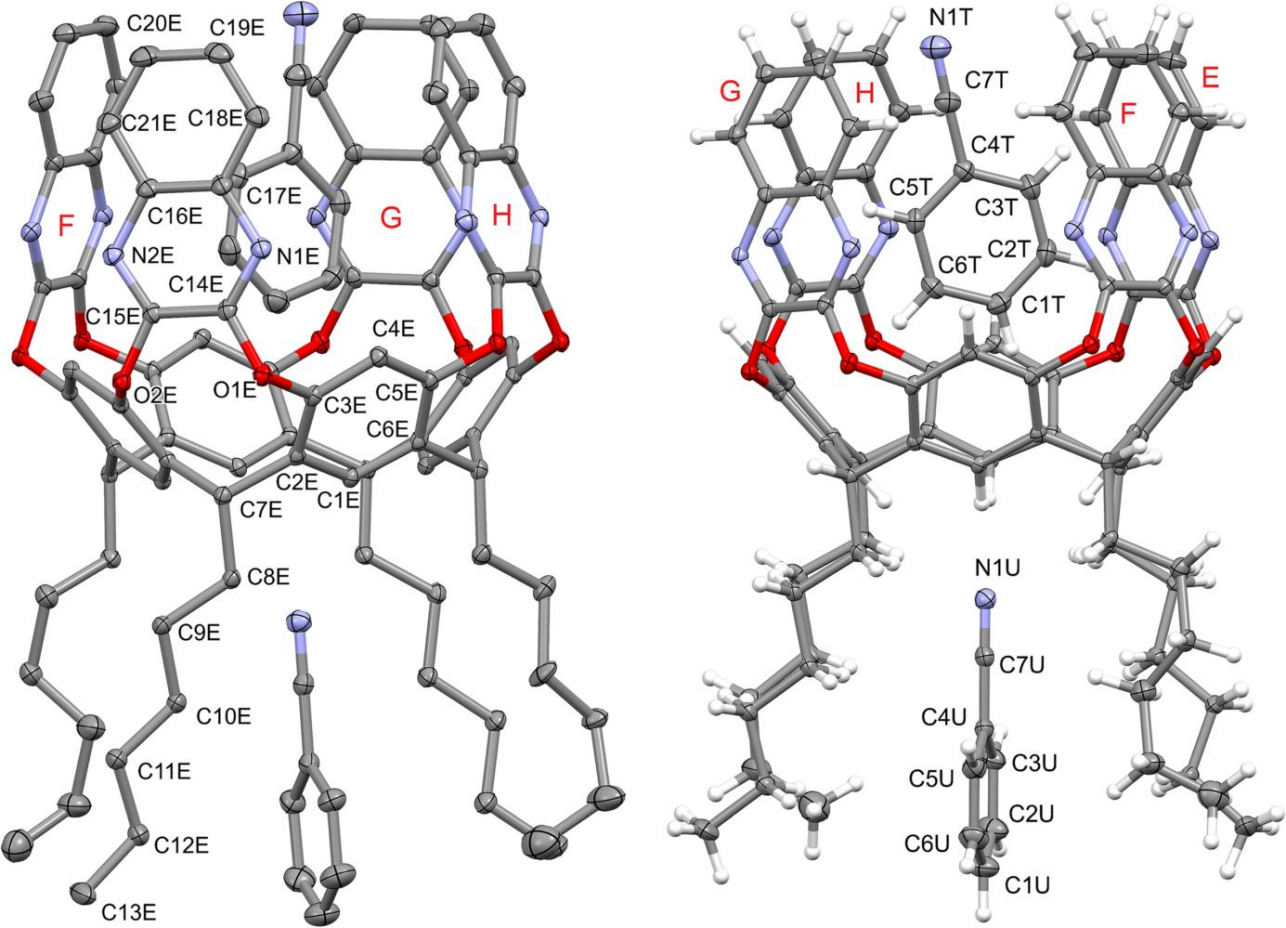
Perspective views of the title complex *E*–*H* with the labelling scheme for the cavitand (left) and for the benzo­nitrile mol­ecules (right; the symmetry code for the guest C1*U*–C7*U*/N1*U* is *x*, *y* − 1, *z*). The ellipsoids are drawn at the 20% probability level. For clarity reasons, only one fourth of the cavitand (*E*) is labelled. The same scheme is applied to the rest of the macrocycle (*F*, *G* and *H*). Only one orientation of the disordered alkyl chain (*H*) is shown for clarity.

**Figure 4:**
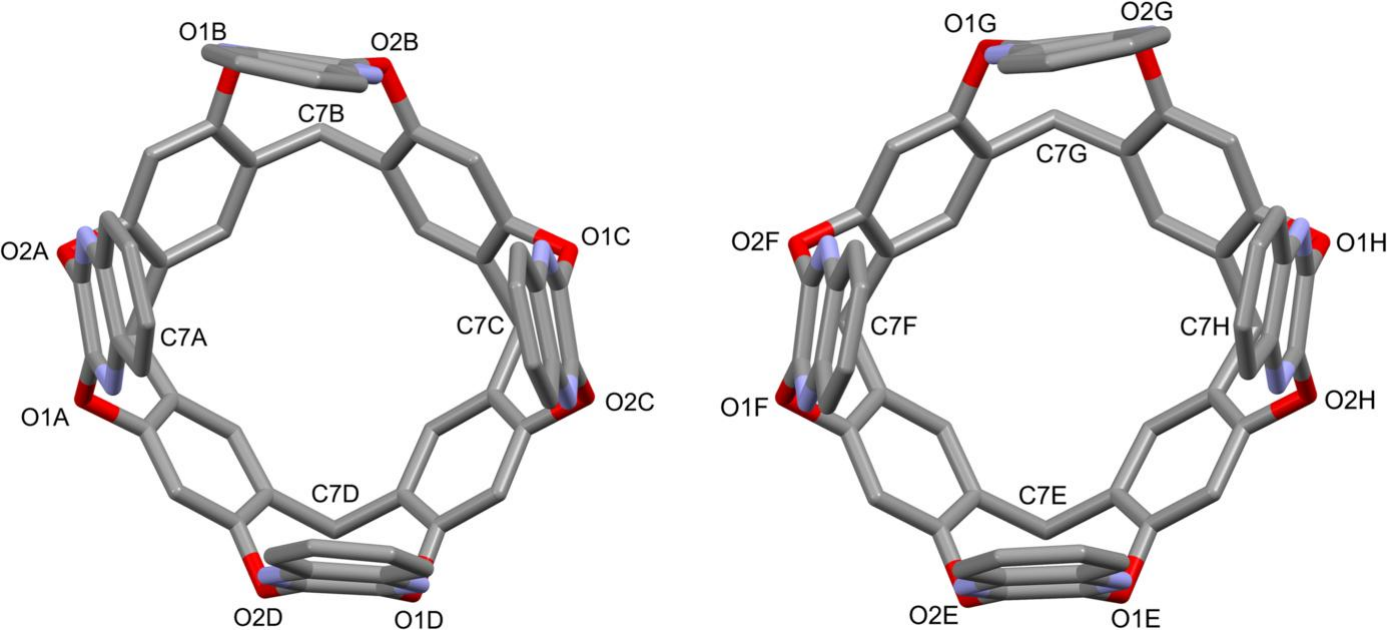
Top view of the cavities of the macrocycles *A*–*D* and *E*–*H* with partial labelling scheme. Alkyl chains and H atoms have been omitted for clarity.

**Figure 7:**
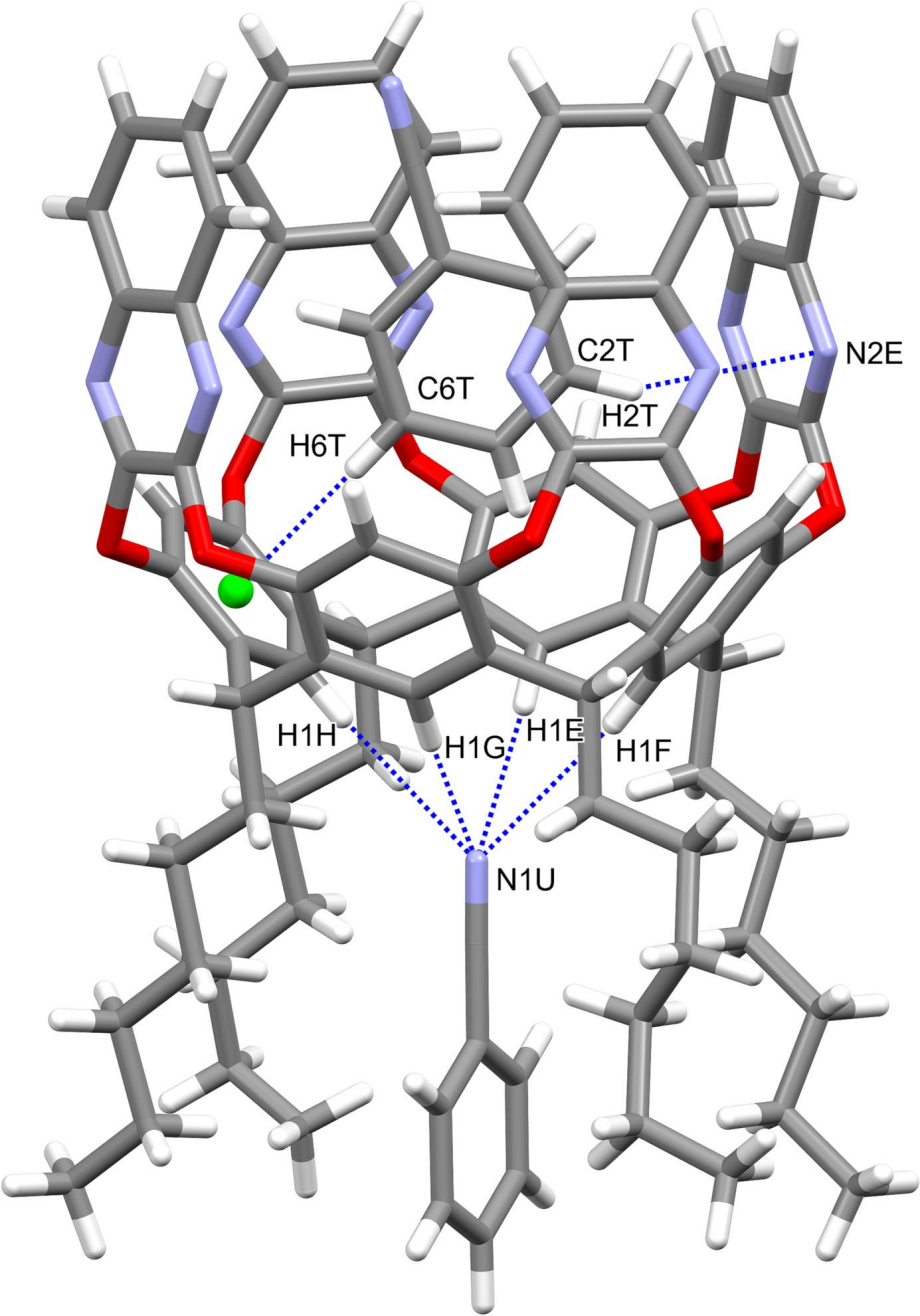
Main supra­molecular inter­actions (blue dotted lines) between the host *E*–*H* and the two benzo­nitrile guest mol­ecules C1*T*–C7*T*/N1*T* and C1*U–*C7*U*/N1*U.* The symmetry code for the guest C1*U*–C7*U*/N1*U* is *x*, *y* − 1, *z.* The centroid C*g*2 is shown as a green sphere.

**Table 1:** Hydrogen-bond geometry (Å, °) C*g*1 is the centroid of the ring C1*D*–C6*D* and C*g*2 is the centroid of the ring C1*H*–C6*H*.

*D*—H⋯*A*	*D*—H	H⋯*A*	*D*⋯*A*	*D*—H⋯*A*
C2*R*—H2*R*⋯C*g*1	0.95	2.60	3.532 (1)	166
C6*T*—H6*T*⋯C*g*2	0.95	2.63	3.566 (2)	169
C2*T*—H2*T*⋯N2*E*	0.95	2.88	3.734 (2)	150
C1*A*—H1*A*⋯N1*S*	0.95	2.76	3.693 (2)	169
C1*B*—H1*B*⋯N1*S*	0.95	2.79	3.742 (3)	176
C1*C*—H1*C*⋯N1*S*	0.95	2.79	3.714 (2)	166
C1*D*—H1*D*⋯N1*S*	0.95	2.83	3.775 (1)	173
C1*E*—H1*E*⋯N1*U* ^i^	0.95	2.85	3.784 (1)	169
C1*F*—H1*F*⋯N1*U* ^i^	0.95	2.85	3.798 (2)	174
C1*G*—H1*G*⋯N1*U* ^i^	0.95	2.73	3.673 (3)	171
C1*H*—H1*H*⋯N1*U* ^i^	0.95	2.80	3.752 (2)	175
C1*S*—H1*S*⋯N1*R* ^ii^	0.95	2.57	3.304 (1)	134
C1*U*—H1*U*⋯N1*T*	0.95	2.74	3.313 (2)	120
C6*U*—H6*U*⋯N1*T*	0.95	2.78	3.333 (2)	118

Symmetry codes: (i) 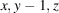 ; (ii) 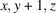 .

**Table 2:** Experimental details

Crystal data	
Chemical formula	C_84_H_80_N_8_O_8_·2C_7_H_5_N
*M*_r_	1535.79
Crystal system, space group	Triclinic, *P* 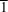
Temperature (K)	150
*a*, *b*, *c* (Å)	18.6922 (5), 18.7278 (5), 24.4009 (6)
α, β, γ (°)	89.992 (2), 70.083 (1), 85.978 (2)
*V* (Å^3^)	8008.6 (4)
*Z*	4
Radiation type	Cu *K*α
μ (mm^−1^)	0.65
Crystal size (mm)	0.17 × 0.14 × 0.09

Data collection	
Diffractometer	Bruker D8 Venture PhotonII
Absorption correction	Multi-scan (*SADABS*; Krause *et al.*, 2015 ▸)
*T*_min_, *T*_max_	0.651, 0.754
No. of measured, independent and observed [*I* > 2σ(*I*)] reflections	105559, 32799, 23928
*R*_int_	0.057
(sin θ/λ)_max_ (Å^−1^)	0.628

Refinement	
*R*[*F* ^2^ > 2σ(*F* ^2^)], *wR*(*F* ^2^), *S*	0.064, 0.190, 1.02
No. of reflections	32799
No. of parameters	2120
No. of restraints	138
H-atom treatment	H-atom parameters constrained
Δρ_max_, Δρ_min_ (e Å^−3^)	1.27, −0.74

Computer programs: *APEX3* and *SAINT* (Bruker, 2016 ▸), *SHELXT2018/2* (Sheldrick, 2015*a* ▸), *SHELXL2019/3* (Sheldrick, 2015*b* ▸), *Mercury* (Macrae *et al.*, 2020 ▸), *WinGX* (Farrugia, 2012 ▸), *publCIF* (Westrip, 2010 ▸) and *enCIFer* (Allen *et al.*, 2004 ▸).

## supplementary crystallographic information

### Crystal data

**Table:**

C_84_H_80_N_8_O_8_·2C_7_H_5_N	*Z* = 4
*M**_r_* = 1535.79	*F*(000) = 3248
Triclinic, *P*1	*D*_x_ = 1.274 Mg m^−^^3^
*a* = 18.6922 (5) Å	Cu *K*α radiation, λ = 1.54178 Å
*b* = 18.7278 (5) Å	Cell parameters from 2780 reflections
*c* = 24.4009 (6) Å	θ = 1.9–75.6°
α = 89.992 (2)°	µ = 0.65 mm^−^^1^
β = 70.083 (1)°	*T* = 150 K
γ = 85.978 (2)°	Prismatic, colourless
*V* = 8008.6 (4) Å^3^	0.17 × 0.14 × 0.09 mm

### Data collection

**Table:**

Bruker D8 Venture PhotonII diffractometer	32799 independent reflections
Radiation source: fine-focus sealed tube	23928 reflections with *I* > 2σ(*I*)
Graphite monochromator	*R*_int_ = 0.057
phi & ω scan	θ_max_ = 75.6°, θ_min_ = 1.9°
Absorption correction: multi-scan (SADABS; Krause *et al.*, 2015)	*h* = −23→23
*T*_min_ = 0.651, *T*_max_ = 0.754	*k* = −23→20
105559 measured reflections	*l* = −30→30

### Refinement

**Table:**

Refinement on *F*^2^	138 restraints
Least-squares matrix: full	Hydrogen site location: inferred from neighbouring sites
*R*[*F*^2^ > 2σ(*F*^2^)] = 0.064	H-atom parameters constrained
*wR*(*F*^2^) = 0.190	*w* = 1/[σ^2^(*F*_o_^2^) + (0.0909*P*)^2^ + 5.2712*P*] where *P* = (*F*_o_^2^ + 2*F*_c_^2^)/3
*S* = 1.02	(Δ/σ)_max_ < 0.001
32799 reflections	Δρ_max_ = 1.27 e Å^−^^3^
2120 parameters	Δρ_min_ = −0.74 e Å^−^^3^

### Special details

**Table:**

Geometry. All esds (except the esd in the dihedral angle between two l.s. planes) are estimated using the full covariance matrix. The cell esds are taken into account individually in the estimation of esds in distances, angles and torsion angles; correlations between esds in cell parameters are only used when they are defined by crystal symmetry. An approximate (isotropic) treatment of cell esds is used for estimating esds involving l.s. planes.

### Fractional atomic coordinates and isotropic or equivalent isotropic displacement parameters (Å^2^)

**Table:**

	*x*	*y*	*z*	*U*_iso_*/*U*_eq_	Occ. (<1)
N1A	0.01973 (11)	0.32461 (12)	0.86763 (9)	0.0373 (4)	
N2A	0.10037 (12)	0.31295 (12)	0.94684 (9)	0.0369 (4)	
N1B	0.33309 (12)	0.29008 (11)	0.92545 (8)	0.0366 (4)	
N2B	0.43666 (11)	0.26871 (12)	0.81017 (9)	0.0379 (5)	
N1C	0.45246 (11)	0.26805 (12)	0.62597 (9)	0.0371 (5)	
N2C	0.37174 (11)	0.29061 (12)	0.54755 (8)	0.0364 (4)	
N1D	0.13381 (11)	0.34407 (11)	0.57744 (8)	0.0337 (4)	
N2D	0.01625 (12)	0.34954 (12)	0.68750 (9)	0.0377 (5)	
N1E	0.47519 (12)	0.17761 (12)	0.30519 (9)	0.0381 (5)	
N2E	0.35445 (12)	0.18310 (12)	0.41338 (9)	0.0373 (4)	
N1F	0.11625 (11)	0.20408 (11)	0.44473 (8)	0.0356 (4)	
N2F	0.03169 (11)	0.20625 (12)	0.36850 (9)	0.0370 (4)	
N1G	0.03976 (11)	0.17993 (12)	0.18571 (8)	0.0376 (5)	
N2G	0.14979 (11)	0.16189 (11)	0.07271 (8)	0.0337 (4)	
N1H	0.38721 (12)	0.16992 (12)	0.04984 (8)	0.0370 (4)	
N2H	0.46979 (12)	0.17871 (12)	0.12709 (9)	0.0388 (5)	
O1A	0.00098 (9)	0.44601 (9)	0.88824 (7)	0.0335 (3)	
O2A	0.08588 (9)	0.43600 (9)	0.95831 (6)	0.0338 (3)	
O1B	0.35557 (9)	0.40976 (9)	0.91870 (7)	0.0337 (3)	
O2B	0.46379 (9)	0.38701 (9)	0.81292 (7)	0.0352 (4)	
O1C	0.48625 (9)	0.38453 (9)	0.61051 (7)	0.0345 (4)	
O2C	0.40256 (9)	0.40847 (9)	0.54166 (6)	0.0334 (3)	
O1D	0.13511 (9)	0.46676 (9)	0.58421 (6)	0.0319 (3)	
O2D	0.01966 (9)	0.47234 (9)	0.68771 (7)	0.0341 (3)	
O1E	0.48563 (9)	0.05487 (9)	0.31228 (7)	0.0341 (3)	
O2E	0.36934 (9)	0.06004 (9)	0.41532 (6)	0.0323 (3)	
O1F	0.09842 (9)	0.08375 (9)	0.45783 (6)	0.0317 (3)	
O2F	0.01090 (9)	0.08749 (9)	0.38962 (6)	0.0333 (3)	
O1G	0.03305 (9)	0.05764 (9)	0.18821 (7)	0.0345 (4)	
O2G	0.14735 (9)	0.03970 (9)	0.08363 (6)	0.0320 (3)	
O1H	0.41613 (9)	0.04782 (9)	0.04268 (6)	0.0341 (4)	
O2H	0.50288 (9)	0.05743 (9)	0.11104 (7)	0.0349 (4)	
C1A	0.11209 (12)	0.56298 (12)	0.77600 (9)	0.0286 (4)	
H1A	0.146513	0.599229	0.771971	0.034*	
C2A	0.08176 (12)	0.53194 (12)	0.83023 (9)	0.0288 (4)	
C3A	0.03400 (12)	0.47712 (12)	0.83380 (9)	0.0290 (4)	
C4A	0.01352 (12)	0.45546 (13)	0.78728 (9)	0.0305 (5)	
H4A	−0.019900	0.418364	0.791137	0.037*	
C5A	0.04355 (12)	0.48988 (13)	0.73467 (9)	0.0304 (5)	
C6A	0.09400 (12)	0.54303 (12)	0.72719 (9)	0.0284 (4)	
C7A	0.09606 (12)	0.55876 (12)	0.88458 (9)	0.0302 (5)	
H7A	0.050864	0.547921	0.918968	0.036*	
C8A	0.10142 (14)	0.64013 (13)	0.88531 (10)	0.0366 (5)	
H8A1	0.147926	0.652304	0.853417	0.044*	
H8A2	0.056830	0.663885	0.877726	0.044*	
C9A	0.10401 (17)	0.66927 (16)	0.94312 (12)	0.0511 (7)	
H9A1	0.053928	0.663371	0.973523	0.061*	
H9A2	0.142660	0.638993	0.953716	0.061*	
C10A	0.1218 (3)	0.7463 (2)	0.94541 (19)	0.0819 (12)	
H10A	0.124029	0.756816	0.984540	0.098*	
H10B	0.173188	0.751874	0.916677	0.098*	
C11A	0.0687 (2)	0.7992 (2)	0.93400 (17)	0.0741 (10)	
H11A	0.015931	0.788968	0.957582	0.089*	
H11B	0.073688	0.795814	0.892352	0.089*	
C12A	0.0835 (3)	0.8781 (2)	0.9491 (2)	0.0891 (9)	
H12A	0.077387	0.881824	0.990977	0.107*	
H12B	0.136526	0.888048	0.926132	0.107*	
C13A	0.0310 (3)	0.9308 (2)	0.9365 (2)	0.0891 (9)	
H13A	0.038379	0.928452	0.894783	0.134*	
H13B	0.040764	0.978722	0.947070	0.134*	
H13C	−0.021544	0.920860	0.959076	0.134*	
C14A	0.03119 (12)	0.37926 (13)	0.89471 (9)	0.0325 (5)	
C15A	0.07362 (13)	0.37376 (14)	0.93398 (9)	0.0337 (5)	
C16A	0.08748 (14)	0.25322 (14)	0.92016 (11)	0.0388 (5)	
C17A	0.04907 (14)	0.25912 (14)	0.87915 (11)	0.0379 (5)	
C18A	0.04098 (17)	0.19712 (16)	0.85006 (13)	0.0484 (6)	
H18A	0.016045	0.200673	0.822026	0.058*	
C19A	0.06875 (18)	0.13180 (17)	0.86189 (15)	0.0551 (7)	
H19A	0.063055	0.090108	0.842039	0.066*	
C20A	0.10571 (18)	0.12589 (17)	0.90325 (15)	0.0548 (7)	
H20A	0.124559	0.080105	0.911285	0.066*	
C21A	0.11485 (17)	0.18509 (15)	0.93202 (13)	0.0474 (6)	
H21A	0.139702	0.180364	0.960057	0.057*	
C1B	0.23969 (12)	0.53729 (12)	0.86306 (9)	0.0279 (4)	
H1B	0.246255	0.576118	0.837168	0.033*	
C2B	0.30450 (13)	0.50271 (12)	0.86999 (9)	0.0290 (4)	
C3B	0.29276 (13)	0.44648 (12)	0.90834 (9)	0.0295 (5)	
C4B	0.22095 (13)	0.42457 (12)	0.93779 (9)	0.0306 (5)	
H4B	0.214518	0.385889	0.963811	0.037*	
C5B	0.15847 (12)	0.45988 (12)	0.92877 (9)	0.0288 (4)	
C6B	0.16574 (13)	0.51797 (12)	0.89209 (9)	0.0284 (4)	
C7B	0.38444 (13)	0.52427 (13)	0.83519 (9)	0.0312 (5)	
H7B	0.419651	0.505949	0.855868	0.037*	
C8B	0.38895 (14)	0.60540 (13)	0.83051 (10)	0.0357 (5)	
H8B1	0.442184	0.614983	0.807673	0.043*	
H8B2	0.356490	0.623538	0.808089	0.043*	
C9B	0.36497 (15)	0.64836 (14)	0.88789 (10)	0.0381 (5)	
H9B1	0.315722	0.633032	0.914428	0.046*	
H9B2	0.403671	0.639124	0.906927	0.046*	
C10B	0.35690 (15)	0.72799 (14)	0.87680 (10)	0.0383 (5)	
H10C	0.318157	0.736131	0.857678	0.046*	
H10D	0.406093	0.741929	0.849143	0.046*	
C11B	0.33429 (16)	0.77695 (14)	0.93070 (10)	0.0399 (6)	
H11C	0.287618	0.760690	0.960144	0.048*	
H11D	0.375511	0.773243	0.947682	0.048*	
C12B	0.31972 (17)	0.85416 (15)	0.91742 (11)	0.0445 (6)	
H12C	0.279085	0.857641	0.899852	0.053*	
H12D	0.366656	0.870496	0.888352	0.053*	
C13B	0.2960 (3)	0.90339 (18)	0.97096 (14)	0.0878 (15)	
H13D	0.249375	0.887761	0.999856	0.132*	
H13E	0.286575	0.952510	0.959885	0.132*	
H13F	0.336881	0.901689	0.987725	0.132*	
C14B	0.36946 (13)	0.34139 (13)	0.89522 (10)	0.0334 (5)	
C15B	0.42359 (13)	0.32961 (14)	0.83769 (10)	0.0337 (5)	
C16B	0.39646 (14)	0.21372 (14)	0.83978 (10)	0.0371 (5)	
C17B	0.34678 (14)	0.22397 (14)	0.89810 (10)	0.0378 (5)	
C18B	0.30878 (17)	0.16579 (15)	0.92839 (12)	0.0484 (7)	
H18B	0.276695	0.171761	0.968008	0.058*	
C19B	0.31840 (19)	0.10066 (16)	0.90033 (14)	0.0547 (7)	
H19B	0.292422	0.061556	0.920586	0.066*	
C20B	0.36631 (18)	0.09103 (16)	0.84188 (13)	0.0508 (7)	
H20B	0.371890	0.045641	0.822911	0.061*	
C21B	0.40481 (15)	0.14605 (15)	0.81215 (12)	0.0443 (6)	
H21B	0.437327	0.138811	0.772743	0.053*	
C1C	0.39979 (12)	0.51949 (13)	0.72657 (9)	0.0302 (5)	
H1C	0.374357	0.566019	0.731655	0.036*	
C2C	0.42397 (12)	0.48674 (13)	0.67108 (9)	0.0301 (5)	
C3C	0.45892 (12)	0.41777 (13)	0.66618 (9)	0.0317 (5)	
C4C	0.47094 (12)	0.38297 (13)	0.71257 (10)	0.0322 (5)	
H4C	0.494853	0.335816	0.707837	0.039*	
C5C	0.44716 (12)	0.41871 (13)	0.76614 (10)	0.0319 (5)	
C6C	0.41110 (12)	0.48731 (13)	0.77475 (9)	0.0306 (5)	
C7C	0.41435 (12)	0.52395 (13)	0.61789 (9)	0.0308 (5)	
H7C	0.456897	0.503817	0.582861	0.037*	
C8C	0.42341 (14)	0.60478 (14)	0.62102 (10)	0.0368 (5)	
H8C1	0.383934	0.624894	0.657109	0.044*	
H8C2	0.473681	0.610892	0.624869	0.044*	
C9C	0.41781 (15)	0.64919 (15)	0.57039 (11)	0.0412 (6)	
H9C1	0.365639	0.649651	0.568633	0.049*	
H9C2	0.454249	0.628526	0.533169	0.049*	
C10C	0.4368 (2)	0.72607 (18)	0.58008 (16)	0.0599 (8)	
H10E	0.407135	0.741185	0.620863	0.072*	
H10F	0.491499	0.724666	0.575806	0.072*	
C11C	0.42204 (18)	0.78211 (17)	0.54110 (13)	0.0527 (7)	
H11E	0.367682	0.783588	0.544375	0.063*	
H11F	0.453235	0.768865	0.500217	0.063*	
C12C	0.4404 (2)	0.85660 (18)	0.55547 (18)	0.0679 (9)	
H12E	0.407798	0.870685	0.595862	0.081*	
H12F	0.494222	0.854831	0.553539	0.081*	
C13C	0.4281 (2)	0.9112 (2)	0.51532 (17)	0.0722 (10)	
H13G	0.458243	0.896383	0.475073	0.108*	
H13H	0.444052	0.957146	0.524520	0.108*	
H13I	0.373878	0.916420	0.519719	0.108*	
C14C	0.44760 (12)	0.32867 (13)	0.60181 (9)	0.0320 (5)	
C15C	0.40553 (13)	0.34069 (13)	0.56310 (9)	0.0327 (5)	
C16C	0.37706 (14)	0.22449 (14)	0.57123 (10)	0.0360 (5)	
C17C	0.41596 (14)	0.21378 (14)	0.61151 (10)	0.0370 (5)	
C18C	0.41874 (17)	0.14589 (16)	0.63597 (12)	0.0471 (6)	
H18C	0.444077	0.138457	0.663501	0.057*	
C19C	0.38516 (18)	0.09086 (17)	0.62020 (14)	0.0530 (7)	
H19C	0.387045	0.045180	0.636798	0.064*	
C20C	0.34765 (18)	0.10175 (16)	0.57931 (13)	0.0515 (7)	
H20C	0.324876	0.062991	0.568251	0.062*	
C21C	0.34354 (15)	0.16681 (15)	0.55542 (12)	0.0441 (6)	
H21C	0.317921	0.173231	0.527975	0.053*	
C1D	0.27169 (12)	0.54650 (12)	0.63966 (9)	0.0285 (4)	
H1D	0.272478	0.583656	0.665909	0.034*	
C2D	0.20220 (12)	0.53448 (12)	0.63287 (9)	0.0278 (4)	
C3D	0.20300 (12)	0.47935 (12)	0.59418 (9)	0.0282 (4)	
C4D	0.26901 (13)	0.43753 (12)	0.56461 (9)	0.0300 (5)	
H4D	0.268147	0.399809	0.538862	0.036*	
C5D	0.33617 (12)	0.45138 (12)	0.57305 (9)	0.0294 (4)	
C6D	0.34000 (12)	0.50673 (12)	0.60986 (9)	0.0288 (4)	
C7D	0.12823 (12)	0.57763 (12)	0.66803 (9)	0.0291 (4)	
H7D	0.091468	0.574282	0.646616	0.035*	
C8D	0.13895 (13)	0.65726 (12)	0.67484 (9)	0.0309 (5)	
H8D1	0.090834	0.680220	0.702417	0.037*	
H8D2	0.178987	0.661094	0.692553	0.037*	
C9D	0.16091 (14)	0.69931 (13)	0.61840 (9)	0.0335 (5)	
H9D1	0.208676	0.676840	0.590002	0.040*	
H9D2	0.120205	0.698302	0.601048	0.040*	
C10D	0.17211 (15)	0.77639 (14)	0.63122 (10)	0.0392 (5)	
H10G	0.127409	0.795081	0.664624	0.047*	
H10H	0.217518	0.776661	0.643359	0.047*	
C11D	0.18227 (15)	0.82696 (14)	0.58117 (10)	0.0376 (5)	
H11G	0.136189	0.828629	0.569765	0.045*	
H11H	0.226221	0.808207	0.547232	0.045*	
C12D	0.19553 (17)	0.90217 (15)	0.59639 (12)	0.0448 (6)	
H12G	0.153717	0.919233	0.632334	0.054*	
H12H	0.243872	0.900894	0.604703	0.054*	
C13D	0.1995 (2)	0.95472 (16)	0.54867 (14)	0.0559 (7)	
H13J	0.238354	0.936600	0.512199	0.084*	
H13K	0.212812	1.001045	0.559505	0.084*	
H13L	0.149743	0.960604	0.543341	0.084*	
C14D	0.10635 (13)	0.40325 (13)	0.60646 (9)	0.0312 (5)	
C15D	0.04641 (13)	0.40614 (13)	0.66218 (10)	0.0330 (5)	
C16D	0.04642 (14)	0.28548 (14)	0.65878 (11)	0.0368 (5)	
C17D	0.10425 (13)	0.28237 (14)	0.60357 (10)	0.0347 (5)	
C18D	0.13375 (15)	0.21554 (15)	0.57566 (12)	0.0427 (6)	
H18D	0.171551	0.213136	0.537895	0.051*	
C19D	0.10765 (17)	0.15413 (16)	0.60326 (14)	0.0499 (7)	
H19D	0.127940	0.109027	0.584587	0.060*	
C20D	0.05136 (17)	0.15683 (16)	0.65874 (14)	0.0496 (7)	
H20D	0.034808	0.113615	0.677709	0.060*	
C21D	0.02018 (16)	0.22111 (16)	0.68569 (12)	0.0459 (6)	
H21D	−0.019291	0.222493	0.722653	0.055*	
C1E	0.40588 (12)	−0.05922 (13)	0.22904 (9)	0.0293 (4)	
H1E	0.375978	−0.099365	0.234913	0.035*	
C2E	0.42081 (12)	−0.03066 (12)	0.27683 (9)	0.0294 (4)	
C3E	0.46503 (12)	0.02748 (13)	0.26633 (9)	0.0304 (5)	
C4E	0.49151 (12)	0.05862 (13)	0.21222 (10)	0.0323 (5)	
H4E	0.520537	0.099363	0.206545	0.039*	
C5E	0.47444 (12)	0.02863 (13)	0.16665 (9)	0.0309 (5)	
C6E	0.43294 (12)	−0.03140 (13)	0.17319 (9)	0.0297 (4)	
C7E	0.38990 (12)	−0.06223 (12)	0.33768 (9)	0.0299 (5)	
H7E	0.425382	−0.051981	0.358988	0.036*	
C8E	0.38705 (13)	−0.14379 (12)	0.33417 (9)	0.0310 (5)	
H8E1	0.352236	−0.154253	0.312878	0.037*	
H8E2	0.438597	−0.164736	0.310787	0.037*	
C9E	0.36116 (13)	−0.18145 (13)	0.39231 (10)	0.0333 (5)	
H9E1	0.400386	−0.179640	0.411057	0.040*	
H9E2	0.313094	−0.156919	0.418655	0.040*	
C10E	0.34873 (14)	−0.25888 (13)	0.38193 (10)	0.0360 (5)	
H10I	0.306249	−0.259092	0.366383	0.043*	
H10J	0.395151	−0.280035	0.351216	0.043*	
C11E	0.33109 (16)	−0.30722 (14)	0.43376 (11)	0.0402 (6)	
H11I	0.283823	−0.287772	0.464536	0.048*	
H11J	0.373168	−0.308000	0.449799	0.048*	
C12E	0.32096 (17)	−0.38308 (15)	0.41733 (11)	0.0448 (6)	
H12I	0.275485	−0.382770	0.405229	0.054*	
H12J	0.365937	−0.400407	0.383589	0.054*	
C13E	0.3116 (3)	−0.43405 (19)	0.46671 (15)	0.0787 (12)	
H13M	0.357433	−0.436208	0.477763	0.118*	
H13N	0.304197	−0.481862	0.454202	0.118*	
H13O	0.267140	−0.417215	0.500262	0.118*	
C14E	0.45137 (13)	0.12072 (13)	0.33392 (10)	0.0332 (5)	
C15E	0.39008 (13)	0.12366 (13)	0.38895 (9)	0.0318 (5)	
C16E	0.37697 (15)	0.24449 (14)	0.38386 (11)	0.0390 (5)	
C17E	0.43713 (15)	0.24141 (14)	0.32955 (11)	0.0394 (5)	
C18E	0.45843 (18)	0.30495 (16)	0.29905 (13)	0.0499 (7)	
H18E	0.499308	0.303475	0.262833	0.060*	
C19E	0.4194 (2)	0.36885 (17)	0.32232 (15)	0.0583 (8)	
H19E	0.432434	0.411623	0.301473	0.070*	
C20E	0.3606 (2)	0.37169 (17)	0.37638 (16)	0.0616 (8)	
H20E	0.334773	0.416561	0.391984	0.074*	
C21E	0.33965 (18)	0.31108 (16)	0.40722 (14)	0.0532 (7)	
H21E	0.300129	0.313973	0.444175	0.064*	
C1F	0.24510 (12)	−0.04690 (12)	0.36547 (9)	0.0270 (4)	
H1F	0.248944	−0.087531	0.341055	0.032*	
C2F	0.17295 (12)	−0.01307 (12)	0.39400 (8)	0.0268 (4)	
C3F	0.16954 (12)	0.04688 (12)	0.42871 (9)	0.0280 (4)	
C4F	0.23374 (13)	0.07094 (12)	0.43632 (9)	0.0288 (4)	
H4F	0.229814	0.111255	0.461076	0.035*	
C5F	0.30404 (12)	0.03519 (12)	0.40715 (9)	0.0283 (4)	
C6F	0.31175 (12)	−0.02422 (12)	0.37091 (9)	0.0273 (4)	
C7F	0.10117 (12)	−0.04126 (12)	0.38785 (9)	0.0288 (4)	
H7F	0.057173	−0.021298	0.421983	0.035*	
C8F	0.10125 (13)	−0.12306 (13)	0.39126 (10)	0.0333 (5)	
H8F1	0.054714	−0.138188	0.385249	0.040*	
H8F2	0.145867	−0.144646	0.359162	0.040*	
C9F	0.10408 (17)	−0.15202 (16)	0.44882 (12)	0.0476 (5)	
H9F1	0.073385	−0.118381	0.480819	0.057*	
H9F2	0.157481	−0.154265	0.448056	0.057*	
C10F	0.07421 (17)	−0.22632 (16)	0.46164 (12)	0.0476 (5)	
H10K	0.075625	−0.240135	0.500439	0.057*	
H10L	0.020076	−0.222891	0.464178	0.057*	
C11F	0.1149 (3)	−0.28389 (19)	0.41960 (16)	0.0722 (10)	
H11K	0.168918	−0.287703	0.417271	0.087*	
H11L	0.113754	−0.270054	0.380724	0.087*	
C12F	0.0839 (3)	−0.3583 (2)	0.4328 (2)	0.0788 (11)	
H12K	0.029712	−0.354317	0.435373	0.095*	
H12L	0.112114	−0.390944	0.399373	0.095*	
C13F	0.0883 (3)	−0.3906 (3)	0.4849 (2)	0.0902 (13)	
H13P	0.141648	−0.395439	0.483029	0.135*	
H13Q	0.068351	−0.438032	0.488431	0.135*	
H13R	0.058033	−0.360442	0.518790	0.135*	
C14F	0.08770 (13)	0.14770 (13)	0.43293 (9)	0.0328 (5)	
C15F	0.04347 (12)	0.14889 (13)	0.39488 (9)	0.0326 (5)	
C16F	0.06255 (14)	0.26659 (14)	0.37929 (10)	0.0374 (5)	
C17F	0.10295 (14)	0.26631 (14)	0.41893 (11)	0.0378 (5)	
C18F	0.13064 (16)	0.33015 (15)	0.43138 (13)	0.0460 (6)	
H18F	0.157022	0.330496	0.458470	0.055*	
C19F	0.11933 (17)	0.39162 (16)	0.40427 (14)	0.0521 (7)	
H19F	0.138009	0.434593	0.412829	0.062*	
C20F	0.08065 (17)	0.39244 (16)	0.36400 (14)	0.0525 (7)	
H20F	0.073960	0.435525	0.345181	0.063*	
C21F	0.05249 (16)	0.33086 (15)	0.35186 (12)	0.0461 (6)	
H21F	0.026049	0.331597	0.324782	0.055*	
C1G	0.11704 (12)	−0.04964 (12)	0.27926 (9)	0.0285 (4)	
H1G	0.149514	−0.091963	0.275966	0.034*	
C2G	0.10085 (12)	−0.02642 (12)	0.23005 (9)	0.0285 (4)	
C3G	0.05381 (12)	0.03571 (13)	0.23635 (9)	0.0312 (5)	
C4G	0.02417 (12)	0.07484 (13)	0.28833 (10)	0.0322 (5)	
H4G	−0.007624	0.117573	0.291372	0.039*	
C5G	0.04225 (12)	0.04985 (13)	0.33565 (9)	0.0314 (5)	
C6G	0.08738 (12)	−0.01307 (12)	0.33324 (9)	0.0286 (4)	
C7G	0.13341 (12)	−0.06716 (12)	0.17120 (9)	0.0291 (4)	
H7G	0.097170	−0.056414	0.149631	0.035*	
C8G	0.13865 (14)	−0.14829 (13)	0.17876 (9)	0.0333 (5)	
H8G1	0.175207	−0.159948	0.199354	0.040*	
H8G2	0.088090	−0.162174	0.204138	0.040*	
C9G	0.16331 (14)	−0.19387 (14)	0.12258 (10)	0.0362 (5)	
H9G1	0.126200	−0.184260	0.102147	0.043*	
H9G2	0.213770	−0.180529	0.096563	0.043*	
C10G	0.16807 (15)	−0.27291 (14)	0.13536 (10)	0.0386 (5)	
H10M	0.118003	−0.284838	0.163011	0.046*	
H10N	0.206120	−0.281797	0.155032	0.046*	
C11G	0.18948 (17)	−0.32324 (15)	0.08259 (11)	0.0433 (6)	
H11M	0.149321	−0.317600	0.064619	0.052*	
H11N	0.237718	−0.309436	0.053499	0.052*	
C12G	0.19931 (18)	−0.40085 (15)	0.09693 (12)	0.0482 (6)	
H12M	0.150953	−0.414655	0.125853	0.058*	
H12N	0.239188	−0.406342	0.115192	0.058*	
C13G	0.2212 (3)	−0.45132 (19)	0.04451 (17)	0.0817 (12)	
H13S	0.180048	−0.449230	0.028045	0.123*	
H13T	0.229503	−0.500274	0.056448	0.123*	
H13U	0.268162	−0.437159	0.015071	0.123*	
C14G	0.06437 (13)	0.11785 (14)	0.16106 (10)	0.0333 (5)	
C15G	0.12192 (13)	0.10842 (13)	0.10437 (9)	0.0313 (5)	
C16G	0.12481 (13)	0.22918 (13)	0.09740 (10)	0.0332 (5)	
C17G	0.07168 (14)	0.23797 (14)	0.15489 (10)	0.0360 (5)	
C18G	0.05290 (15)	0.30712 (15)	0.18035 (11)	0.0443 (6)	
H18G	0.017956	0.313729	0.219058	0.053*	
C19G	0.08434 (16)	0.36456 (15)	0.15000 (12)	0.0443 (6)	
H19G	0.072374	0.410866	0.168088	0.053*	
C20G	0.13404 (16)	0.35630 (15)	0.09253 (12)	0.0451 (6)	
H20G	0.153980	0.397245	0.071357	0.054*	
C21G	0.15443 (16)	0.28948 (15)	0.06624 (11)	0.0424 (6)	
H21G	0.188447	0.284237	0.027131	0.051*	
C1H	0.27750 (12)	−0.06322 (12)	0.14296 (9)	0.0280 (4)	
H1H	0.275954	−0.099674	0.170333	0.034*	
C2H	0.34798 (12)	−0.03711 (12)	0.11237 (9)	0.0278 (4)	
C3H	0.34745 (13)	0.01771 (12)	0.07375 (9)	0.0296 (5)	
C4H	0.28179 (13)	0.04321 (12)	0.06430 (9)	0.0298 (5)	
H4H	0.283296	0.079896	0.037118	0.036*	
C5H	0.21383 (13)	0.01474 (12)	0.09485 (9)	0.0286 (4)	
C6H	0.20926 (12)	−0.03866 (12)	0.13551 (9)	0.0280 (4)	
C7H	0.42147 (12)	−0.06688 (13)	0.12057 (9)	0.0308 (5)	
H7H	0.464410	−0.053927	0.085316	0.037*	
C8H	0.42761 (14)	−0.14867 (14)	0.12373 (10)	0.0370 (5)	
H8H1	0.476624	−0.163838	0.128990	0.044*	
H8H2	0.386127	−0.162883	0.158760	0.044*	
C9H	0.42328 (16)	−0.18934 (14)	0.07092 (12)	0.0468 (6)	
H9HC	0.460850	−0.172740	0.034651	0.056*	0.7
H9HD	0.371663	−0.181329	0.068254	0.056*	0.7
H9HA	0.368195	−0.190137	0.077045	0.056*	0.3
H9HB	0.444341	−0.158157	0.037327	0.056*	0.3
C10H	0.4416 (4)	−0.2713 (2)	0.0794 (3)	0.0563 (14)	0.7
H10O	0.495196	−0.278253	0.077934	0.068*	0.7
H10P	0.408740	−0.284250	0.118851	0.068*	0.7
C11H	0.4306 (3)	−0.3221 (2)	0.03576 (19)	0.0529 (10)	0.7
H11O	0.375260	−0.321878	0.042676	0.063*	0.7
H11P	0.454894	−0.303172	−0.003624	0.063*	0.7
C12H	0.4611 (5)	−0.3981 (3)	0.0355 (3)	0.097 (2)	0.7
H12O	0.449695	−0.425186	0.005017	0.116*	0.7
H12P	0.517363	−0.398955	0.024037	0.116*	0.7
C13H	0.4331 (5)	−0.4349 (3)	0.0888 (3)	0.115 (3)	0.7
H13V	0.456889	−0.483932	0.083498	0.173*	0.7
H13W	0.445547	−0.409919	0.119267	0.173*	0.7
H13$	0.377649	−0.436239	0.100183	0.173*	0.7
C10I	0.4559 (7)	−0.2606 (4)	0.0507 (5)	0.046 (2)	0.3
H10Q	0.510243	−0.265541	0.047700	0.055*	0.3
H10R	0.452765	−0.270735	0.011837	0.055*	0.3
C11I	0.4110 (7)	−0.3111 (6)	0.0944 (5)	0.058 (2)	0.3
H11Q	0.413609	−0.298508	0.133023	0.070*	0.3
H11R	0.356884	−0.304136	0.097133	0.070*	0.3
C12I	0.4359 (10)	−0.3855 (7)	0.0816 (7)	0.086 (4)	0.3
H12Q	0.412939	−0.412385	0.117565	0.104*	0.3
H12R	0.491896	−0.390610	0.072203	0.104*	0.3
C13I	0.4186 (8)	−0.4194 (7)	0.0349 (5)	0.087 (4)	0.3
H13Z	0.438348	−0.469666	0.030493	0.130*	0.3
H131	0.363144	−0.416600	0.043970	0.130*	0.3
H132	0.442413	−0.394739	−0.001570	0.130*	0.3
C14H	0.42146 (13)	0.11436 (14)	0.06413 (9)	0.0334 (5)	
C15H	0.46477 (13)	0.11917 (14)	0.10241 (10)	0.0335 (5)	
C16H	0.43306 (14)	0.23827 (15)	0.11409 (11)	0.0388 (5)	
C17H	0.39301 (14)	0.23471 (14)	0.07413 (10)	0.0382 (5)	
C18H	0.35794 (16)	0.29742 (16)	0.06018 (12)	0.0459 (6)	
H18H	0.331652	0.295334	0.033125	0.055*	
C19H	0.36159 (18)	0.36142 (17)	0.08550 (14)	0.0515 (7)	
H19H	0.338112	0.403866	0.075792	0.062*	
C20H	0.39997 (18)	0.36478 (17)	0.12589 (14)	0.0539 (7)	
H20H	0.401714	0.409604	0.143439	0.065*	
C21H	0.43475 (17)	0.30478 (16)	0.14032 (13)	0.0483 (6)	
H21H	0.460026	0.307896	0.167962	0.058*	
N1R	0.1892 (2)	0.11582 (17)	0.70789 (15)	0.0738 (8)	
C1R	0.24828 (16)	0.37466 (17)	0.76508 (13)	0.0496 (7)	
H1R	0.257286	0.420900	0.776131	0.060*	
C2R	0.23664 (17)	0.36444 (17)	0.71276 (13)	0.0498 (7)	
H2R	0.237774	0.403673	0.687866	0.060*	
C3R	0.22340 (15)	0.29752 (16)	0.69660 (11)	0.0447 (6)	
H3R	0.215834	0.290420	0.660467	0.054*	
C4R	0.22117 (14)	0.24027 (15)	0.73350 (11)	0.0404 (6)	
C5R	0.23358 (15)	0.25102 (16)	0.78595 (12)	0.0450 (6)	
H5R	0.232848	0.212023	0.810994	0.054*	
C6R	0.24682 (16)	0.31767 (17)	0.80135 (12)	0.0484 (7)	
H6R	0.255078	0.324889	0.837208	0.058*	
C7R	0.20425 (18)	0.17100 (18)	0.71861 (14)	0.0533 (7)	
N1S	0.26720 (15)	0.68326 (14)	0.75464 (11)	0.0511 (6)	
C1S	0.2464 (3)	0.9685 (2)	0.76040 (19)	0.0765 (11)	
H1S	0.242855	1.019376	0.761496	0.092*	
C2S	0.3163 (2)	0.93123 (19)	0.74963 (17)	0.0674 (9)	
H2S	0.360720	0.956475	0.743134	0.081*	
C3S	0.32186 (17)	0.85790 (17)	0.74830 (13)	0.0505 (7)	
H3S	0.369991	0.832072	0.740580	0.061*	
C4S	0.25634 (15)	0.82161 (15)	0.75835 (10)	0.0401 (6)	
C5S	0.18561 (17)	0.85922 (18)	0.76939 (13)	0.0537 (7)	
H5S	0.140775	0.834438	0.776666	0.064*	
C6S	0.1819 (2)	0.9327 (2)	0.76955 (18)	0.0742 (11)	
H6S	0.134253	0.958985	0.776055	0.089*	
C7S	0.26201 (15)	0.74465 (16)	0.75660 (11)	0.0427 (6)	
N1T	0.2058 (2)	0.38615 (18)	0.21522 (16)	0.0766 (9)	
C1T	0.26500 (18)	0.11411 (18)	0.26130 (14)	0.0554 (7)	
H1T	0.274420	0.065850	0.270489	0.066*	
C2T	0.26497 (18)	0.16903 (19)	0.29874 (12)	0.0559 (8)	
H2T	0.274544	0.158410	0.333790	0.067*	
C3T	0.25136 (17)	0.23856 (18)	0.28617 (13)	0.0524 (7)	
H3T	0.251791	0.276023	0.312276	0.063*	
C4T	0.23692 (15)	0.25449 (16)	0.23532 (12)	0.0437 (6)	
C5T	0.23741 (16)	0.19977 (17)	0.19669 (11)	0.0470 (6)	
H5T	0.228344	0.210611	0.161479	0.056*	
C6T	0.25112 (17)	0.12970 (17)	0.20978 (13)	0.0511 (7)	
H6T	0.251122	0.092025	0.183721	0.061*	
C7T	0.22001 (18)	0.32777 (18)	0.22347 (15)	0.0559 (7)	
N1U	0.26218 (14)	0.80041 (14)	0.25829 (10)	0.0480 (5)	
C1U	0.2880 (3)	0.5158 (2)	0.2582 (2)	0.0779 (11)	
H1U	0.293559	0.464948	0.258163	0.093*	
C2U	0.3495 (2)	0.5536 (2)	0.25224 (19)	0.0739 (10)	
H2U	0.397412	0.529096	0.248033	0.089*	
C3U	0.34262 (19)	0.62697 (17)	0.25232 (14)	0.0546 (7)	
H3U	0.385615	0.653504	0.248046	0.065*	
C4U	0.27186 (15)	0.66231 (15)	0.25873 (11)	0.0414 (6)	
C5U	0.20872 (19)	0.62417 (18)	0.26471 (14)	0.0553 (7)	
H5U	0.160576	0.648233	0.269032	0.066*	
C6U	0.2178 (2)	0.5494 (2)	0.26420 (18)	0.0722 (10)	
H6U	0.175616	0.521871	0.268003	0.087*	
C7U	0.26606 (14)	0.73938 (16)	0.25833 (11)	0.0395 (6)	

### Atomic displacement parameters (Å^2^)

**Table:**

	*U*^11^	*U*^22^	*U*^33^	*U*^12^	*U*^13^	*U*^23^
N1A	0.0352 (10)	0.0366 (12)	0.0405 (11)	−0.0068 (8)	−0.0126 (8)	0.0064 (9)
N2A	0.0401 (11)	0.0367 (12)	0.0336 (10)	−0.0073 (9)	−0.0114 (8)	0.0080 (8)
N1B	0.0430 (11)	0.0354 (12)	0.0292 (9)	0.0042 (9)	−0.0110 (8)	0.0005 (8)
N2B	0.0347 (10)	0.0417 (12)	0.0333 (10)	0.0056 (9)	−0.0081 (8)	−0.0030 (9)
N1C	0.0364 (10)	0.0385 (12)	0.0340 (10)	0.0045 (8)	−0.0102 (8)	−0.0077 (9)
N2C	0.0377 (10)	0.0383 (12)	0.0299 (9)	0.0015 (8)	−0.0080 (8)	−0.0080 (8)
N1D	0.0372 (10)	0.0341 (11)	0.0309 (9)	−0.0040 (8)	−0.0126 (8)	−0.0023 (8)
N2D	0.0424 (11)	0.0384 (12)	0.0331 (10)	−0.0112 (9)	−0.0122 (8)	0.0025 (8)
N1E	0.0426 (11)	0.0395 (12)	0.0331 (10)	−0.0123 (9)	−0.0123 (8)	0.0005 (9)
N2E	0.0404 (11)	0.0369 (12)	0.0344 (10)	−0.0072 (9)	−0.0117 (8)	−0.0050 (8)
N1F	0.0380 (10)	0.0324 (11)	0.0328 (10)	0.0027 (8)	−0.0085 (8)	−0.0035 (8)
N2F	0.0330 (10)	0.0387 (12)	0.0340 (10)	0.0047 (8)	−0.0059 (8)	0.0013 (8)
N1G	0.0347 (10)	0.0409 (12)	0.0307 (10)	0.0071 (9)	−0.0047 (8)	0.0028 (8)
N2G	0.0401 (10)	0.0354 (11)	0.0237 (9)	0.0007 (8)	−0.0091 (8)	0.0015 (8)
N1H	0.0407 (11)	0.0389 (12)	0.0314 (10)	−0.0116 (9)	−0.0107 (8)	0.0074 (8)
N2H	0.0377 (11)	0.0422 (13)	0.0370 (10)	−0.0099 (9)	−0.0121 (9)	0.0041 (9)
O1A	0.0311 (8)	0.0367 (9)	0.0271 (7)	−0.0008 (7)	−0.0029 (6)	0.0053 (6)
O2A	0.0335 (8)	0.0386 (10)	0.0246 (7)	−0.0053 (7)	−0.0035 (6)	0.0027 (6)
O1B	0.0383 (8)	0.0358 (9)	0.0280 (7)	0.0023 (7)	−0.0135 (6)	−0.0036 (6)
O2B	0.0333 (8)	0.0411 (10)	0.0303 (8)	0.0000 (7)	−0.0103 (6)	−0.0012 (7)
O1C	0.0312 (8)	0.0381 (10)	0.0281 (8)	−0.0002 (7)	−0.0028 (6)	−0.0092 (7)
O2C	0.0326 (8)	0.0358 (9)	0.0248 (7)	0.0004 (7)	−0.0015 (6)	−0.0039 (6)
O1D	0.0355 (8)	0.0332 (9)	0.0278 (7)	−0.0050 (6)	−0.0115 (6)	0.0029 (6)
O2D	0.0372 (8)	0.0351 (9)	0.0295 (8)	−0.0014 (7)	−0.0111 (6)	−0.0016 (6)
O1E	0.0331 (8)	0.0384 (10)	0.0312 (8)	−0.0040 (7)	−0.0111 (6)	−0.0024 (7)
O2E	0.0350 (8)	0.0357 (9)	0.0276 (7)	−0.0060 (7)	−0.0118 (6)	0.0009 (6)
O1F	0.0313 (8)	0.0329 (9)	0.0248 (7)	0.0041 (6)	−0.0027 (6)	−0.0006 (6)
O2F	0.0295 (8)	0.0366 (9)	0.0266 (7)	0.0022 (6)	−0.0013 (6)	−0.0027 (6)
O1G	0.0317 (8)	0.0429 (10)	0.0285 (8)	−0.0027 (7)	−0.0095 (6)	0.0075 (7)
O2G	0.0362 (8)	0.0333 (9)	0.0272 (7)	−0.0014 (7)	−0.0119 (6)	0.0004 (6)
O1H	0.0347 (8)	0.0383 (9)	0.0242 (7)	−0.0086 (7)	−0.0024 (6)	0.0008 (6)
O2H	0.0329 (8)	0.0398 (10)	0.0273 (7)	−0.0053 (7)	−0.0035 (6)	0.0032 (7)
C1A	0.0296 (10)	0.0244 (11)	0.0268 (10)	0.0018 (8)	−0.0042 (8)	−0.0002 (8)
C2A	0.0274 (10)	0.0280 (12)	0.0258 (10)	0.0049 (8)	−0.0037 (8)	−0.0004 (8)
C3A	0.0265 (10)	0.0290 (12)	0.0249 (10)	0.0026 (8)	−0.0013 (8)	0.0023 (8)
C4A	0.0269 (10)	0.0292 (12)	0.0307 (11)	−0.0001 (8)	−0.0044 (8)	0.0005 (9)
C5A	0.0296 (10)	0.0311 (12)	0.0268 (10)	0.0032 (9)	−0.0061 (8)	−0.0020 (9)
C6A	0.0282 (10)	0.0266 (11)	0.0253 (10)	0.0050 (8)	−0.0038 (8)	0.0005 (8)
C7A	0.0301 (10)	0.0317 (12)	0.0231 (10)	0.0000 (9)	−0.0023 (8)	−0.0011 (8)
C8A	0.0381 (12)	0.0326 (13)	0.0327 (11)	0.0010 (10)	−0.0045 (9)	−0.0049 (9)
C9A	0.0529 (16)	0.0496 (17)	0.0444 (15)	0.0015 (13)	−0.0093 (12)	−0.0198 (13)
C10A	0.102 (3)	0.065 (2)	0.075 (2)	0.004 (2)	−0.029 (2)	−0.024 (2)
C11A	0.085 (3)	0.083 (3)	0.062 (2)	−0.017 (2)	−0.0318 (19)	0.0046 (19)
C12A	0.092 (2)	0.0673 (19)	0.111 (2)	−0.0048 (15)	−0.0383 (19)	0.0002 (17)
C13A	0.092 (2)	0.0673 (19)	0.111 (2)	−0.0048 (15)	−0.0383 (19)	0.0002 (17)
C14A	0.0287 (10)	0.0360 (13)	0.0283 (10)	−0.0068 (9)	−0.0032 (8)	0.0067 (9)
C15A	0.0318 (11)	0.0392 (14)	0.0250 (10)	−0.0058 (9)	−0.0025 (8)	0.0051 (9)
C16A	0.0387 (12)	0.0383 (14)	0.0383 (12)	−0.0072 (10)	−0.0109 (10)	0.0089 (10)
C17A	0.0373 (12)	0.0349 (14)	0.0414 (13)	−0.0089 (10)	−0.0122 (10)	0.0053 (10)
C18A	0.0498 (15)	0.0424 (16)	0.0588 (17)	−0.0083 (12)	−0.0250 (13)	0.0024 (13)
C19A	0.0594 (18)	0.0366 (16)	0.072 (2)	−0.0091 (13)	−0.0246 (15)	−0.0024 (14)
C20A	0.0602 (18)	0.0361 (16)	0.0691 (19)	−0.0036 (13)	−0.0235 (15)	0.0092 (14)
C21A	0.0550 (16)	0.0384 (15)	0.0530 (16)	−0.0069 (12)	−0.0232 (13)	0.0116 (12)
C1B	0.0352 (11)	0.0254 (11)	0.0207 (9)	−0.0032 (8)	−0.0062 (8)	−0.0017 (8)
C2B	0.0345 (11)	0.0280 (12)	0.0225 (9)	−0.0049 (9)	−0.0065 (8)	−0.0053 (8)
C3B	0.0345 (11)	0.0308 (12)	0.0230 (9)	0.0008 (9)	−0.0102 (8)	−0.0059 (8)
C4B	0.0414 (12)	0.0280 (12)	0.0203 (9)	−0.0038 (9)	−0.0077 (8)	−0.0021 (8)
C5B	0.0329 (11)	0.0295 (12)	0.0204 (9)	−0.0046 (9)	−0.0039 (8)	−0.0028 (8)
C6B	0.0354 (11)	0.0261 (11)	0.0200 (9)	−0.0006 (9)	−0.0049 (8)	−0.0040 (8)
C7B	0.0315 (11)	0.0348 (13)	0.0258 (10)	−0.0037 (9)	−0.0077 (8)	−0.0021 (9)
C8B	0.0391 (12)	0.0355 (13)	0.0297 (11)	−0.0093 (10)	−0.0069 (9)	−0.0031 (9)
C9B	0.0465 (13)	0.0352 (14)	0.0316 (11)	−0.0094 (10)	−0.0110 (10)	−0.0054 (10)
C10B	0.0453 (13)	0.0365 (14)	0.0316 (11)	−0.0070 (10)	−0.0106 (10)	−0.0041 (10)
C11B	0.0512 (14)	0.0367 (14)	0.0310 (11)	−0.0070 (11)	−0.0125 (10)	−0.0042 (10)
C12B	0.0583 (16)	0.0381 (15)	0.0344 (12)	−0.0063 (12)	−0.0115 (11)	−0.0023 (11)
C13B	0.179 (5)	0.0340 (18)	0.0440 (17)	0.009 (2)	−0.034 (2)	−0.0076 (14)
C14B	0.0356 (11)	0.0367 (13)	0.0289 (11)	0.0046 (10)	−0.0138 (9)	−0.0023 (9)
C15B	0.0299 (11)	0.0389 (14)	0.0316 (11)	0.0039 (9)	−0.0109 (9)	−0.0001 (10)
C16B	0.0352 (12)	0.0386 (14)	0.0357 (12)	0.0081 (10)	−0.0119 (10)	−0.0006 (10)
C17B	0.0431 (13)	0.0337 (13)	0.0351 (12)	0.0082 (10)	−0.0137 (10)	0.0009 (10)
C18B	0.0606 (17)	0.0383 (15)	0.0373 (13)	0.0045 (12)	−0.0067 (12)	0.0050 (11)
C19B	0.0689 (19)	0.0333 (15)	0.0543 (17)	0.0038 (13)	−0.0125 (14)	0.0058 (12)
C20B	0.0586 (17)	0.0352 (15)	0.0552 (16)	0.0117 (12)	−0.0178 (13)	−0.0079 (12)
C21B	0.0442 (14)	0.0417 (15)	0.0421 (13)	0.0107 (11)	−0.0113 (11)	−0.0066 (11)
C1C	0.0283 (10)	0.0301 (12)	0.0283 (10)	−0.0030 (8)	−0.0046 (8)	−0.0039 (9)
C2C	0.0246 (10)	0.0344 (13)	0.0275 (10)	−0.0044 (8)	−0.0034 (8)	−0.0023 (9)
C3C	0.0257 (10)	0.0384 (13)	0.0265 (10)	−0.0010 (9)	−0.0034 (8)	−0.0088 (9)
C4C	0.0274 (10)	0.0335 (13)	0.0320 (11)	0.0017 (9)	−0.0062 (8)	−0.0046 (9)
C5C	0.0254 (10)	0.0384 (13)	0.0297 (11)	−0.0023 (9)	−0.0068 (8)	−0.0017 (9)
C6C	0.0261 (10)	0.0356 (13)	0.0281 (10)	−0.0049 (9)	−0.0062 (8)	−0.0049 (9)
C7C	0.0296 (10)	0.0326 (13)	0.0244 (10)	−0.0040 (9)	−0.0013 (8)	−0.0022 (9)
C8C	0.0366 (12)	0.0366 (14)	0.0334 (11)	−0.0089 (10)	−0.0059 (9)	−0.0015 (10)
C9C	0.0387 (13)	0.0406 (15)	0.0411 (13)	−0.0099 (11)	−0.0081 (10)	0.0075 (11)
C10C	0.0597 (18)	0.0480 (19)	0.077 (2)	−0.0182 (14)	−0.0272 (16)	0.0127 (16)
C11C	0.0522 (16)	0.0534 (19)	0.0483 (15)	−0.0098 (13)	−0.0105 (13)	0.0007 (13)
C12C	0.073 (2)	0.0448 (19)	0.085 (2)	−0.0119 (16)	−0.0245 (19)	−0.0144 (17)
C13C	0.070 (2)	0.062 (2)	0.077 (2)	0.0021 (17)	−0.0174 (19)	−0.0172 (19)
C14C	0.0290 (10)	0.0344 (13)	0.0259 (10)	0.0036 (9)	−0.0019 (8)	−0.0077 (9)
C15C	0.0318 (11)	0.0344 (13)	0.0241 (10)	0.0031 (9)	−0.0007 (8)	−0.0059 (9)
C16C	0.0354 (12)	0.0353 (13)	0.0318 (11)	0.0026 (10)	−0.0053 (9)	−0.0060 (10)
C17C	0.0362 (12)	0.0347 (13)	0.0346 (12)	0.0041 (10)	−0.0064 (9)	−0.0060 (10)
C18C	0.0525 (15)	0.0422 (16)	0.0466 (14)	0.0039 (12)	−0.0184 (12)	−0.0007 (12)
C19C	0.0605 (18)	0.0378 (16)	0.0585 (17)	−0.0011 (13)	−0.0179 (14)	0.0026 (13)
C20C	0.0567 (17)	0.0391 (16)	0.0583 (17)	−0.0072 (13)	−0.0184 (14)	−0.0066 (13)
C21C	0.0442 (14)	0.0436 (16)	0.0459 (14)	−0.0007 (11)	−0.0175 (11)	−0.0065 (12)
C1D	0.0341 (11)	0.0248 (11)	0.0228 (9)	−0.0040 (8)	−0.0045 (8)	0.0000 (8)
C2D	0.0341 (11)	0.0248 (11)	0.0208 (9)	−0.0016 (8)	−0.0049 (8)	0.0033 (8)
C3D	0.0329 (11)	0.0294 (12)	0.0214 (9)	−0.0043 (9)	−0.0079 (8)	0.0050 (8)
C4D	0.0383 (11)	0.0282 (12)	0.0209 (9)	−0.0024 (9)	−0.0067 (8)	−0.0010 (8)
C5D	0.0322 (11)	0.0301 (12)	0.0212 (9)	−0.0012 (9)	−0.0032 (8)	0.0012 (8)
C6D	0.0332 (11)	0.0285 (12)	0.0206 (9)	−0.0038 (9)	−0.0038 (8)	0.0029 (8)
C7D	0.0321 (11)	0.0276 (12)	0.0252 (10)	0.0010 (9)	−0.0074 (8)	0.0010 (8)
C8D	0.0362 (11)	0.0271 (12)	0.0250 (10)	0.0013 (9)	−0.0056 (9)	0.0007 (8)
C9D	0.0392 (12)	0.0296 (12)	0.0274 (10)	0.0004 (9)	−0.0064 (9)	0.0019 (9)
C10D	0.0497 (14)	0.0334 (14)	0.0294 (11)	−0.0016 (11)	−0.0073 (10)	0.0005 (10)
C11D	0.0422 (13)	0.0339 (13)	0.0338 (12)	−0.0013 (10)	−0.0094 (10)	0.0050 (10)
C12D	0.0533 (15)	0.0348 (14)	0.0425 (14)	−0.0042 (11)	−0.0112 (12)	0.0056 (11)
C13D	0.0676 (19)	0.0373 (16)	0.0611 (18)	−0.0067 (14)	−0.0191 (15)	0.0120 (13)
C14D	0.0342 (11)	0.0341 (13)	0.0278 (10)	−0.0065 (9)	−0.0129 (9)	0.0029 (9)
C15D	0.0330 (11)	0.0366 (13)	0.0302 (11)	−0.0040 (9)	−0.0117 (9)	−0.0004 (9)
C16D	0.0419 (13)	0.0354 (14)	0.0380 (12)	−0.0091 (10)	−0.0188 (10)	0.0000 (10)
C17D	0.0367 (12)	0.0355 (13)	0.0364 (12)	−0.0079 (10)	−0.0174 (10)	0.0000 (10)
C18D	0.0444 (14)	0.0386 (15)	0.0463 (14)	−0.0050 (11)	−0.0164 (11)	−0.0051 (11)
C19D	0.0536 (16)	0.0345 (15)	0.0678 (18)	−0.0077 (12)	−0.0278 (14)	−0.0019 (13)
C20D	0.0578 (17)	0.0365 (15)	0.0645 (18)	−0.0168 (12)	−0.0312 (14)	0.0098 (13)
C21D	0.0515 (15)	0.0439 (16)	0.0457 (14)	−0.0187 (12)	−0.0181 (12)	0.0081 (12)
C1E	0.0257 (10)	0.0302 (12)	0.0293 (10)	0.0003 (8)	−0.0064 (8)	−0.0010 (9)
C2E	0.0250 (10)	0.0320 (12)	0.0272 (10)	0.0031 (8)	−0.0049 (8)	−0.0019 (9)
C3E	0.0265 (10)	0.0344 (13)	0.0284 (10)	0.0004 (9)	−0.0075 (8)	−0.0049 (9)
C4E	0.0273 (10)	0.0343 (13)	0.0323 (11)	−0.0047 (9)	−0.0059 (9)	−0.0006 (9)
C5E	0.0254 (10)	0.0360 (13)	0.0268 (10)	−0.0013 (9)	−0.0033 (8)	0.0019 (9)
C6E	0.0258 (10)	0.0311 (12)	0.0286 (10)	0.0013 (8)	−0.0053 (8)	−0.0024 (9)
C7E	0.0289 (10)	0.0319 (12)	0.0272 (10)	−0.0002 (9)	−0.0077 (8)	−0.0011 (9)
C8E	0.0308 (11)	0.0314 (12)	0.0281 (10)	0.0029 (9)	−0.0077 (8)	−0.0028 (9)
C9E	0.0369 (12)	0.0308 (13)	0.0293 (11)	0.0038 (9)	−0.0088 (9)	−0.0014 (9)
C10E	0.0384 (12)	0.0324 (13)	0.0318 (11)	0.0006 (10)	−0.0055 (9)	−0.0018 (9)
C11E	0.0493 (14)	0.0340 (14)	0.0344 (12)	−0.0007 (11)	−0.0110 (10)	0.0027 (10)
C12E	0.0547 (16)	0.0398 (15)	0.0347 (12)	−0.0055 (12)	−0.0082 (11)	0.0034 (11)
C13E	0.143 (4)	0.0437 (19)	0.0490 (18)	−0.024 (2)	−0.029 (2)	0.0090 (14)
C14E	0.0342 (11)	0.0372 (13)	0.0310 (11)	−0.0078 (9)	−0.0137 (9)	−0.0023 (9)
C15E	0.0343 (11)	0.0357 (13)	0.0285 (10)	−0.0076 (9)	−0.0137 (9)	−0.0009 (9)
C16E	0.0444 (13)	0.0340 (14)	0.0424 (13)	−0.0100 (10)	−0.0183 (11)	−0.0026 (10)
C17E	0.0466 (14)	0.0374 (14)	0.0372 (12)	−0.0125 (11)	−0.0164 (11)	−0.0010 (10)
C18E	0.0634 (18)	0.0433 (17)	0.0451 (14)	−0.0192 (13)	−0.0183 (13)	0.0040 (12)
C19E	0.078 (2)	0.0383 (17)	0.0668 (19)	−0.0214 (15)	−0.0322 (17)	0.0086 (14)
C20E	0.070 (2)	0.0347 (17)	0.081 (2)	−0.0077 (14)	−0.0260 (18)	−0.0067 (15)
C21E	0.0574 (17)	0.0400 (16)	0.0589 (17)	−0.0097 (13)	−0.0143 (14)	−0.0097 (13)
C1F	0.0323 (10)	0.0248 (11)	0.0219 (9)	−0.0012 (8)	−0.0071 (8)	0.0002 (8)
C2F	0.0299 (10)	0.0274 (11)	0.0204 (9)	−0.0025 (8)	−0.0049 (8)	0.0048 (8)
C3F	0.0310 (10)	0.0289 (12)	0.0202 (9)	0.0013 (8)	−0.0044 (8)	0.0020 (8)
C4F	0.0373 (11)	0.0256 (11)	0.0209 (9)	−0.0005 (9)	−0.0071 (8)	−0.0004 (8)
C5F	0.0320 (11)	0.0316 (12)	0.0218 (9)	−0.0043 (9)	−0.0092 (8)	0.0030 (8)
C6F	0.0294 (10)	0.0288 (12)	0.0213 (9)	−0.0011 (8)	−0.0060 (8)	0.0031 (8)
C7F	0.0275 (10)	0.0320 (12)	0.0229 (9)	−0.0023 (8)	−0.0033 (8)	0.0012 (8)
C8F	0.0339 (11)	0.0330 (13)	0.0309 (11)	−0.0061 (9)	−0.0074 (9)	0.0031 (9)
C9F	0.0531 (11)	0.0460 (12)	0.0398 (10)	−0.0057 (9)	−0.0104 (8)	0.0067 (8)
C10F	0.0531 (11)	0.0460 (12)	0.0398 (10)	−0.0057 (9)	−0.0104 (8)	0.0067 (8)
C11F	0.102 (3)	0.052 (2)	0.064 (2)	−0.0063 (19)	−0.030 (2)	0.0050 (16)
C12F	0.094 (3)	0.047 (2)	0.094 (3)	−0.0084 (19)	−0.029 (2)	0.0078 (19)
C13F	0.083 (3)	0.085 (3)	0.088 (3)	−0.009 (2)	−0.010 (2)	−0.006 (2)
C14F	0.0316 (11)	0.0332 (13)	0.0257 (10)	0.0049 (9)	−0.0009 (8)	−0.0009 (9)
C15F	0.0277 (10)	0.0350 (13)	0.0272 (10)	0.0048 (9)	−0.0006 (8)	−0.0031 (9)
C16F	0.0336 (12)	0.0360 (14)	0.0360 (12)	0.0059 (10)	−0.0050 (9)	−0.0004 (10)
C17F	0.0367 (12)	0.0339 (13)	0.0355 (12)	0.0052 (10)	−0.0043 (10)	−0.0009 (10)
C18F	0.0486 (15)	0.0376 (15)	0.0517 (15)	0.0021 (11)	−0.0179 (12)	−0.0027 (12)
C19F	0.0541 (16)	0.0339 (15)	0.0656 (18)	−0.0021 (12)	−0.0173 (14)	0.0009 (13)
C20F	0.0551 (17)	0.0372 (16)	0.0602 (17)	0.0041 (12)	−0.0146 (14)	0.0096 (13)
C21F	0.0456 (14)	0.0432 (16)	0.0472 (14)	0.0053 (12)	−0.0145 (12)	0.0062 (12)
C1G	0.0261 (10)	0.0308 (12)	0.0267 (10)	−0.0038 (8)	−0.0061 (8)	0.0024 (8)
C2G	0.0254 (10)	0.0327 (12)	0.0255 (10)	−0.0068 (8)	−0.0053 (8)	0.0007 (8)
C3G	0.0261 (10)	0.0386 (13)	0.0274 (10)	−0.0051 (9)	−0.0066 (8)	0.0060 (9)
C4G	0.0266 (10)	0.0340 (13)	0.0314 (11)	0.0020 (9)	−0.0049 (8)	0.0023 (9)
C5G	0.0261 (10)	0.0361 (13)	0.0268 (10)	−0.0017 (9)	−0.0023 (8)	−0.0013 (9)
C6G	0.0246 (10)	0.0322 (12)	0.0264 (10)	−0.0056 (8)	−0.0049 (8)	0.0023 (9)
C7G	0.0314 (11)	0.0318 (12)	0.0235 (10)	−0.0060 (9)	−0.0080 (8)	0.0018 (8)
C8G	0.0390 (12)	0.0339 (13)	0.0268 (10)	−0.0111 (10)	−0.0094 (9)	0.0011 (9)
C9G	0.0437 (13)	0.0367 (14)	0.0281 (11)	−0.0088 (10)	−0.0109 (9)	0.0000 (9)
C10G	0.0473 (14)	0.0376 (14)	0.0303 (11)	−0.0082 (11)	−0.0115 (10)	−0.0018 (10)
C11G	0.0553 (15)	0.0384 (15)	0.0347 (12)	−0.0091 (12)	−0.0123 (11)	−0.0043 (11)
C12G	0.0564 (16)	0.0409 (16)	0.0420 (14)	−0.0071 (12)	−0.0092 (12)	−0.0062 (12)
C13G	0.133 (4)	0.042 (2)	0.066 (2)	−0.001 (2)	−0.030 (2)	−0.0169 (16)
C14G	0.0305 (11)	0.0400 (14)	0.0291 (11)	0.0005 (9)	−0.0105 (9)	0.0058 (9)
C15G	0.0328 (11)	0.0346 (13)	0.0276 (10)	0.0002 (9)	−0.0123 (9)	−0.0010 (9)
C16G	0.0361 (12)	0.0351 (13)	0.0277 (10)	0.0050 (9)	−0.0113 (9)	−0.0004 (9)
C17G	0.0356 (12)	0.0390 (14)	0.0301 (11)	0.0078 (10)	−0.0089 (9)	0.0017 (10)
C18G	0.0451 (14)	0.0427 (15)	0.0351 (12)	0.0147 (11)	−0.0042 (11)	−0.0035 (11)
C19G	0.0506 (15)	0.0354 (14)	0.0425 (13)	0.0122 (11)	−0.0132 (11)	−0.0038 (11)
C20G	0.0543 (15)	0.0349 (15)	0.0424 (14)	0.0042 (11)	−0.0131 (12)	0.0034 (11)
C21G	0.0514 (15)	0.0396 (15)	0.0299 (11)	0.0017 (11)	−0.0069 (10)	0.0014 (10)
C1H	0.0352 (11)	0.0247 (11)	0.0217 (9)	−0.0029 (8)	−0.0066 (8)	−0.0010 (8)
C2H	0.0309 (10)	0.0276 (11)	0.0212 (9)	−0.0011 (8)	−0.0043 (8)	−0.0044 (8)
C3H	0.0346 (11)	0.0291 (12)	0.0208 (9)	−0.0071 (9)	−0.0032 (8)	−0.0031 (8)
C4H	0.0395 (12)	0.0277 (12)	0.0200 (9)	−0.0053 (9)	−0.0067 (8)	0.0000 (8)
C5H	0.0350 (11)	0.0276 (12)	0.0219 (9)	−0.0008 (9)	−0.0083 (8)	−0.0041 (8)
C6H	0.0331 (11)	0.0277 (12)	0.0213 (9)	−0.0041 (8)	−0.0067 (8)	−0.0029 (8)
C7H	0.0290 (10)	0.0351 (13)	0.0242 (10)	−0.0011 (9)	−0.0039 (8)	−0.0019 (9)
C8H	0.0371 (12)	0.0355 (13)	0.0350 (12)	0.0039 (10)	−0.0094 (10)	−0.0051 (10)
C9H	0.0419 (14)	0.0483 (16)	0.0448 (14)	0.0019 (12)	−0.0090 (11)	−0.0180 (12)
C10H	0.074 (4)	0.050 (3)	0.050 (3)	0.015 (3)	−0.033 (3)	−0.022 (2)
C11H	0.068 (3)	0.038 (2)	0.057 (2)	−0.0016 (19)	−0.026 (2)	−0.0095 (18)
C12H	0.150 (7)	0.047 (3)	0.112 (5)	−0.001 (4)	−0.070 (5)	−0.005 (3)
C13H	0.164 (8)	0.083 (5)	0.116 (6)	−0.006 (5)	−0.071 (6)	−0.001 (4)
C10I	0.049 (5)	0.058 (5)	0.028 (5)	0.010 (4)	−0.012 (5)	−0.020 (4)
C11I	0.070 (7)	0.049 (5)	0.066 (6)	−0.012 (5)	−0.037 (5)	0.008 (5)
C12I	0.118 (10)	0.027 (5)	0.123 (11)	−0.002 (6)	−0.053 (9)	0.007 (6)
C13I	0.062 (7)	0.093 (10)	0.105 (10)	−0.032 (7)	−0.022 (7)	0.042 (6)
C14H	0.0326 (11)	0.0390 (14)	0.0246 (10)	−0.0107 (10)	−0.0029 (9)	0.0050 (9)
C15H	0.0298 (11)	0.0391 (14)	0.0281 (10)	−0.0085 (9)	−0.0042 (9)	0.0043 (9)
C16H	0.0376 (12)	0.0422 (15)	0.0359 (12)	−0.0131 (10)	−0.0096 (10)	0.0043 (10)
C17H	0.0397 (12)	0.0396 (14)	0.0334 (12)	−0.0106 (10)	−0.0084 (10)	0.0066 (10)
C18H	0.0521 (15)	0.0443 (16)	0.0439 (14)	−0.0094 (12)	−0.0186 (12)	0.0065 (12)
C19H	0.0551 (16)	0.0420 (16)	0.0583 (17)	−0.0046 (13)	−0.0205 (14)	0.0074 (13)
C20H	0.0594 (17)	0.0408 (17)	0.0632 (18)	−0.0082 (13)	−0.0222 (15)	−0.0065 (14)
C21H	0.0520 (16)	0.0449 (17)	0.0519 (15)	−0.0089 (12)	−0.0217 (13)	−0.0025 (12)
N1R	0.090 (2)	0.0508 (18)	0.093 (2)	−0.0011 (15)	−0.0485 (19)	−0.0074 (16)
C1R	0.0459 (15)	0.0445 (17)	0.0552 (16)	−0.0073 (12)	−0.0124 (12)	−0.0088 (13)
C2R	0.0486 (15)	0.0509 (18)	0.0479 (15)	−0.0044 (13)	−0.0135 (12)	0.0098 (13)
C3R	0.0432 (14)	0.0578 (18)	0.0333 (12)	−0.0001 (12)	−0.0139 (11)	0.0011 (11)
C4R	0.0361 (12)	0.0433 (15)	0.0403 (13)	0.0010 (10)	−0.0118 (10)	−0.0037 (11)
C5R	0.0430 (14)	0.0528 (17)	0.0405 (13)	−0.0033 (12)	−0.0161 (11)	0.0090 (12)
C6R	0.0449 (14)	0.064 (2)	0.0367 (13)	−0.0046 (13)	−0.0146 (11)	−0.0056 (12)
C7R	0.0557 (17)	0.0492 (19)	0.0591 (17)	0.0024 (14)	−0.0262 (14)	−0.0019 (14)
N1S	0.0620 (15)	0.0435 (16)	0.0483 (13)	−0.0071 (11)	−0.0187 (11)	−0.0014 (11)
C1S	0.097 (3)	0.0410 (19)	0.097 (3)	0.0030 (19)	−0.041 (2)	−0.0004 (18)
C2S	0.073 (2)	0.051 (2)	0.081 (2)	−0.0159 (17)	−0.0269 (19)	0.0113 (17)
C3S	0.0480 (15)	0.0498 (18)	0.0521 (16)	−0.0034 (13)	−0.0152 (13)	0.0006 (13)
C4S	0.0465 (14)	0.0407 (15)	0.0305 (11)	−0.0015 (11)	−0.0102 (10)	−0.0022 (10)
C5S	0.0461 (15)	0.059 (2)	0.0525 (16)	0.0038 (13)	−0.0143 (13)	−0.0076 (14)
C6S	0.072 (2)	0.064 (2)	0.087 (3)	0.0240 (19)	−0.033 (2)	−0.0110 (19)
C7S	0.0445 (14)	0.0503 (18)	0.0319 (12)	−0.0044 (12)	−0.0112 (10)	−0.0012 (11)
N1T	0.083 (2)	0.052 (2)	0.096 (2)	−0.0034 (16)	−0.0338 (19)	0.0034 (16)
C1T	0.0536 (17)	0.0491 (18)	0.0585 (18)	0.0015 (13)	−0.0136 (14)	0.0102 (14)
C2T	0.0558 (17)	0.076 (2)	0.0371 (14)	−0.0006 (15)	−0.0179 (13)	0.0055 (14)
C3T	0.0526 (16)	0.063 (2)	0.0439 (15)	−0.0039 (14)	−0.0194 (13)	−0.0099 (13)
C4T	0.0368 (13)	0.0471 (16)	0.0429 (14)	−0.0057 (11)	−0.0077 (11)	−0.0003 (11)
C5T	0.0457 (14)	0.0616 (19)	0.0339 (12)	−0.0037 (13)	−0.0140 (11)	0.0003 (12)
C6T	0.0511 (16)	0.0497 (18)	0.0495 (15)	−0.0010 (13)	−0.0139 (13)	−0.0151 (13)
C7T	0.0547 (17)	0.0468 (19)	0.0650 (19)	−0.0066 (14)	−0.0182 (15)	0.0018 (14)
N1U	0.0548 (14)	0.0434 (15)	0.0496 (13)	−0.0019 (11)	−0.0232 (11)	0.0012 (10)
C1U	0.090 (3)	0.044 (2)	0.100 (3)	−0.0017 (19)	−0.034 (2)	−0.0030 (19)
C2U	0.076 (2)	0.048 (2)	0.102 (3)	0.0074 (17)	−0.039 (2)	−0.0107 (19)
C3U	0.0583 (17)	0.0434 (17)	0.0623 (18)	0.0016 (13)	−0.0220 (15)	−0.0053 (14)
C4U	0.0488 (14)	0.0409 (15)	0.0321 (12)	−0.0055 (11)	−0.0104 (10)	−0.0009 (10)
C5U	0.0550 (17)	0.0530 (19)	0.0551 (17)	−0.0109 (14)	−0.0141 (14)	0.0027 (14)
C6U	0.073 (2)	0.059 (2)	0.084 (2)	−0.0265 (18)	−0.0212 (19)	0.0023 (19)
C7U	0.0394 (13)	0.0449 (17)	0.0339 (12)	−0.0022 (11)	−0.0123 (10)	−0.0017 (10)

### Geometric parameters (Å, º)

**Table:**

N1A—C14A	1.288 (3)	C1E—H1E	0.9500
N1A—C17A	1.377 (3)	C2E—C3E	1.384 (3)
N2A—C15A	1.293 (3)	C2E—C7E	1.533 (3)
N2A—C16A	1.370 (3)	C3E—C4E	1.387 (3)
N1B—C14B	1.296 (3)	C4E—C5E	1.386 (3)
N1B—C17B	1.372 (3)	C4E—H4E	0.9500
N2B—C15B	1.289 (3)	C5E—C6E	1.389 (3)
N2B—C16B	1.373 (3)	C6E—C7H	1.531 (3)
N1C—C14C	1.291 (3)	C7E—C6F	1.531 (3)
N1C—C17C	1.373 (3)	C7E—C8E	1.535 (3)
N2C—C15C	1.292 (3)	C7E—H7E	1.0000
N2C—C16C	1.378 (3)	C8E—C9E	1.524 (3)
N1D—C14D	1.291 (3)	C8E—H8E1	0.9900
N1D—C17D	1.377 (3)	C8E—H8E2	0.9900
N2D—C15D	1.292 (3)	C9E—C10E	1.519 (3)
N2D—C16D	1.373 (3)	C9E—H9E1	0.9900
N1E—C14E	1.296 (3)	C9E—H9E2	0.9900
N1E—C17E	1.371 (3)	C10E—C11E	1.512 (3)
N2E—C15E	1.290 (3)	C10E—H10I	0.9900
N2E—C16E	1.371 (3)	C10E—H10J	0.9900
N1F—C14F	1.291 (3)	C11E—C12E	1.518 (4)
N1F—C17F	1.372 (3)	C11E—H11I	0.9900
N2F—C15F	1.296 (3)	C11E—H11J	0.9900
N2F—C16F	1.369 (3)	C12E—C13E	1.508 (4)
N1G—C14G	1.289 (3)	C12E—H12I	0.9900
N1G—C17G	1.374 (3)	C12E—H12J	0.9900
N2G—C15G	1.292 (3)	C13E—H13M	0.9800
N2G—C16G	1.377 (3)	C13E—H13N	0.9800
N1H—C14H	1.293 (3)	C13E—H13O	0.9800
N1H—C17H	1.378 (3)	C14E—C15E	1.436 (3)
N2H—C15H	1.292 (3)	C16E—C21E	1.405 (4)
N2H—C16H	1.363 (4)	C16E—C17E	1.414 (4)
O1A—C14A	1.367 (3)	C17E—C18E	1.412 (4)
O1A—C3A	1.406 (3)	C18E—C19E	1.372 (5)
O2A—C15A	1.376 (3)	C18E—H18E	0.9500
O2A—C5B	1.407 (3)	C19E—C20E	1.398 (5)
O1B—C14B	1.374 (3)	C19E—H19E	0.9500
O1B—C3B	1.416 (3)	C20E—C21E	1.367 (5)
O2B—C15B	1.374 (3)	C20E—H20E	0.9500
O2B—C5C	1.403 (3)	C21E—H21E	0.9500
O1C—C14C	1.368 (3)	C1F—C6F	1.392 (3)
O1C—C3C	1.406 (3)	C1F—C2F	1.395 (3)
O2C—C15C	1.377 (3)	C1F—H1F	0.9500
O2C—C5D	1.409 (3)	C2F—C3F	1.391 (3)
O1D—C14D	1.374 (3)	C2F—C7F	1.529 (3)
O1D—C3D	1.406 (3)	C3F—C4F	1.380 (3)
O2D—C15D	1.370 (3)	C4F—C5F	1.386 (3)
O2D—C5A	1.411 (3)	C4F—H4F	0.9500
O1E—C14E	1.367 (3)	C5F—C6F	1.390 (3)
O1E—C3E	1.411 (3)	C7F—C6G	1.528 (3)
O2E—C15E	1.370 (3)	C7F—C8F	1.534 (3)
O2E—C5F	1.411 (3)	C7F—H7F	1.0000
O1F—C14F	1.376 (3)	C8F—C9F	1.522 (4)
O1F—C3F	1.406 (3)	C8F—H8F1	0.9900
O2F—C15F	1.364 (3)	C8F—H8F2	0.9900
O2F—C5G	1.411 (3)	C9F—C10F	1.528 (4)
O1G—C14G	1.372 (3)	C9F—H9F1	0.9900
O1G—C3G	1.410 (3)	C9F—H9F2	0.9900
O2G—C15G	1.374 (3)	C10F—C11F	1.465 (5)
O2G—C5H	1.410 (3)	C10F—H10K	0.9900
O1H—C14H	1.375 (3)	C10F—H10L	0.9900
O1H—C3H	1.407 (3)	C11F—C12F	1.538 (5)
O2H—C15H	1.370 (3)	C11F—H11K	0.9900
O2H—C5E	1.401 (3)	C11F—H11L	0.9900
C1A—C2A	1.395 (3)	C12F—C13F	1.433 (6)
C1A—C6A	1.402 (3)	C12F—H12K	0.9900
C1A—H1A	0.9500	C12F—H12L	0.9900
C2A—C3A	1.391 (3)	C13F—H13P	0.9800
C2A—C7A	1.531 (3)	C13F—H13Q	0.9800
C3A—C4A	1.385 (3)	C13F—H13R	0.9800
C4A—C5A	1.392 (3)	C14F—C15F	1.438 (3)
C4A—H4A	0.9500	C16F—C21F	1.409 (4)
C5A—C6A	1.388 (3)	C16F—C17F	1.415 (4)
C6A—C7D	1.530 (3)	C17F—C18F	1.408 (4)
C7A—C6B	1.528 (3)	C18F—C19F	1.367 (4)
C7A—C8A	1.535 (3)	C18F—H18F	0.9500
C7A—H7A	1.0000	C19F—C20F	1.404 (5)
C8A—C9A	1.530 (4)	C19F—H19F	0.9500
C8A—H8A1	0.9900	C20F—C21F	1.373 (4)
C8A—H8A2	0.9900	C20F—H20F	0.9500
C9A—C10A	1.508 (5)	C21F—H21F	0.9500
C9A—H9A1	0.9900	C1G—C2G	1.397 (3)
C9A—H9A2	0.9900	C1G—C6G	1.400 (3)
C10A—C11A	1.447 (6)	C1G—H1G	0.9500
C10A—H10A	0.9900	C2G—C3G	1.384 (3)
C10A—H10B	0.9900	C2G—C7G	1.534 (3)
C11A—C12A	1.588 (6)	C3G—C4G	1.385 (3)
C11A—H11A	0.9900	C4G—C5G	1.382 (3)
C11A—H11B	0.9900	C4G—H4G	0.9500
C12A—C13A	1.450 (6)	C5G—C6G	1.389 (3)
C12A—H12A	0.9900	C7G—C6H	1.523 (3)
C12A—H12B	0.9900	C7G—C8G	1.531 (3)
C13A—H13A	0.9800	C7G—H7G	1.0000
C13A—H13B	0.9800	C8G—C9G	1.528 (3)
C13A—H13C	0.9800	C8G—H8G1	0.9900
C14A—C15A	1.437 (3)	C8G—H8G2	0.9900
C16A—C21A	1.408 (4)	C9G—C10G	1.516 (4)
C16A—C17A	1.417 (4)	C9G—H9G1	0.9900
C17A—C18A	1.405 (4)	C9G—H9G2	0.9900
C18A—C19A	1.364 (4)	C10G—C11G	1.517 (3)
C18A—H18A	0.9500	C10G—H10M	0.9900
C19A—C20A	1.404 (5)	C10G—H10N	0.9900
C19A—H19A	0.9500	C11G—C12G	1.508 (4)
C20A—C21A	1.364 (4)	C11G—H11M	0.9900
C20A—H20A	0.9500	C11G—H11N	0.9900
C21A—H21A	0.9500	C12G—C13G	1.512 (4)
C1B—C2B	1.395 (3)	C12G—H12M	0.9900
C1B—C6B	1.395 (3)	C12G—H12N	0.9900
C1B—H1B	0.9500	C13G—H13S	0.9800
C2B—C3B	1.387 (3)	C13G—H13T	0.9800
C2B—C7B	1.529 (3)	C13G—H13U	0.9800
C3B—C4B	1.381 (3)	C14G—C15G	1.436 (3)
C4B—C5B	1.384 (3)	C16G—C21G	1.400 (4)
C4B—H4B	0.9500	C16G—C17G	1.417 (3)
C5B—C6B	1.393 (3)	C17G—C18G	1.405 (4)
C7B—C8B	1.530 (3)	C18G—C19G	1.358 (4)
C7B—C6C	1.534 (3)	C18G—H18G	0.9500
C7B—H7B	1.0000	C19G—C20G	1.395 (4)
C8B—C9B	1.527 (3)	C19G—H19G	0.9500
C8B—H8B1	0.9900	C20G—C21G	1.375 (4)
C8B—H8B2	0.9900	C20G—H20G	0.9500
C9B—C10B	1.522 (4)	C21G—H21G	0.9500
C9B—H9B1	0.9900	C1H—C6H	1.396 (3)
C9B—H9B2	0.9900	C1H—C2H	1.397 (3)
C10B—C11B	1.522 (3)	C1H—H1H	0.9500
C10B—H10C	0.9900	C2H—C3H	1.395 (3)
C10B—H10D	0.9900	C2H—C7H	1.522 (3)
C11B—C12B	1.508 (4)	C3H—C4H	1.378 (3)
C11B—H11C	0.9900	C4H—C5H	1.380 (3)
C11B—H11D	0.9900	C4H—H4H	0.9500
C12B—C13B	1.517 (4)	C5H—C6H	1.395 (3)
C12B—H12C	0.9900	C7H—C8H	1.532 (3)
C12B—H12D	0.9900	C7H—H7H	1.0000
C13B—H13D	0.9800	C8H—C9H	1.525 (3)
C13B—H13E	0.9800	C8H—H8H1	0.9900
C13B—H13F	0.9800	C8H—H8H2	0.9900
C14B—C15B	1.430 (3)	C9H—C10I	1.441 (5)
C16B—C21B	1.409 (4)	C9H—C10H	1.578 (4)
C16B—C17B	1.414 (3)	C9H—H9HC	0.9900
C17B—C18B	1.410 (4)	C9H—H9HD	0.9900
C18B—C19B	1.369 (4)	C9H—H9HA	0.9900
C18B—H18B	0.9500	C9H—H9HB	0.9900
C19B—C20B	1.406 (4)	C10H—C11H	1.503 (6)
C19B—H19B	0.9500	C10H—H10O	0.9900
C20B—C21B	1.362 (4)	C10H—H10P	0.9900
C20B—H20B	0.9500	C11H—C12H	1.496 (8)
C21B—H21B	0.9500	C11H—H11O	0.9900
C1C—C6C	1.393 (3)	C11H—H11P	0.9900
C1C—C2C	1.399 (3)	C12H—C13H	1.423 (5)
C1C—H1C	0.9500	C12H—H12O	0.9900
C2C—C3C	1.392 (3)	C12H—H12P	0.9900
C2C—C7C	1.531 (3)	C13H—H13V	0.9800
C3C—C4C	1.382 (3)	C13H—H13W	0.9800
C4C—C5C	1.385 (3)	C13H—H13$	0.9800
C4C—H4C	0.9500	C10I—C11I	1.496 (16)
C5C—C6C	1.390 (3)	C10I—H10Q	0.9900
C7C—C6D	1.524 (3)	C10I—H10R	0.9900
C7C—C8C	1.540 (3)	C11I—C12I	1.437 (15)
C7C—H7C	1.0000	C11I—H11Q	0.9900
C8C—C9C	1.518 (4)	C11I—H11R	0.9900
C8C—H8C1	0.9900	C12I—C13I	1.446 (4)
C8C—H8C2	0.9900	C12I—H12Q	0.9900
C9C—C10C	1.544 (4)	C12I—H12R	0.9900
C9C—H9C1	0.9900	C13I—H13Z	0.9800
C9C—H9C2	0.9900	C13I—H131	0.9800
C10C—C11C	1.490 (5)	C13I—H132	0.9800
C10C—H10E	0.9900	C14H—C15H	1.436 (3)
C10C—H10F	0.9900	C16H—C21H	1.408 (4)
C11C—C12C	1.528 (4)	C16H—C17H	1.421 (4)
C11C—H11E	0.9900	C17H—C18H	1.403 (4)
C11C—H11F	0.9900	C18H—C19H	1.365 (4)
C12C—C13C	1.476 (6)	C18H—H18H	0.9500
C12C—H12E	0.9900	C19H—C20H	1.407 (4)
C12C—H12F	0.9900	C19H—H19H	0.9500
C13C—H13G	0.9800	C20H—C21H	1.365 (4)
C13C—H13H	0.9800	C20H—H20H	0.9500
C13C—H13I	0.9800	C21H—H21H	0.9500
C14C—C15C	1.429 (3)	N1R—C7R	1.143 (4)
C16C—C21C	1.403 (4)	C1R—C2R	1.381 (4)
C16C—C17C	1.415 (4)	C1R—C6R	1.382 (4)
C17C—C18C	1.410 (4)	C1R—H1R	0.9500
C18C—C19C	1.364 (4)	C2R—C3R	1.378 (4)
C18C—H18C	0.9500	C2R—H2R	0.9500
C19C—C20C	1.409 (4)	C3R—C4R	1.394 (4)
C19C—H19C	0.9500	C3R—H3R	0.9500
C20C—C21C	1.359 (4)	C4R—C5R	1.393 (4)
C20C—H20C	0.9500	C4R—C7R	1.432 (4)
C21C—H21C	0.9500	C5R—C6R	1.367 (4)
C1D—C6D	1.396 (3)	C5R—H5R	0.9500
C1D—C2D	1.398 (3)	C6R—H6R	0.9500
C1D—H1D	0.9500	N1S—C7S	1.147 (4)
C2D—C3D	1.395 (3)	C1S—C6S	1.372 (6)
C2D—C7D	1.528 (3)	C1S—C2S	1.380 (6)
C3D—C4D	1.384 (3)	C1S—H1S	0.9500
C4D—C5D	1.381 (3)	C2S—C3S	1.370 (5)
C4D—H4D	0.9500	C2S—H2S	0.9500
C5D—C6D	1.393 (3)	C3S—C4S	1.391 (4)
C7D—C8D	1.535 (3)	C3S—H3S	0.9500
C7D—H7D	1.0000	C4S—C5S	1.394 (4)
C8D—C9D	1.531 (3)	C4S—C7S	1.437 (4)
C8D—H8D1	0.9900	C5S—C6S	1.373 (5)
C8D—H8D2	0.9900	C5S—H5S	0.9500
C9D—C10D	1.521 (3)	C6S—H6S	0.9500
C9D—H9D1	0.9900	N1T—C7T	1.141 (4)
C9D—H9D2	0.9900	C1T—C2T	1.376 (5)
C10D—C11D	1.514 (3)	C1T—C6T	1.394 (4)
C10D—H10G	0.9900	C1T—H1T	0.9500
C10D—H10H	0.9900	C2T—C3T	1.364 (5)
C11D—C12D	1.515 (4)	C2T—H2T	0.9500
C11D—H11G	0.9900	C3T—C4T	1.385 (4)
C11D—H11H	0.9900	C3T—H3T	0.9500
C12D—C13D	1.510 (4)	C4T—C5T	1.390 (4)
C12D—H12G	0.9900	C4T—C7T	1.438 (5)
C12D—H12H	0.9900	C5T—C6T	1.378 (4)
C13D—H13J	0.9800	C5T—H5T	0.9500
C13D—H13K	0.9800	C6T—H6T	0.9500
C13D—H13L	0.9800	N1U—C7U	1.140 (4)
C14D—C15D	1.436 (3)	C1U—C2U	1.358 (6)
C16D—C17D	1.409 (3)	C1U—C6U	1.375 (6)
C16D—C21D	1.410 (4)	C1U—H1U	0.9500
C17D—C18D	1.408 (4)	C2U—C3U	1.372 (5)
C18D—C19D	1.367 (4)	C2U—H2U	0.9500
C18D—H18D	0.9500	C3U—C4U	1.396 (4)
C19D—C20D	1.401 (4)	C3U—H3U	0.9500
C19D—H19D	0.9500	C4U—C5U	1.388 (4)
C20D—C21D	1.365 (4)	C4U—C7U	1.440 (4)
C20D—H20D	0.9500	C5U—C6U	1.399 (5)
C21D—H21D	0.9500	C5U—H5U	0.9500
C1E—C6E	1.397 (3)	C6U—H6U	0.9500
C1E—C2E	1.402 (3)		
			
C14A—N1A—C17A	116.4 (2)	C11E—C10E—H10I	108.0
C15A—N2A—C16A	116.6 (2)	C9E—C10E—H10I	108.0
C14B—N1B—C17B	116.2 (2)	C11E—C10E—H10J	108.0
C15B—N2B—C16B	116.2 (2)	C9E—C10E—H10J	108.0
C14C—N1C—C17C	116.2 (2)	H10I—C10E—H10J	107.3
C15C—N2C—C16C	116.4 (2)	C10E—C11E—C12E	111.7 (2)
C14D—N1D—C17D	116.4 (2)	C10E—C11E—H11I	109.3
C15D—N2D—C16D	116.1 (2)	C12E—C11E—H11I	109.3
C14E—N1E—C17E	116.2 (2)	C10E—C11E—H11J	109.3
C15E—N2E—C16E	116.8 (2)	C12E—C11E—H11J	109.3
C14F—N1F—C17F	117.0 (2)	H11I—C11E—H11J	107.9
C15F—N2F—C16F	116.7 (2)	C13E—C12E—C11E	112.7 (2)
C14G—N1G—C17G	116.5 (2)	C13E—C12E—H12I	109.1
C15G—N2G—C16G	116.59 (19)	C11E—C12E—H12I	109.1
C14H—N1H—C17H	116.6 (2)	C13E—C12E—H12J	109.1
C15H—N2H—C16H	116.7 (2)	C11E—C12E—H12J	109.1
C14A—O1A—C3A	116.01 (16)	H12I—C12E—H12J	107.8
C15A—O2A—C5B	112.48 (16)	C12E—C13E—H13M	109.5
C14B—O1B—C3B	111.60 (17)	C12E—C13E—H13N	109.5
C15B—O2B—C5C	116.95 (17)	H13M—C13E—H13N	109.5
C14C—O1C—C3C	116.63 (17)	C12E—C13E—H13O	109.5
C15C—O2C—C5D	113.74 (16)	H13M—C13E—H13O	109.5
C14D—O1D—C3D	113.00 (17)	H13N—C13E—H13O	109.5
C15D—O2D—C5A	115.27 (17)	N1E—C14E—O1E	119.9 (2)
C14E—O1E—C3E	114.94 (17)	N1E—C14E—C15E	122.5 (2)
C15E—O2E—C5F	113.56 (17)	O1E—C14E—C15E	117.6 (2)
C14F—O1F—C3F	113.35 (16)	N2E—C15E—O2E	120.0 (2)
C15F—O2F—C5G	116.32 (17)	N2E—C15E—C14E	122.5 (2)
C14G—O1G—C3G	116.19 (18)	O2E—C15E—C14E	117.5 (2)
C15G—O2G—C5H	112.97 (16)	N2E—C16E—C21E	120.0 (2)
C14H—O1H—C3H	112.91 (16)	N2E—C16E—C17E	120.5 (2)
C15H—O2H—C5E	115.88 (17)	C21E—C16E—C17E	119.5 (3)
C2A—C1A—C6A	122.9 (2)	N1E—C17E—C18E	118.8 (2)
C2A—C1A—H1A	118.6	N1E—C17E—C16E	121.4 (2)
C6A—C1A—H1A	118.6	C18E—C17E—C16E	119.8 (3)
C3A—C2A—C1A	116.9 (2)	C19E—C18E—C17E	119.2 (3)
C3A—C2A—C7A	120.64 (19)	C19E—C18E—H18E	120.4
C1A—C2A—C7A	122.4 (2)	C17E—C18E—H18E	120.4
C4A—C3A—C2A	122.9 (2)	C18E—C19E—C20E	120.7 (3)
C4A—C3A—O1A	118.8 (2)	C18E—C19E—H19E	119.7
C2A—C3A—O1A	118.18 (19)	C20E—C19E—H19E	119.7
C3A—C4A—C5A	117.8 (2)	C21E—C20E—C19E	121.2 (3)
C3A—C4A—H4A	121.1	C21E—C20E—H20E	119.4
C5A—C4A—H4A	121.1	C19E—C20E—H20E	119.4
C6A—C5A—C4A	122.5 (2)	C20E—C21E—C16E	119.5 (3)
C6A—C5A—O2D	118.43 (19)	C20E—C21E—H21E	120.2
C4A—C5A—O2D	119.0 (2)	C16E—C21E—H21E	120.2
C5A—C6A—C1A	117.0 (2)	C6F—C1F—C2F	123.4 (2)
C5A—C6A—C7D	121.04 (19)	C6F—C1F—H1F	118.3
C1A—C6A—C7D	122.0 (2)	C2F—C1F—H1F	118.3
C6B—C7A—C2A	111.14 (17)	C3F—C2F—C1F	116.8 (2)
C6B—C7A—C8A	112.10 (19)	C3F—C2F—C7F	121.82 (19)
C2A—C7A—C8A	112.40 (19)	C1F—C2F—C7F	121.42 (19)
C6B—C7A—H7A	106.9	C4F—C3F—C2F	122.1 (2)
C2A—C7A—H7A	106.9	C4F—C3F—O1F	118.62 (19)
C8A—C7A—H7A	106.9	C2F—C3F—O1F	119.23 (19)
C9A—C8A—C7A	113.1 (2)	C3F—C4F—C5F	118.7 (2)
C9A—C8A—H8A1	109.0	C3F—C4F—H4F	120.6
C7A—C8A—H8A1	109.0	C5F—C4F—H4F	120.6
C9A—C8A—H8A2	109.0	C4F—C5F—C6F	122.1 (2)
C7A—C8A—H8A2	109.0	C4F—C5F—O2E	118.36 (19)
H8A1—C8A—H8A2	107.8	C6F—C5F—O2E	119.53 (19)
C10A—C9A—C8A	117.4 (3)	C5F—C6F—C1F	116.79 (19)
C10A—C9A—H9A1	108.0	C5F—C6F—C7E	121.71 (19)
C8A—C9A—H9A1	108.0	C1F—C6F—C7E	121.49 (19)
C10A—C9A—H9A2	108.0	C6G—C7F—C2F	111.31 (17)
C8A—C9A—H9A2	108.0	C6G—C7F—C8F	112.57 (18)
H9A1—C9A—H9A2	107.2	C2F—C7F—C8F	112.57 (18)
C11A—C10A—C9A	115.9 (4)	C6G—C7F—H7F	106.6
C11A—C10A—H10A	108.3	C2F—C7F—H7F	106.6
C9A—C10A—H10A	108.3	C8F—C7F—H7F	106.6
C11A—C10A—H10B	108.3	C9F—C8F—C7F	113.9 (2)
C9A—C10A—H10B	108.3	C9F—C8F—H8F1	108.8
H10A—C10A—H10B	107.4	C7F—C8F—H8F1	108.8
C10A—C11A—C12A	111.7 (3)	C9F—C8F—H8F2	108.8
C10A—C11A—H11A	109.3	C7F—C8F—H8F2	108.8
C12A—C11A—H11A	109.3	H8F1—C8F—H8F2	107.7
C10A—C11A—H11B	109.3	C8F—C9F—C10F	113.3 (2)
C12A—C11A—H11B	109.3	C8F—C9F—H9F1	108.9
H11A—C11A—H11B	107.9	C10F—C9F—H9F1	108.9
C13A—C12A—C11A	111.4 (4)	C8F—C9F—H9F2	108.9
C13A—C12A—H12A	109.4	C10F—C9F—H9F2	108.9
C11A—C12A—H12A	109.4	H9F1—C9F—H9F2	107.7
C13A—C12A—H12B	109.4	C11F—C10F—C9F	116.5 (3)
C11A—C12A—H12B	109.4	C11F—C10F—H10K	108.2
H12A—C12A—H12B	108.0	C9F—C10F—H10K	108.2
C12A—C13A—H13A	109.5	C11F—C10F—H10L	108.2
C12A—C13A—H13B	109.5	C9F—C10F—H10L	108.2
H13A—C13A—H13B	109.5	H10K—C10F—H10L	107.3
C12A—C13A—H13C	109.5	C10F—C11F—C12F	116.0 (3)
H13A—C13A—H13C	109.5	C10F—C11F—H11K	108.3
H13B—C13A—H13C	109.5	C12F—C11F—H11K	108.3
N1A—C14A—O1A	120.5 (2)	C10F—C11F—H11L	108.3
N1A—C14A—C15A	122.8 (2)	C12F—C11F—H11L	108.3
O1A—C14A—C15A	116.7 (2)	H11K—C11F—H11L	107.4
N2A—C15A—O2A	119.6 (2)	C13F—C12F—C11F	116.4 (4)
N2A—C15A—C14A	122.4 (2)	C13F—C12F—H12K	108.2
O2A—C15A—C14A	118.0 (2)	C11F—C12F—H12K	108.2
N2A—C16A—C21A	119.8 (2)	C13F—C12F—H12L	108.2
N2A—C16A—C17A	120.9 (2)	C11F—C12F—H12L	108.2
C21A—C16A—C17A	119.2 (3)	H12K—C12F—H12L	107.3
N1A—C17A—C18A	119.9 (2)	C12F—C13F—H13P	109.5
N1A—C17A—C16A	120.9 (2)	C12F—C13F—H13Q	109.5
C18A—C17A—C16A	119.3 (2)	H13P—C13F—H13Q	109.5
C19A—C18A—C17A	120.3 (3)	C12F—C13F—H13R	109.5
C19A—C18A—H18A	119.8	H13P—C13F—H13R	109.5
C17A—C18A—H18A	119.8	H13Q—C13F—H13R	109.5
C18A—C19A—C20A	120.3 (3)	N1F—C14F—O1F	119.8 (2)
C18A—C19A—H19A	119.8	N1F—C14F—C15F	122.4 (2)
C20A—C19A—H19A	119.8	O1F—C14F—C15F	117.8 (2)
C21A—C20A—C19A	120.8 (3)	N2F—C15F—O2F	120.7 (2)
C21A—C20A—H20A	119.6	N2F—C15F—C14F	122.2 (2)
C19A—C20A—H20A	119.6	O2F—C15F—C14F	117.0 (2)
C20A—C21A—C16A	120.0 (3)	N2F—C16F—C21F	119.9 (2)
C20A—C21A—H21A	120.0	N2F—C16F—C17F	121.1 (2)
C16A—C21A—H21A	120.0	C21F—C16F—C17F	119.0 (3)
C2B—C1B—C6B	123.6 (2)	N1F—C17F—C18F	119.7 (2)
C2B—C1B—H1B	118.2	N1F—C17F—C16F	120.5 (2)
C6B—C1B—H1B	118.2	C18F—C17F—C16F	119.8 (2)
C3B—C2B—C1B	116.7 (2)	C19F—C18F—C17F	119.6 (3)
C3B—C2B—C7B	122.0 (2)	C19F—C18F—H18F	120.2
C1B—C2B—C7B	121.3 (2)	C17F—C18F—H18F	120.2
C4B—C3B—C2B	122.3 (2)	C18F—C19F—C20F	121.3 (3)
C4B—C3B—O1B	117.7 (2)	C18F—C19F—H19F	119.4
C2B—C3B—O1B	120.0 (2)	C20F—C19F—H19F	119.4
C3B—C4B—C5B	118.9 (2)	C21F—C20F—C19F	119.9 (3)
C3B—C4B—H4B	120.5	C21F—C20F—H20F	120.0
C5B—C4B—H4B	120.5	C19F—C20F—H20F	120.0
C4B—C5B—C6B	122.0 (2)	C20F—C21F—C16F	120.5 (3)
C4B—C5B—O2A	118.1 (2)	C20F—C21F—H21F	119.8
C6B—C5B—O2A	119.9 (2)	C16F—C21F—H21F	119.8
C5B—C6B—C1B	116.6 (2)	C2G—C1G—C6G	122.7 (2)
C5B—C6B—C7A	121.6 (2)	C2G—C1G—H1G	118.7
C1B—C6B—C7A	121.8 (2)	C6G—C1G—H1G	118.7
C2B—C7B—C8B	113.04 (19)	C3G—C2G—C1G	117.1 (2)
C2B—C7B—C6C	109.02 (18)	C3G—C2G—C7G	120.7 (2)
C8B—C7B—C6C	111.34 (19)	C1G—C2G—C7G	122.2 (2)
C2B—C7B—H7B	107.7	C2G—C3G—C4G	122.7 (2)
C8B—C7B—H7B	107.7	C2G—C3G—O1G	117.9 (2)
C6C—C7B—H7B	107.7	C4G—C3G—O1G	119.3 (2)
C9B—C8B—C7B	116.4 (2)	C5G—C4G—C3G	118.0 (2)
C9B—C8B—H8B1	108.2	C5G—C4G—H4G	121.0
C7B—C8B—H8B1	108.2	C3G—C4G—H4G	121.0
C9B—C8B—H8B2	108.2	C4G—C5G—C6G	122.7 (2)
C7B—C8B—H8B2	108.2	C4G—C5G—O2F	119.1 (2)
H8B1—C8B—H8B2	107.3	C6G—C5G—O2F	118.1 (2)
C10B—C9B—C8B	110.2 (2)	C5G—C6G—C1G	116.8 (2)
C10B—C9B—H9B1	109.6	C5G—C6G—C7F	120.77 (19)
C8B—C9B—H9B1	109.6	C1G—C6G—C7F	122.4 (2)
C10B—C9B—H9B2	109.6	C6H—C7G—C8G	113.25 (19)
C8B—C9B—H9B2	109.6	C6H—C7G—C2G	109.47 (17)
H9B1—C9B—H9B2	108.1	C8G—C7G—C2G	111.85 (18)
C9B—C10B—C11B	115.4 (2)	C6H—C7G—H7G	107.3
C9B—C10B—H10C	108.4	C8G—C7G—H7G	107.3
C11B—C10B—H10C	108.4	C2G—C7G—H7G	107.3
C9B—C10B—H10D	108.4	C9G—C8G—C7G	115.79 (19)
C11B—C10B—H10D	108.4	C9G—C8G—H8G1	108.3
H10C—C10B—H10D	107.5	C7G—C8G—H8G1	108.3
C12B—C11B—C10B	112.5 (2)	C9G—C8G—H8G2	108.3
C12B—C11B—H11C	109.1	C7G—C8G—H8G2	108.3
C10B—C11B—H11C	109.1	H8G1—C8G—H8G2	107.4
C12B—C11B—H11D	109.1	C10G—C9G—C8G	111.05 (19)
C10B—C11B—H11D	109.1	C10G—C9G—H9G1	109.4
H11C—C11B—H11D	107.8	C8G—C9G—H9G1	109.4
C11B—C12B—C13B	113.0 (2)	C10G—C9G—H9G2	109.4
C11B—C12B—H12C	109.0	C8G—C9G—H9G2	109.4
C13B—C12B—H12C	109.0	H9G1—C9G—H9G2	108.0
C11B—C12B—H12D	109.0	C9G—C10G—C11G	115.4 (2)
C13B—C12B—H12D	109.0	C9G—C10G—H10M	108.4
H12C—C12B—H12D	107.8	C11G—C10G—H10M	108.4
C12B—C13B—H13D	109.5	C9G—C10G—H10N	108.4
C12B—C13B—H13E	109.5	C11G—C10G—H10N	108.4
H13D—C13B—H13E	109.5	H10M—C10G—H10N	107.5
C12B—C13B—H13F	109.5	C12G—C11G—C10G	113.3 (2)
H13D—C13B—H13F	109.5	C12G—C11G—H11M	108.9
H13E—C13B—H13F	109.5	C10G—C11G—H11M	108.9
N1B—C14B—O1B	119.4 (2)	C12G—C11G—H11N	108.9
N1B—C14B—C15B	122.3 (2)	C10G—C11G—H11N	108.9
O1B—C14B—C15B	118.4 (2)	H11M—C11G—H11N	107.7
N2B—C15B—O2B	120.6 (2)	C11G—C12G—C13G	113.7 (3)
N2B—C15B—C14B	123.2 (2)	C11G—C12G—H12M	108.8
O2B—C15B—C14B	116.2 (2)	C13G—C12G—H12M	108.8
N2B—C16B—C21B	120.0 (2)	C11G—C12G—H12N	108.8
N2B—C16B—C17B	120.7 (2)	C13G—C12G—H12N	108.8
C21B—C16B—C17B	119.3 (2)	H12M—C12G—H12N	107.7
N1B—C17B—C18B	119.1 (2)	C12G—C13G—H13S	109.5
N1B—C17B—C16B	121.2 (2)	C12G—C13G—H13T	109.5
C18B—C17B—C16B	119.6 (2)	H13S—C13G—H13T	109.5
C19B—C18B—C17B	119.6 (3)	C12G—C13G—H13U	109.5
C19B—C18B—H18B	120.2	H13S—C13G—H13U	109.5
C17B—C18B—H18B	120.2	H13T—C13G—H13U	109.5
C18B—C19B—C20B	120.7 (3)	N1G—C14G—O1G	119.8 (2)
C18B—C19B—H19B	119.7	N1G—C14G—C15G	122.8 (2)
C20B—C19B—H19B	119.7	O1G—C14G—C15G	117.4 (2)
C21B—C20B—C19B	120.7 (3)	N2G—C15G—O2G	119.6 (2)
C21B—C20B—H20B	119.6	N2G—C15G—C14G	122.3 (2)
C19B—C20B—H20B	119.6	O2G—C15G—C14G	118.0 (2)
C20B—C21B—C16B	120.0 (3)	N2G—C16G—C21G	119.6 (2)
C20B—C21B—H21B	120.0	N2G—C16G—C17G	120.7 (2)
C16B—C21B—H21B	120.0	C21G—C16G—C17G	119.6 (2)
C6C—C1C—C2C	123.3 (2)	N1G—C17G—C18G	120.3 (2)
C6C—C1C—H1C	118.3	N1G—C17G—C16G	120.8 (2)
C2C—C1C—H1C	118.3	C18G—C17G—C16G	118.9 (2)
C3C—C2C—C1C	116.2 (2)	C19G—C18G—C17G	120.4 (2)
C3C—C2C—C7C	120.92 (19)	C19G—C18G—H18G	119.8
C1C—C2C—C7C	122.8 (2)	C17G—C18G—H18G	119.8
C4C—C3C—C2C	122.9 (2)	C18G—C19G—C20G	120.7 (3)
C4C—C3C—O1C	119.5 (2)	C18G—C19G—H19G	119.7
C2C—C3C—O1C	117.5 (2)	C20G—C19G—H19G	119.7
C3C—C4C—C5C	118.2 (2)	C21G—C20G—C19G	120.6 (3)
C3C—C4C—H4C	120.9	C21G—C20G—H20G	119.7
C5C—C4C—H4C	120.9	C19G—C20G—H20G	119.7
C4C—C5C—C6C	122.4 (2)	C20G—C21G—C16G	119.7 (2)
C4C—C5C—O2B	119.5 (2)	C20G—C21G—H21G	120.2
C6C—C5C—O2B	118.03 (19)	C16G—C21G—H21G	120.2
C5C—C6C—C1C	116.9 (2)	C6H—C1H—C2H	123.6 (2)
C5C—C6C—C7B	120.5 (2)	C6H—C1H—H1H	118.2
C1C—C6C—C7B	122.5 (2)	C2H—C1H—H1H	118.2
C6D—C7C—C2C	111.16 (18)	C3H—C2H—C1H	116.4 (2)
C6D—C7C—C8C	113.5 (2)	C3H—C2H—C7H	121.72 (19)
C2C—C7C—C8C	110.73 (18)	C1H—C2H—C7H	121.9 (2)
C6D—C7C—H7C	107.0	C4H—C3H—C2H	122.3 (2)
C2C—C7C—H7C	107.0	C4H—C3H—O1H	118.4 (2)
C8C—C7C—H7C	107.0	C2H—C3H—O1H	119.4 (2)
C9C—C8C—C7C	117.3 (2)	C3H—C4H—C5H	119.1 (2)
C9C—C8C—H8C1	108.0	C3H—C4H—H4H	120.5
C7C—C8C—H8C1	108.0	C5H—C4H—H4H	120.5
C9C—C8C—H8C2	108.0	C4H—C5H—C6H	122.1 (2)
C7C—C8C—H8C2	108.0	C4H—C5H—O2G	118.7 (2)
H8C1—C8C—H8C2	107.2	C6H—C5H—O2G	119.24 (19)
C8C—C9C—C10C	107.9 (2)	C5H—C6H—C1H	116.6 (2)
C8C—C9C—H9C1	110.1	C5H—C6H—C7G	121.7 (2)
C10C—C9C—H9C1	110.1	C1H—C6H—C7G	121.7 (2)
C8C—C9C—H9C2	110.1	C2H—C7H—C6E	111.44 (17)
C10C—C9C—H9C2	110.1	C2H—C7H—C8H	113.21 (19)
H9C1—C9C—H9C2	108.4	C6E—C7H—C8H	111.42 (19)
C11C—C10C—C9C	117.1 (3)	C2H—C7H—H7H	106.8
C11C—C10C—H10E	108.0	C6E—C7H—H7H	106.8
C9C—C10C—H10E	108.0	C8H—C7H—H7H	106.8
C11C—C10C—H10F	108.0	C9H—C8H—C7H	115.6 (2)
C9C—C10C—H10F	108.0	C9H—C8H—H8H1	108.4
H10E—C10C—H10F	107.3	C7H—C8H—H8H1	108.4
C10C—C11C—C12C	113.0 (3)	C9H—C8H—H8H2	108.4
C10C—C11C—H11E	109.0	C7H—C8H—H8H2	108.4
C12C—C11C—H11E	109.0	H8H1—C8H—H8H2	107.5
C10C—C11C—H11F	109.0	C10I—C9H—C8H	127.0 (5)
C12C—C11C—H11F	109.0	C8H—C9H—C10H	107.1 (3)
H11E—C11C—H11F	107.8	C8H—C9H—H9HC	110.3
C13C—C12C—C11C	112.6 (3)	C10H—C9H—H9HC	110.3
C13C—C12C—H12E	109.1	C8H—C9H—H9HD	110.3
C11C—C12C—H12E	109.1	C10H—C9H—H9HD	110.3
C13C—C12C—H12F	109.1	H9HC—C9H—H9HD	108.5
C11C—C12C—H12F	109.1	C10I—C9H—H9HA	105.6
H12E—C12C—H12F	107.8	C8H—C9H—H9HA	105.6
C12C—C13C—H13G	109.5	C10I—C9H—H9HB	105.6
C12C—C13C—H13H	109.5	C8H—C9H—H9HB	105.6
H13G—C13C—H13H	109.5	H9HA—C9H—H9HB	106.1
C12C—C13C—H13I	109.5	C11H—C10H—C9H	115.8 (4)
H13G—C13C—H13I	109.5	C11H—C10H—H10O	108.3
H13H—C13C—H13I	109.5	C9H—C10H—H10O	108.3
N1C—C14C—O1C	120.5 (2)	C11H—C10H—H10P	108.3
N1C—C14C—C15C	123.1 (2)	C9H—C10H—H10P	108.3
O1C—C14C—C15C	116.4 (2)	H10O—C10H—H10P	107.4
N2C—C15C—O2C	120.0 (2)	C12H—C11H—C10H	117.0 (4)
N2C—C15C—C14C	122.4 (2)	C12H—C11H—H11O	108.0
O2C—C15C—C14C	117.5 (2)	C10H—C11H—H11O	108.0
N2C—C16C—C21C	119.9 (2)	C12H—C11H—H11P	108.0
N2C—C16C—C17C	120.9 (2)	C10H—C11H—H11P	108.0
C21C—C16C—C17C	119.3 (2)	H11O—C11H—H11P	107.3
N1C—C17C—C18C	119.7 (2)	C13H—C12H—C11H	116.6 (6)
N1C—C17C—C16C	120.9 (2)	C13H—C12H—H12O	108.1
C18C—C17C—C16C	119.4 (2)	C11H—C12H—H12O	108.1
C19C—C18C—C17C	120.2 (3)	C13H—C12H—H12P	108.1
C19C—C18C—H18C	119.9	C11H—C12H—H12P	108.1
C17C—C18C—H18C	119.9	H12O—C12H—H12P	107.3
C18C—C19C—C20C	120.0 (3)	C12H—C13H—H13V	109.5
C18C—C19C—H19C	120.0	C12H—C13H—H13W	109.5
C20C—C19C—H19C	120.0	H13V—C13H—H13W	109.5
C21C—C20C—C19C	121.1 (3)	C12H—C13H—H13$	109.5
C21C—C20C—H20C	119.5	H13V—C13H—H13$	109.5
C19C—C20C—H20C	119.5	H13W—C13H—H13$	109.5
C20C—C21C—C16C	120.1 (3)	C9H—C10I—C11I	106.9 (8)
C20C—C21C—H21C	120.0	C9H—C10I—H10Q	110.3
C16C—C21C—H21C	120.0	C11I—C10I—H10Q	110.3
C6D—C1D—C2D	123.4 (2)	C9H—C10I—H10R	110.3
C6D—C1D—H1D	118.3	C11I—C10I—H10R	110.3
C2D—C1D—H1D	118.3	H10Q—C10I—H10R	108.6
C3D—C2D—C1D	116.8 (2)	C12I—C11I—C10I	114.9 (11)
C3D—C2D—C7D	121.5 (2)	C12I—C11I—H11Q	108.5
C1D—C2D—C7D	121.57 (19)	C10I—C11I—H11Q	108.5
C4D—C3D—C2D	121.8 (2)	C12I—C11I—H11R	108.5
C4D—C3D—O1D	119.10 (19)	C10I—C11I—H11R	108.5
C2D—C3D—O1D	119.06 (19)	H11Q—C11I—H11R	107.5
C5D—C4D—C3D	119.1 (2)	C11I—C12I—C13I	117.3 (10)
C5D—C4D—H4D	120.5	C11I—C12I—H12Q	108.0
C3D—C4D—H4D	120.5	C13I—C12I—H12Q	108.0
C4D—C5D—C6D	122.2 (2)	C11I—C12I—H12R	108.0
C4D—C5D—O2C	117.64 (19)	C13I—C12I—H12R	108.0
C6D—C5D—O2C	120.1 (2)	H12Q—C12I—H12R	107.2
C5D—C6D—C1D	116.6 (2)	C12I—C13I—H13Z	109.5
C5D—C6D—C7C	122.56 (19)	C12I—C13I—H131	109.5
C1D—C6D—C7C	120.8 (2)	H13Z—C13I—H131	109.5
C2D—C7D—C6A	108.95 (17)	C12I—C13I—H132	109.5
C2D—C7D—C8D	113.03 (18)	H13Z—C13I—H132	109.5
C6A—C7D—C8D	111.69 (17)	H131—C13I—H132	109.5
C2D—C7D—H7D	107.6	N1H—C14H—O1H	119.5 (2)
C6A—C7D—H7D	107.6	N1H—C14H—C15H	122.4 (2)
C8D—C7D—H7D	107.6	O1H—C14H—C15H	118.1 (2)
C9D—C8D—C7D	115.47 (18)	N2H—C15H—O2H	120.4 (2)
C9D—C8D—H8D1	108.4	N2H—C15H—C14H	122.6 (2)
C7D—C8D—H8D1	108.4	O2H—C15H—C14H	117.0 (2)
C9D—C8D—H8D2	108.4	N2H—C16H—C21H	119.8 (2)
C7D—C8D—H8D2	108.4	N2H—C16H—C17H	121.2 (2)
H8D1—C8D—H8D2	107.5	C21H—C16H—C17H	119.0 (3)
C10D—C9D—C8D	109.84 (19)	N1H—C17H—C18H	119.8 (2)
C10D—C9D—H9D1	109.7	N1H—C17H—C16H	120.4 (2)
C8D—C9D—H9D1	109.7	C18H—C17H—C16H	119.8 (2)
C10D—C9D—H9D2	109.7	C19H—C18H—C17H	120.0 (3)
C8D—C9D—H9D2	109.7	C19H—C18H—H18H	120.0
H9D1—C9D—H9D2	108.2	C17H—C18H—H18H	120.0
C11D—C10D—C9D	115.5 (2)	C18H—C19H—C20H	120.3 (3)
C11D—C10D—H10G	108.4	C18H—C19H—H19H	119.9
C9D—C10D—H10G	108.4	C20H—C19H—H19H	119.9
C11D—C10D—H10H	108.4	C21H—C20H—C19H	121.2 (3)
C9D—C10D—H10H	108.4	C21H—C20H—H20H	119.4
H10G—C10D—H10H	107.5	C19H—C20H—H20H	119.4
C10D—C11D—C12D	112.7 (2)	C20H—C21H—C16H	119.8 (3)
C10D—C11D—H11G	109.1	C20H—C21H—H21H	120.1
C12D—C11D—H11G	109.1	C16H—C21H—H21H	120.1
C10D—C11D—H11H	109.1	C2R—C1R—C6R	120.1 (3)
C12D—C11D—H11H	109.1	C2R—C1R—H1R	120.0
H11G—C11D—H11H	107.8	C6R—C1R—H1R	120.0
C13D—C12D—C11D	113.4 (2)	C3R—C2R—C1R	120.2 (3)
C13D—C12D—H12G	108.9	C3R—C2R—H2R	119.9
C11D—C12D—H12G	108.9	C1R—C2R—H2R	119.9
C13D—C12D—H12H	108.9	C2R—C3R—C4R	119.7 (2)
C11D—C12D—H12H	108.9	C2R—C3R—H3R	120.2
H12G—C12D—H12H	107.7	C4R—C3R—H3R	120.2
C12D—C13D—H13J	109.5	C5R—C4R—C3R	119.7 (3)
C12D—C13D—H13K	109.5	C5R—C4R—C7R	119.8 (3)
H13J—C13D—H13K	109.5	C3R—C4R—C7R	120.5 (3)
C12D—C13D—H13L	109.5	C6R—C5R—C4R	120.0 (3)
H13J—C13D—H13L	109.5	C6R—C5R—H5R	120.0
H13K—C13D—H13L	109.5	C4R—C5R—H5R	120.0
N1D—C14D—O1D	119.8 (2)	C5R—C6R—C1R	120.4 (3)
N1D—C14D—C15D	122.7 (2)	C5R—C6R—H6R	119.8
O1D—C14D—C15D	117.5 (2)	C1R—C6R—H6R	119.8
N2D—C15D—O2D	120.0 (2)	N1R—C7R—C4R	178.4 (4)
N2D—C15D—C14D	122.6 (2)	C6S—C1S—C2S	120.5 (4)
O2D—C15D—C14D	117.4 (2)	C6S—C1S—H1S	119.7
N2D—C16D—C17D	121.6 (2)	C2S—C1S—H1S	119.7
N2D—C16D—C21D	119.3 (2)	C3S—C2S—C1S	120.1 (3)
C17D—C16D—C21D	119.1 (2)	C3S—C2S—H2S	119.9
N1D—C17D—C18D	119.6 (2)	C1S—C2S—H2S	119.9
N1D—C17D—C16D	120.5 (2)	C2S—C3S—C4S	119.4 (3)
C18D—C17D—C16D	119.8 (2)	C2S—C3S—H3S	120.3
C19D—C18D—C17D	119.6 (3)	C4S—C3S—H3S	120.3
C19D—C18D—H18D	120.2	C3S—C4S—C5S	120.6 (3)
C17D—C18D—H18D	120.2	C3S—C4S—C7S	119.5 (3)
C18D—C19D—C20D	120.9 (3)	C5S—C4S—C7S	119.9 (3)
C18D—C19D—H19D	119.6	C6S—C5S—C4S	118.8 (3)
C20D—C19D—H19D	119.6	C6S—C5S—H5S	120.6
C21D—C20D—C19D	120.4 (3)	C4S—C5S—H5S	120.6
C21D—C20D—H20D	119.8	C1S—C6S—C5S	120.6 (3)
C19D—C20D—H20D	119.8	C1S—C6S—H6S	119.7
C20D—C21D—C16D	120.1 (3)	C5S—C6S—H6S	119.7
C20D—C21D—H21D	119.9	N1S—C7S—C4S	179.2 (3)
C16D—C21D—H21D	119.9	C2T—C1T—C6T	119.6 (3)
C6E—C1E—C2E	123.1 (2)	C2T—C1T—H1T	120.2
C6E—C1E—H1E	118.5	C6T—C1T—H1T	120.2
C2E—C1E—H1E	118.5	C3T—C2T—C1T	120.8 (3)
C3E—C2E—C1E	116.6 (2)	C3T—C2T—H2T	119.6
C3E—C2E—C7E	121.6 (2)	C1T—C2T—H2T	119.6
C1E—C2E—C7E	121.8 (2)	C2T—C3T—C4T	119.9 (3)
C2E—C3E—C4E	122.8 (2)	C2T—C3T—H3T	120.0
C2E—C3E—O1E	118.5 (2)	C4T—C3T—H3T	120.0
C4E—C3E—O1E	118.8 (2)	C3T—C4T—C5T	120.0 (3)
C5E—C4E—C3E	118.1 (2)	C3T—C4T—C7T	119.5 (3)
C5E—C4E—H4E	120.9	C5T—C4T—C7T	120.4 (3)
C3E—C4E—H4E	120.9	C6T—C5T—C4T	119.6 (3)
C4E—C5E—C6E	122.5 (2)	C6T—C5T—H5T	120.2
C4E—C5E—O2H	119.3 (2)	C4T—C5T—H5T	120.2
C6E—C5E—O2H	118.2 (2)	C5T—C6T—C1T	120.0 (3)
C5E—C6E—C1E	116.8 (2)	C5T—C6T—H6T	120.0
C5E—C6E—C7H	120.8 (2)	C1T—C6T—H6T	120.0
C1E—C6E—C7H	122.3 (2)	N1T—C7T—C4T	178.6 (4)
C6F—C7E—C2E	108.89 (18)	C2U—C1U—C6U	121.5 (4)
C6F—C7E—C8E	112.44 (18)	C2U—C1U—H1U	119.2
C2E—C7E—C8E	111.44 (18)	C6U—C1U—H1U	119.2
C6F—C7E—H7E	108.0	C1U—C2U—C3U	120.2 (4)
C2E—C7E—H7E	108.0	C1U—C2U—H2U	119.9
C8E—C7E—H7E	108.0	C3U—C2U—H2U	119.9
C9E—C8E—C7E	115.94 (18)	C2U—C3U—C4U	119.4 (3)
C9E—C8E—H8E1	108.3	C2U—C3U—H3U	120.3
C7E—C8E—H8E1	108.3	C4U—C3U—H3U	120.3
C9E—C8E—H8E2	108.3	C5U—C4U—C3U	120.9 (3)
C7E—C8E—H8E2	108.3	C5U—C4U—C7U	120.7 (3)
H8E1—C8E—H8E2	107.4	C3U—C4U—C7U	118.4 (3)
C10E—C9E—C8E	109.30 (18)	C4U—C5U—C6U	118.3 (3)
C10E—C9E—H9E1	109.8	C4U—C5U—H5U	120.9
C8E—C9E—H9E1	109.8	C6U—C5U—H5U	120.9
C10E—C9E—H9E2	109.8	C1U—C6U—C5U	119.8 (3)
C8E—C9E—H9E2	109.8	C1U—C6U—H6U	120.1
H9E1—C9E—H9E2	108.3	C5U—C6U—H6U	120.1
C11E—C10E—C9E	117.1 (2)	N1U—C7U—C4U	179.2 (3)
			
C6A—C1A—C2A—C3A	2.5 (3)	C2E—C1E—C6E—C5E	−1.9 (3)
C6A—C1A—C2A—C7A	−174.3 (2)	C2E—C1E—C6E—C7H	175.0 (2)
C1A—C2A—C3A—C4A	−3.0 (3)	C3E—C2E—C7E—C6F	89.8 (2)
C7A—C2A—C3A—C4A	173.8 (2)	C1E—C2E—C7E—C6F	−90.3 (2)
C1A—C2A—C3A—O1A	−178.40 (18)	C3E—C2E—C7E—C8E	−145.6 (2)
C7A—C2A—C3A—O1A	−1.6 (3)	C1E—C2E—C7E—C8E	34.3 (3)
C14A—O1A—C3A—C4A	78.8 (3)	C6F—C7E—C8E—C9E	−60.6 (2)
C14A—O1A—C3A—C2A	−105.6 (2)	C2E—C7E—C8E—C9E	176.86 (18)
C2A—C3A—C4A—C5A	1.0 (3)	C7E—C8E—C9E—C10E	170.61 (19)
O1A—C3A—C4A—C5A	176.41 (19)	C8E—C9E—C10E—C11E	173.6 (2)
C3A—C4A—C5A—C6A	1.6 (3)	C9E—C10E—C11E—C12E	−179.3 (2)
C3A—C4A—C5A—O2D	−175.74 (19)	C10E—C11E—C12E—C13E	173.9 (3)
C15D—O2D—C5A—C6A	110.9 (2)	C17E—N1E—C14E—O1E	179.2 (2)
C15D—O2D—C5A—C4A	−71.6 (3)	C17E—N1E—C14E—C15E	−1.7 (3)
C4A—C5A—C6A—C1A	−2.1 (3)	C3E—O1E—C14E—N1E	−76.8 (3)
O2D—C5A—C6A—C1A	175.29 (18)	C3E—O1E—C14E—C15E	104.1 (2)
C4A—C5A—C6A—C7D	177.6 (2)	C16E—N2E—C15E—O2E	−178.43 (19)
O2D—C5A—C6A—C7D	−5.0 (3)	C16E—N2E—C15E—C14E	1.0 (3)
C2A—C1A—C6A—C5A	0.0 (3)	C5F—O2E—C15E—N2E	79.5 (2)
C2A—C1A—C6A—C7D	−179.75 (19)	C5F—O2E—C15E—C14E	−99.9 (2)
C3A—C2A—C7A—C6B	91.2 (2)	N1E—C14E—C15E—N2E	0.2 (4)
C1A—C2A—C7A—C6B	−92.2 (2)	O1E—C14E—C15E—N2E	179.4 (2)
C3A—C2A—C7A—C8A	−142.3 (2)	N1E—C14E—C15E—O2E	179.6 (2)
C1A—C2A—C7A—C8A	34.3 (3)	O1E—C14E—C15E—O2E	−1.2 (3)
C6B—C7A—C8A—C9A	−62.4 (3)	C15E—N2E—C16E—C21E	178.6 (2)
C2A—C7A—C8A—C9A	171.6 (2)	C15E—N2E—C16E—C17E	−0.7 (3)
C7A—C8A—C9A—C10A	170.5 (3)	C14E—N1E—C17E—C18E	−177.4 (2)
C8A—C9A—C10A—C11A	60.8 (4)	C14E—N1E—C17E—C16E	1.9 (3)
C9A—C10A—C11A—C12A	169.2 (3)	N2E—C16E—C17E—N1E	−0.8 (4)
C10A—C11A—C12A—C13A	178.9 (4)	C21E—C16E—C17E—N1E	179.9 (2)
C17A—N1A—C14A—O1A	−176.8 (2)	N2E—C16E—C17E—C18E	178.5 (2)
C17A—N1A—C14A—C15A	1.4 (3)	C21E—C16E—C17E—C18E	−0.8 (4)
C3A—O1A—C14A—N1A	−70.9 (3)	N1E—C17E—C18E—C19E	178.3 (3)
C3A—O1A—C14A—C15A	110.8 (2)	C16E—C17E—C18E—C19E	−1.0 (4)
C16A—N2A—C15A—O2A	−178.68 (19)	C17E—C18E—C19E—C20E	1.8 (5)
C16A—N2A—C15A—C14A	1.2 (3)	C18E—C19E—C20E—C21E	−0.9 (5)
C5B—O2A—C15A—N2A	81.5 (2)	C19E—C20E—C21E—C16E	−1.0 (5)
C5B—O2A—C15A—C14A	−98.3 (2)	N2E—C16E—C21E—C20E	−177.5 (3)
N1A—C14A—C15A—N2A	−3.2 (3)	C17E—C16E—C21E—C20E	1.8 (4)
O1A—C14A—C15A—N2A	175.1 (2)	C6F—C1F—C2F—C3F	0.9 (3)
N1A—C14A—C15A—O2A	176.67 (19)	C6F—C1F—C2F—C7F	−178.37 (19)
O1A—C14A—C15A—O2A	−5.1 (3)	C1F—C2F—C3F—C4F	−1.9 (3)
C15A—N2A—C16A—C21A	−180.0 (2)	C7F—C2F—C3F—C4F	177.4 (2)
C15A—N2A—C16A—C17A	2.2 (3)	C1F—C2F—C3F—O1F	179.08 (18)
C14A—N1A—C17A—C18A	−178.1 (2)	C7F—C2F—C3F—O1F	−1.6 (3)
C14A—N1A—C17A—C16A	1.9 (3)	C14F—O1F—C3F—C4F	75.6 (2)
N2A—C16A—C17A—N1A	−3.9 (4)	C14F—O1F—C3F—C2F	−105.4 (2)
C21A—C16A—C17A—N1A	178.2 (2)	C2F—C3F—C4F—C5F	1.7 (3)
N2A—C16A—C17A—C18A	176.2 (2)	O1F—C3F—C4F—C5F	−179.29 (19)
C21A—C16A—C17A—C18A	−1.7 (4)	C3F—C4F—C5F—C6F	−0.4 (3)
N1A—C17A—C18A—C19A	−178.9 (3)	C3F—C4F—C5F—O2E	−179.83 (19)
C16A—C17A—C18A—C19A	1.0 (4)	C15E—O2E—C5F—C4F	−71.7 (2)
C17A—C18A—C19A—C20A	0.0 (5)	C15E—O2E—C5F—C6F	108.9 (2)
C18A—C19A—C20A—C21A	−0.4 (5)	C4F—C5F—C6F—C1F	−0.5 (3)
C19A—C20A—C21A—C16A	−0.4 (5)	O2E—C5F—C6F—C1F	178.86 (18)
N2A—C16A—C21A—C20A	−176.5 (3)	C4F—C5F—C6F—C7E	178.4 (2)
C17A—C16A—C21A—C20A	1.4 (4)	O2E—C5F—C6F—C7E	−2.2 (3)
C6B—C1B—C2B—C3B	−0.3 (3)	C2F—C1F—C6F—C5F	0.3 (3)
C6B—C1B—C2B—C7B	177.53 (19)	C2F—C1F—C6F—C7E	−178.7 (2)
C1B—C2B—C3B—C4B	1.0 (3)	C2E—C7E—C6F—C5F	−93.0 (2)
C7B—C2B—C3B—C4B	−176.83 (19)	C8E—C7E—C6F—C5F	143.0 (2)
C1B—C2B—C3B—O1B	−178.59 (18)	C2E—C7E—C6F—C1F	85.9 (2)
C7B—C2B—C3B—O1B	3.6 (3)	C8E—C7E—C6F—C1F	−38.1 (3)
C14B—O1B—C3B—C4B	70.2 (2)	C3F—C2F—C7F—C6G	96.2 (2)
C14B—O1B—C3B—C2B	−110.2 (2)	C1F—C2F—C7F—C6G	−84.6 (2)
C2B—C3B—C4B—C5B	0.0 (3)	C3F—C2F—C7F—C8F	−136.4 (2)
O1B—C3B—C4B—C5B	179.56 (18)	C1F—C2F—C7F—C8F	42.9 (3)
C3B—C4B—C5B—C6B	−1.7 (3)	C6G—C7F—C8F—C9F	−172.2 (2)
C3B—C4B—C5B—O2A	178.87 (18)	C2F—C7F—C8F—C9F	61.0 (3)
C15A—O2A—C5B—C4B	−75.9 (2)	C7F—C8F—C9F—C10F	158.0 (2)
C15A—O2A—C5B—C6B	104.7 (2)	C8F—C9F—C10F—C11F	60.7 (4)
C4B—C5B—C6B—C1B	2.3 (3)	C9F—C10F—C11F—C12F	−179.6 (3)
O2A—C5B—C6B—C1B	−178.29 (18)	C10F—C11F—C12F—C13F	−64.3 (5)
C4B—C5B—C6B—C7A	−177.15 (19)	C17F—N1F—C14F—O1F	−179.41 (19)
O2A—C5B—C6B—C7A	2.2 (3)	C17F—N1F—C14F—C15F	−0.3 (3)
C2B—C1B—C6B—C5B	−1.3 (3)	C3F—O1F—C14F—N1F	−81.8 (2)
C2B—C1B—C6B—C7A	178.17 (19)	C3F—O1F—C14F—C15F	99.1 (2)
C2A—C7A—C6B—C5B	−96.6 (2)	C16F—N2F—C15F—O2F	175.85 (19)
C8A—C7A—C6B—C5B	136.6 (2)	C16F—N2F—C15F—C14F	−1.0 (3)
C2A—C7A—C6B—C1B	83.9 (3)	C5G—O2F—C15F—N2F	73.5 (3)
C8A—C7A—C6B—C1B	−42.8 (3)	C5G—O2F—C15F—C14F	−109.5 (2)
C3B—C2B—C7B—C8B	−140.6 (2)	N1F—C14F—C15F—N2F	2.4 (3)
C1B—C2B—C7B—C8B	41.7 (3)	O1F—C14F—C15F—N2F	−178.53 (19)
C3B—C2B—C7B—C6C	95.0 (2)	N1F—C14F—C15F—O2F	−174.59 (19)
C1B—C2B—C7B—C6C	−82.7 (3)	O1F—C14F—C15F—O2F	4.5 (3)
C2B—C7B—C8B—C9B	57.7 (3)	C15F—N2F—C16F—C21F	179.6 (2)
C6C—C7B—C8B—C9B	−179.1 (2)	C15F—N2F—C16F—C17F	−2.1 (3)
C7B—C8B—C9B—C10B	−168.5 (2)	C14F—N1F—C17F—C18F	178.1 (2)
C8B—C9B—C10B—C11B	−179.1 (2)	C14F—N1F—C17F—C16F	−2.7 (3)
C9B—C10B—C11B—C12B	−174.3 (2)	N2F—C16F—C17F—N1F	4.2 (3)
C10B—C11B—C12B—C13B	179.2 (3)	C21F—C16F—C17F—N1F	−177.5 (2)
C17B—N1B—C14B—O1B	176.6 (2)	N2F—C16F—C17F—C18F	−176.7 (2)
C17B—N1B—C14B—C15B	−2.9 (3)	C21F—C16F—C17F—C18F	1.6 (4)
C3B—O1B—C14B—N1B	−86.4 (2)	N1F—C17F—C18F—C19F	178.0 (2)
C3B—O1B—C14B—C15B	93.1 (2)	C16F—C17F—C18F—C19F	−1.1 (4)
C16B—N2B—C15B—O2B	176.7 (2)	C17F—C18F—C19F—C20F	−0.1 (4)
C16B—N2B—C15B—C14B	−1.4 (3)	C18F—C19F—C20F—C21F	0.9 (5)
C5C—O2B—C15B—N2B	71.5 (3)	C19F—C20F—C21F—C16F	−0.4 (4)
C5C—O2B—C15B—C14B	−110.3 (2)	N2F—C16F—C21F—C20F	177.5 (2)
N1B—C14B—C15B—N2B	4.7 (4)	C17F—C16F—C21F—C20F	−0.8 (4)
O1B—C14B—C15B—N2B	−174.9 (2)	C6G—C1G—C2G—C3G	0.5 (3)
N1B—C14B—C15B—O2B	−173.5 (2)	C6G—C1G—C2G—C7G	−179.7 (2)
O1B—C14B—C15B—O2B	7.0 (3)	C1G—C2G—C3G—C4G	0.9 (3)
C15B—N2B—C16B—C21B	177.4 (2)	C7G—C2G—C3G—C4G	−178.9 (2)
C15B—N2B—C16B—C17B	−3.0 (3)	C1G—C2G—C3G—O1G	−176.22 (18)
C14B—N1B—C17B—C18B	−179.7 (2)	C7G—C2G—C3G—O1G	3.9 (3)
C14B—N1B—C17B—C16B	−1.5 (3)	C14G—O1G—C3G—C2G	−109.8 (2)
N2B—C16B—C17B—N1B	4.7 (4)	C14G—O1G—C3G—C4G	72.9 (3)
C21B—C16B—C17B—N1B	−175.7 (2)	C2G—C3G—C4G—C5G	−0.6 (3)
N2B—C16B—C17B—C18B	−177.2 (2)	O1G—C3G—C4G—C5G	176.5 (2)
C21B—C16B—C17B—C18B	2.5 (4)	C3G—C4G—C5G—C6G	−1.2 (3)
N1B—C17B—C18B—C19B	176.0 (3)	C3G—C4G—C5G—O2F	−178.1 (2)
C16B—C17B—C18B—C19B	−2.2 (4)	C15F—O2F—C5G—C4G	−77.1 (3)
C17B—C18B—C19B—C20B	0.6 (5)	C15F—O2F—C5G—C6G	105.9 (2)
C18B—C19B—C20B—C21B	0.7 (5)	C4G—C5G—C6G—C1G	2.5 (3)
C19B—C20B—C21B—C16B	−0.5 (4)	O2F—C5G—C6G—C1G	179.36 (19)
N2B—C16B—C21B—C20B	178.5 (2)	C4G—C5G—C6G—C7F	−175.7 (2)
C17B—C16B—C21B—C20B	−1.1 (4)	O2F—C5G—C6G—C7F	1.2 (3)
C6C—C1C—C2C—C3C	1.8 (3)	C2G—C1G—C6G—C5G	−2.1 (3)
C6C—C1C—C2C—C7C	−177.2 (2)	C2G—C1G—C6G—C7F	176.0 (2)
C1C—C2C—C3C—C4C	−1.1 (3)	C2F—C7F—C6G—C5G	−91.4 (2)
C7C—C2C—C3C—C4C	177.9 (2)	C8F—C7F—C6G—C5G	141.1 (2)
C1C—C2C—C3C—O1C	−177.78 (19)	C2F—C7F—C6G—C1G	90.5 (2)
C7C—C2C—C3C—O1C	1.3 (3)	C8F—C7F—C6G—C1G	−36.9 (3)
C14C—O1C—C3C—C4C	73.5 (3)	C3G—C2G—C7G—C6H	89.4 (2)
C14C—O1C—C3C—C2C	−109.8 (2)	C1G—C2G—C7G—C6H	−90.4 (2)
C2C—C3C—C4C—C5C	−0.2 (3)	C3G—C2G—C7G—C8G	−144.2 (2)
O1C—C3C—C4C—C5C	176.4 (2)	C1G—C2G—C7G—C8G	36.0 (3)
C3C—C4C—C5C—C6C	0.9 (3)	C6H—C7G—C8G—C9G	−61.3 (3)
C3C—C4C—C5C—O2B	−175.1 (2)	C2G—C7G—C8G—C9G	174.43 (19)
C15B—O2B—C5C—C4C	−72.3 (3)	C7G—C8G—C9G—C10G	178.8 (2)
C15B—O2B—C5C—C6C	111.5 (2)	C8G—C9G—C10G—C11G	178.0 (2)
C4C—C5C—C6C—C1C	−0.2 (3)	C9G—C10G—C11G—C12G	175.6 (2)
O2B—C5C—C6C—C1C	175.84 (19)	C10G—C11G—C12G—C13G	−179.6 (3)
C4C—C5C—C6C—C7B	178.9 (2)	C17G—N1G—C14G—O1G	−177.5 (2)
O2B—C5C—C6C—C7B	−5.1 (3)	C17G—N1G—C14G—C15G	−0.1 (3)
C2C—C1C—C6C—C5C	−1.2 (3)	C3G—O1G—C14G—N1G	−74.0 (3)
C2C—C1C—C6C—C7B	179.7 (2)	C3G—O1G—C14G—C15G	108.5 (2)
C2B—C7B—C6C—C5C	−86.6 (3)	C16G—N2G—C15G—O2G	−176.60 (19)
C8B—C7B—C6C—C5C	148.0 (2)	C16G—N2G—C15G—C14G	3.7 (3)
C2B—C7B—C6C—C1C	92.4 (3)	C5H—O2G—C15G—N2G	85.6 (2)
C8B—C7B—C6C—C1C	−33.0 (3)	C5H—O2G—C15G—C14G	−94.7 (2)
C3C—C2C—C7C—C6D	88.3 (2)	N1G—C14G—C15G—N2G	−4.2 (4)
C1C—C2C—C7C—C6D	−92.7 (2)	O1G—C14G—C15G—N2G	173.2 (2)
C3C—C2C—C7C—C8C	−144.5 (2)	N1G—C14G—C15G—O2G	176.1 (2)
C1C—C2C—C7C—C8C	34.5 (3)	O1G—C14G—C15G—O2G	−6.5 (3)
C6D—C7C—C8C—C9C	−55.9 (3)	C15G—N2G—C16G—C21G	178.0 (2)
C2C—C7C—C8C—C9C	178.2 (2)	C15G—N2G—C16G—C17G	0.5 (3)
C7C—C8C—C9C—C10C	−174.2 (2)	C14G—N1G—C17G—C18G	−174.7 (2)
C8C—C9C—C10C—C11C	−169.9 (3)	C14G—N1G—C17G—C16G	4.3 (3)
C9C—C10C—C11C—C12C	178.2 (3)	N2G—C16G—C17G—N1G	−4.7 (3)
C10C—C11C—C12C—C13C	178.0 (3)	C21G—C16G—C17G—N1G	177.8 (2)
C17C—N1C—C14C—O1C	−175.67 (19)	N2G—C16G—C17G—C18G	174.3 (2)
C17C—N1C—C14C—C15C	1.4 (3)	C21G—C16G—C17G—C18G	−3.2 (4)
C3C—O1C—C14C—N1C	−71.5 (3)	N1G—C17G—C18G—C19G	180.0 (2)
C3C—O1C—C14C—C15C	111.2 (2)	C16G—C17G—C18G—C19G	0.9 (4)
C16C—N2C—C15C—O2C	−179.62 (18)	C17G—C18G—C19G—C20G	1.9 (4)
C16C—N2C—C15C—C14C	1.2 (3)	C18G—C19G—C20G—C21G	−2.5 (4)
C5D—O2C—C15C—N2C	84.1 (2)	C19G—C20G—C21G—C16G	0.2 (4)
C5D—O2C—C15C—C14C	−96.6 (2)	N2G—C16G—C21G—C20G	−174.9 (2)
N1C—C14C—C15C—N2C	−2.9 (3)	C17G—C16G—C21G—C20G	2.6 (4)
O1C—C14C—C15C—N2C	174.28 (19)	C6H—C1H—C2H—C3H	1.7 (3)
N1C—C14C—C15C—O2C	177.85 (19)	C6H—C1H—C2H—C7H	−178.24 (19)
O1C—C14C—C15C—O2C	−4.9 (3)	C1H—C2H—C3H—C4H	−2.4 (3)
C15C—N2C—C16C—C21C	−178.4 (2)	C7H—C2H—C3H—C4H	177.46 (19)
C15C—N2C—C16C—C17C	1.6 (3)	C1H—C2H—C3H—O1H	178.31 (18)
C14C—N1C—C17C—C18C	179.9 (2)	C7H—C2H—C3H—O1H	−1.8 (3)
C14C—N1C—C17C—C16C	1.4 (3)	C14H—O1H—C3H—C4H	76.0 (2)
N2C—C16C—C17C—N1C	−3.1 (3)	C14H—O1H—C3H—C2H	−104.7 (2)
C21C—C16C—C17C—N1C	177.0 (2)	C2H—C3H—C4H—C5H	1.4 (3)
N2C—C16C—C17C—C18C	178.5 (2)	O1H—C3H—C4H—C5H	−179.38 (18)
C21C—C16C—C17C—C18C	−1.5 (4)	C3H—C4H—C5H—C6H	0.7 (3)
N1C—C17C—C18C—C19C	−177.5 (3)	C3H—C4H—C5H—O2G	−178.17 (18)
C16C—C17C—C18C—C19C	1.0 (4)	C15G—O2G—C5H—C4H	−71.1 (2)
C17C—C18C—C19C—C20C	0.1 (4)	C15G—O2G—C5H—C6H	110.1 (2)
C18C—C19C—C20C—C21C	−0.7 (5)	C4H—C5H—C6H—C1H	−1.4 (3)
C19C—C20C—C21C—C16C	0.1 (4)	O2G—C5H—C6H—C1H	177.44 (18)
N2C—C16C—C21C—C20C	−179.0 (2)	C4H—C5H—C6H—C7G	176.9 (2)
C17C—C16C—C21C—C20C	0.9 (4)	O2G—C5H—C6H—C7G	−4.3 (3)
C6D—C1D—C2D—C3D	0.2 (3)	C2H—C1H—C6H—C5H	0.2 (3)
C6D—C1D—C2D—C7D	177.8 (2)	C2H—C1H—C6H—C7G	−178.12 (19)
C1D—C2D—C3D—C4D	1.1 (3)	C8G—C7G—C6H—C5H	140.1 (2)
C7D—C2D—C3D—C4D	−176.4 (2)	C2G—C7G—C6H—C5H	−94.3 (2)
C1D—C2D—C3D—O1D	−177.74 (18)	C8G—C7G—C6H—C1H	−41.7 (3)
C7D—C2D—C3D—O1D	4.7 (3)	C2G—C7G—C6H—C1H	83.9 (3)
C14D—O1D—C3D—C4D	69.9 (2)	C3H—C2H—C7H—C6E	96.0 (2)
C14D—O1D—C3D—C2D	−111.2 (2)	C1H—C2H—C7H—C6E	−84.1 (3)
C2D—C3D—C4D—C5D	−0.9 (3)	C3H—C2H—C7H—C8H	−137.5 (2)
O1D—C3D—C4D—C5D	178.00 (19)	C1H—C2H—C7H—C8H	42.4 (3)
C3D—C4D—C5D—C6D	−0.8 (3)	C5E—C6E—C7H—C2H	−92.3 (2)
C3D—C4D—C5D—O2C	−179.12 (19)	C1E—C6E—C7H—C2H	90.9 (3)
C15C—O2C—C5D—C4D	−75.3 (2)	C5E—C6E—C7H—C8H	140.2 (2)
C15C—O2C—C5D—C6D	106.3 (2)	C1E—C6E—C7H—C8H	−36.6 (3)
C4D—C5D—C6D—C1D	2.1 (3)	C2H—C7H—C8H—C9H	58.1 (3)
O2C—C5D—C6D—C1D	−179.64 (19)	C6E—C7H—C8H—C9H	−175.4 (2)
C4D—C5D—C6D—C7C	−177.9 (2)	C7H—C8H—C9H—C10I	153.5 (7)
O2C—C5D—C6D—C7C	0.3 (3)	C7H—C8H—C9H—C10H	172.7 (3)
C2D—C1D—C6D—C5D	−1.8 (3)	C8H—C9H—C10H—C11H	172.6 (4)
C2D—C1D—C6D—C7C	178.2 (2)	C9H—C10H—C11H—C12H	168.8 (5)
C2C—C7C—C6D—C5D	−94.7 (2)	C10H—C11H—C12H—C13H	57.6 (9)
C8C—C7C—C6D—C5D	139.6 (2)	C8H—C9H—C10I—C11I	70.1 (10)
C2C—C7C—C6D—C1D	85.2 (3)	C9H—C10I—C11I—C12I	−179.6 (8)
C8C—C7C—C6D—C1D	−40.4 (3)	C10I—C11I—C12I—C13I	−73.6 (18)
C3D—C2D—C7D—C6A	92.7 (2)	C17H—N1H—C14H—O1H	178.20 (19)
C1D—C2D—C7D—C6A	−84.7 (3)	C17H—N1H—C14H—C15H	−1.2 (3)
C3D—C2D—C7D—C8D	−142.5 (2)	C3H—O1H—C14H—N1H	−80.6 (3)
C1D—C2D—C7D—C8D	40.1 (3)	C3H—O1H—C14H—C15H	98.8 (2)
C5A—C6A—C7D—C2D	−89.4 (2)	C16H—N2H—C15H—O2H	177.0 (2)
C1A—C6A—C7D—C2D	90.3 (2)	C16H—N2H—C15H—C14H	−1.5 (3)
C5A—C6A—C7D—C8D	145.0 (2)	C5E—O2H—C15H—N2H	71.1 (3)
C1A—C6A—C7D—C8D	−35.3 (3)	C5E—O2H—C15H—C14H	−110.3 (2)
C2D—C7D—C8D—C9D	66.0 (3)	N1H—C14H—C15H—N2H	3.0 (3)
C6A—C7D—C8D—C9D	−170.72 (19)	O1H—C14H—C15H—N2H	−176.4 (2)
C7D—C8D—C9D—C10D	−178.2 (2)	N1H—C14H—C15H—O2H	−175.49 (19)
C8D—C9D—C10D—C11D	−171.1 (2)	O1H—C14H—C15H—O2H	5.1 (3)
C9D—C10D—C11D—C12D	−178.2 (2)	C15H—N2H—C16H—C21H	178.7 (2)
C10D—C11D—C12D—C13D	−175.4 (2)	C15H—N2H—C16H—C17H	−1.6 (3)
C17D—N1D—C14D—O1D	177.47 (19)	C14H—N1H—C17H—C18H	179.4 (2)
C17D—N1D—C14D—C15D	−2.0 (3)	C14H—N1H—C17H—C16H	−1.8 (3)
C3D—O1D—C14D—N1D	−81.4 (2)	N2H—C16H—C17H—N1H	3.3 (4)
C3D—O1D—C14D—C15D	98.1 (2)	C21H—C16H—C17H—N1H	−176.9 (2)
C16D—N2D—C15D—O2D	179.8 (2)	N2H—C16H—C17H—C18H	−177.9 (2)
C16D—N2D—C15D—C14D	1.9 (3)	C21H—C16H—C17H—C18H	1.9 (4)
C5A—O2D—C15D—N2D	76.9 (3)	N1H—C17H—C18H—C19H	178.0 (2)
C5A—O2D—C15D—C14D	−105.1 (2)	C16H—C17H—C18H—C19H	−0.8 (4)
N1D—C14D—C15D—N2D	0.7 (4)	C17H—C18H—C19H—C20H	−0.4 (4)
O1D—C14D—C15D—N2D	−178.8 (2)	C18H—C19H—C20H—C21H	0.5 (5)
N1D—C14D—C15D—O2D	−177.3 (2)	C19H—C20H—C21H—C16H	0.6 (5)
O1D—C14D—C15D—O2D	3.2 (3)	N2H—C16H—C21H—C20H	178.0 (3)
C15D—N2D—C16D—C17D	−3.1 (3)	C17H—C16H—C21H—C20H	−1.8 (4)
C15D—N2D—C16D—C21D	175.5 (2)	C6R—C1R—C2R—C3R	0.1 (4)
C14D—N1D—C17D—C18D	−177.2 (2)	C1R—C2R—C3R—C4R	0.5 (4)
C14D—N1D—C17D—C16D	0.8 (3)	C2R—C3R—C4R—C5R	−1.0 (4)
N2D—C16D—C17D—N1D	1.8 (4)	C2R—C3R—C4R—C7R	177.3 (3)
C21D—C16D—C17D—N1D	−176.7 (2)	C3R—C4R—C5R—C6R	0.9 (4)
N2D—C16D—C17D—C18D	179.9 (2)	C7R—C4R—C5R—C6R	−177.5 (3)
C21D—C16D—C17D—C18D	1.3 (4)	C4R—C5R—C6R—C1R	−0.2 (4)
N1D—C17D—C18D—C19D	176.0 (2)	C2R—C1R—C6R—C5R	−0.3 (4)
C16D—C17D—C18D—C19D	−2.1 (4)	C6S—C1S—C2S—C3S	−0.4 (6)
C17D—C18D—C19D—C20D	0.6 (4)	C1S—C2S—C3S—C4S	−0.5 (5)
C18D—C19D—C20D—C21D	1.6 (4)	C2S—C3S—C4S—C5S	0.3 (4)
C19D—C20D—C21D—C16D	−2.4 (4)	C2S—C3S—C4S—C7S	179.6 (3)
N2D—C16D—C21D—C20D	−177.7 (2)	C3S—C4S—C5S—C6S	0.7 (4)
C17D—C16D—C21D—C20D	0.9 (4)	C7S—C4S—C5S—C6S	−178.6 (3)
C6E—C1E—C2E—C3E	−0.4 (3)	C2S—C1S—C6S—C5S	1.4 (6)
C6E—C1E—C2E—C7E	179.7 (2)	C4S—C5S—C6S—C1S	−1.6 (6)
C1E—C2E—C3E—C4E	2.4 (3)	C6T—C1T—C2T—C3T	0.1 (5)
C7E—C2E—C3E—C4E	−177.7 (2)	C1T—C2T—C3T—C4T	0.4 (5)
C1E—C2E—C3E—O1E	−175.86 (18)	C2T—C3T—C4T—C5T	−1.0 (4)
C7E—C2E—C3E—O1E	4.0 (3)	C2T—C3T—C4T—C7T	177.8 (3)
C14E—O1E—C3E—C2E	−110.4 (2)	C3T—C4T—C5T—C6T	1.0 (4)
C14E—O1E—C3E—C4E	71.3 (3)	C7T—C4T—C5T—C6T	−177.8 (3)
C2E—C3E—C4E—C5E	−1.8 (3)	C4T—C5T—C6T—C1T	−0.5 (4)
O1E—C3E—C4E—C5E	176.42 (19)	C2T—C1T—C6T—C5T	−0.1 (5)
C3E—C4E—C5E—C6E	−0.8 (3)	C6U—C1U—C2U—C3U	0.2 (7)
C3E—C4E—C5E—O2H	−177.7 (2)	C1U—C2U—C3U—C4U	0.2 (6)
C15H—O2H—C5E—C4E	−78.5 (3)	C2U—C3U—C4U—C5U	−0.3 (5)
C15H—O2H—C5E—C6E	104.5 (2)	C2U—C3U—C4U—C7U	−179.6 (3)
C4E—C5E—C6E—C1E	2.6 (3)	C3U—C4U—C5U—C6U	0.1 (5)
O2H—C5E—C6E—C1E	179.48 (19)	C7U—C4U—C5U—C6U	179.4 (3)
C4E—C5E—C6E—C7H	−174.4 (2)	C2U—C1U—C6U—C5U	−0.5 (7)
O2H—C5E—C6E—C7H	2.5 (3)	C4U—C5U—C6U—C1U	0.3 (6)

### Hydrogen-bond geometry (Å, º)

C*g*1 is the centroid of the ring C1*D*–C6*D* and C*g*2 is the centroid of the ring C1*H*–C6*H*.

**Table:**

*D*—H···*A*	*D*—H	H···*A*	*D*···*A*	*D*—H···*A*
C2*R*—H2*R*···C*g*1	0.95	2.60	3.532 (1)	166
C6*T*—H6*T*···C*g*2	0.95	2.63	3.566 (2)	169
C2*T*—H2*T*···N2*E*	0.95	2.88	3.734 (2)	150
C1*A*—H1*A*···N1*S*	0.95	2.76	3.693 (2)	169
C1*B*—H1*B*···N1*S*	0.95	2.79	3.742 (3)	176
C1*C*—H1*C*···N1*S*	0.95	2.79	3.714 (2)	166
C1*D*—H1*D*···N1*S*	0.95	2.83	3.775 (1)	173
C1*E*—H1*E*···N1*U*^i^	0.95	2.85	3.784 (1)	169
C1*F*—H1*F*···N1*U*^i^	0.95	2.85	3.798 (2)	174
C1*G*—H1*G*···N1*U*^i^	0.95	2.73	3.673 (3)	171
C1*H*—H1*H*···N1*U*^i^	0.95	2.80	3.752 (2)	175
C1*S*—H1*S*···N1*R*^ii^	0.95	2.57	3.304 (1)	134
C1*U*—H1*U*···N1*T*	0.95	2.74	3.313 (2)	120
C6*U*—H6*U*···N1*T*	0.95	2.78	3.333 (2)	118

Symmetry codes: (i) *x*, *y*−1, *z*; (ii) *x*, *y*+1, *z*.

## References

[bb1] Allen, F. H., Johnson, O., Shields, G. P., Smith, B. R. & Towler, M. (2004). *J. Appl. Cryst.* **37**, 335–338.

[bb2] Amorini, M., Riboni, N., Pesenti, L., Dini, V., Pedrini, A., Massera, C., Gualandi, C., Bianchi, F., Pinalli, R. & Dalcanale, E. (2022). *Small*, **18**, 2104946. 10.1002/smll.202104946 34755446

[bb3] Aprile, A., Palermo, G., De Luca, A., Pinalli, R., Dalcanale, E. & Pagliusi, P. (2018). *RSC Adv.* **8**, 16314–16318. 10.1039/c8ra02875c PMC9080272 35542228

[bb4] Azov, V. A., Beeby, A., Cacciarini, M., Cheetham, A. G., Diederich, F., Frei, M., Gimzewski, J. K., Gramlich, V., Hecht, B., Jaun, B., Latychevskaia, T., Lieb, A., Lill, Y., Marotti, F., Schlegel, A., Schlittler, R. R., Skinner, P. J., Seiler, P. & Yamakoshi, Y. (2006). *Adv. Funct. Mater.* **16**, 147–156.

[bb33] Azov, V., Skinner, P., Yamakoshi, Y., Seiler, P., Gramlich, V. & Diederich, F. (2003). *Helv. Chim. Acta*, **86**, 3648–3670.

[bb5] Ballistreri, F. P., Brancatelli, G., Demitri, N., Geremia, S., Guldi, D. M., Melchionna, M., Pappalardo, A., Prato, M., Tomaselli, G. A. & Trusso Sfrazzetto, G. (2016). *Supramol. Chem.* **28**, 601–607.

[bb6] Bianchi, F., Bedini, A., Riboni, N., Pinalli, R., Gregori, A., Sidisky, L., Dalcanale, E. & Careri, M. (2014). *Anal. Chem.* **86**, 10646–10652. 10.1021/ac5025045 25303228

[bb7] Brighenti, R., Artoni, F., Vernerey, F., Torelli, M., Pedrini, A., Domenichelli, I. & Dalcanale, E. (2018). *J. Mech. Phys. Solids*, **113**, 65–81.

[bb8] Bruker (2016). *APEX3* and *SAINT*. Bruker AXS, Madison, Wisconsin, USA.

[bb9] Clément, P., Korom, S., Struzzi, C., Parra, E. J., Bittencourt, C., Ballester, P. & Llobet, E. (2015). *Adv. Funct. Mater.* **25**, 4011–4020.

[bb10] Farrugia, L. J. (2012). *J. Appl. Cryst.* **45**, 849–854.

[bb11] Frei, M., Marotti, F. & Diederich, F. (2004). *Chem. Commun.* pp. 1362–1363. 10.1039/b405331a 15179465

[bb12] Giannetto, M., Pedrini, A., Fortunati, S., Brando, D., Milano, S., Massera, C., Tatti, R., Verucchi, R., Careri, M., Dalcanale, E. & Pinalli, R. (2018). *Sens. Actuators B Chem.* **276**, 340–348.

[bb13] Groom, C. R., Bruno, I. J., Lightfoot, M. P. & Ward, S. C. (2016). *Acta Cryst.* B**72**, 171–179. 10.1107/S2052520616003954 PMC4822653 27048719

[bb14] Krause, L., Herbst-Irmer, R., Sheldrick, G. M. & Stalke, D. (2015). *J. Appl. Cryst.* **48**, 3–10. 10.1107/S1600576714022985 PMC4453166 26089746

[bb15] Macrae, C. F., Sovago, I., Cottrell, S. J., Galek, P. T. A., McCabe, P., Pidcock, E., Platings, M., Shields, G. P., Stevens, J. S., Towler, M. & Wood, P. A. (2020). *J. Appl. Cryst.* **53**, 226–235. 10.1107/S1600576719014092 PMC6998782 32047413

[bb16] Marsh, R. E. (2004). *Acta Cryst.* B**60**, 252–253. 10.1107/S0108768104003878 15017100

[bb17] Milić, J. V. & Diederich, F. (2019). *Chem. Eur. J.* **25**, 8440–8452. 10.1002/chem.201900852 31111578

[bb18] Moran, J. R., Ericson, J. L., Dalcanale, E., Bryant, J. A., Knobler, C. B. & Cram, D. J. (1991). *J. Am. Chem. Soc.* **113**, 5707–5714.

[bb19] Moran, J. R., Karbach, S. & Cram, D. J. (1982). *J. Am. Chem. Soc.* **104**, 5826–5828.

[bb20] Pinalli, R., Barboza, T., Bianchi, F., Massera, C., Ugozzoli, F. & Dalcanale, E. (2013). *Supramol. Chem.* **25**, 682–687.

[bb21] Pochorovski, I. & Diederich, F. (2014). *Acc. Chem. Res.* **47**, 2096–2105. 10.1021/ar500104k 24814219

[bb22] Portone, F., Amorini, M., Montanari, M., Pinalli, R., Pedrini, A., Verucchi, R., Brighenti, R. & Dalcanale, E. (2023). *Adv. Funct. Mater.* **33**, 2307605.

[bb23] Sheldrick, G. M. (2015*a*). *Acta Cryst.* A**71**, 3–8.

[bb24] Sheldrick, G. M. (2015*b*). *Acta Cryst.* C**71**, 3–8.

[bb25] Skinner, P. J., Cheetham, A. G., Beeby, A., Gramlich, V. & Diederich, F. (2001). *Helv. Chim. Acta*, **84**, 2146–2153.

[bb26] Soncini, P., Bonsignore, S., Dalcanale, E. & Ugozzoli, F. (1992). *J. Org. Chem.* **57**, 4608–4612.

[bb27] Torelli, M., Terenziani, F., Pedrini, A., Guagnini, F., Domenichelli, I., Massera, C. & Dalcanale, E. (2020). *ChemistryOpen*, **9**, 261–268. 10.1002/open.201900345 PMC7043258 32128296

[bb28] Trzciński, J., Pinalli, R., Riboni, N., Pedrini, A., Bianchi, F., Zampolli, S., Elmi, I., Massera, C., Ugozzoli, F. & Dalcanale, E. (2017). *ACS Sens.* **2**, 590–598. 10.1021/acssensors.7b00110 28723190

[bb29] Vincenti, M., Pelizzetti, E., Dalcanale, E. & Soncini, P. (1993). *Pure Appl. Chem.* **65**, 1507–1512.

[bb30] Wagner, G., Arion, V. B., Brecker, L., Krantz, C., Mieusset, J.-L. & Brinker, U. H. (2009). *Org. Lett.* **11**, 3056–3058. 10.1021/ol901122h 19537769

[bb31] Westrip, S. P. (2010). *J. Appl. Cryst.* **43**, 920–925.

[bb32] Zhu, Y.-J., Zhao, M.-K., Rebek, J. Jr & Yu, Y. (2022). *ChemistryOpen*, **11**, e202200026. 10.1002/open.202200026 PMC9197774 35701378

